# Pyridinium amidate (PYA) substituents impact ligand-centered hydride formation and (catalytic) hydride transfer reactivity

**DOI:** 10.1039/d6dt01322h

**Published:** 2026-06-29

**Authors:** Laura Monte, Nicolas Lentz, Martin Albrecht

**Affiliations:** a Department of Chemistry, Biochemistry, and Pharmaceutical Sciences, University of Bern Freiestrasse 3 3012 Bern Switzerland martin.albrecht@unibe.ch

## Abstract

Iridium complexes with pyridinium amidate (PYA) ligands reversibly bind hydrides on the PYA ligand rather than at the iridium center, thus enabling hydride storage and release akin to NAD^+^/NADH. To probe the factors underlying this reactivity, a series of nine operationally unsaturated iridium(iii) complexes containing a *O*,*N*-bidentate phenolate PYA ligand [IrCp*(O^N_PYA_)] were synthesized with different substituents either on the phenolate (H, Me, OMe, NO_2_) or on the PYA unit (H, CN, CONH_2_, NHCOMe, NH_2_). Crystallographic, UV-vis spectroscopic and DFT analyses indicate electronic decoupling of the pyridinium and aminophenolate units of the ligand. Upon reaction with formate, all complexes but the NH_2_-substituted derivative gave dihydropyridine products due to hydride storage either at the pyridinium C4 (*para*) or C6 (*ortho*) position. The *ortho*/*para* ratio was governed by the pyridinium substitution, with electron-withdrawing substituents favoring the *ortho* product, while the electron-donating NH_2_ substituent suppressed hydride formation completely. In contrast, aminophenolate substitution did not affect the regioselectivity and yielded *para* hydrides. The impact of aminophenolate and PYA substituents on the regioselectivity of *ortho*/*para* hydride formation was further rationalized by Löwdin population analysis. When probed in catalytic transfer hydrogenation, the complexes showed substituent-dependent activity, though remarkably little correlation with regioselectivity of the hydride formation nor with hydride stability as probed by reaction with benzoic acid. The most active complex containing an aminophenolate methyl substituent reached *k*_obs_ = 7.0 h^−1^. The catalytic activity trend correlates with the aminophenolate substituent effects, but not with pyridinium substitution nor HOMO–LUMO gap. These results thus disentangle distinct ligand effects, with the PYA unit governing hydride regioselectivity and stability, while the aminophenolate unit impacts the iridium electron density and catalytic activity.

## Abstract 2

Ligand substituents in iridium PYA complexes are demonstrated to tailor either iridium electron density and catalytic activity, or orthogonally the storage of hydrides on the pyridinium unit in terms of stability and *ortho*/*para*-selectivity.

## Graphical abstract

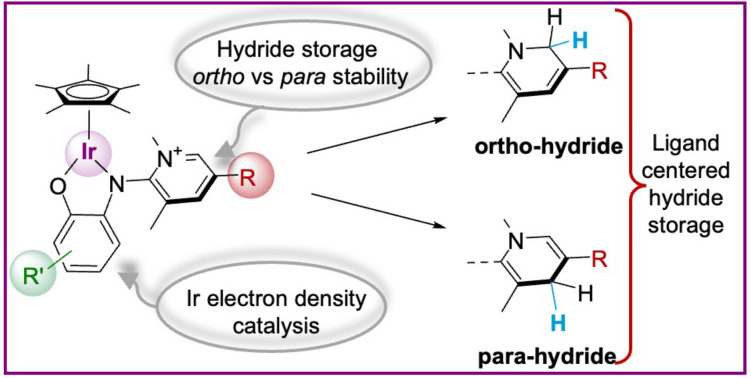

## Introduction

**Fig. 1:**
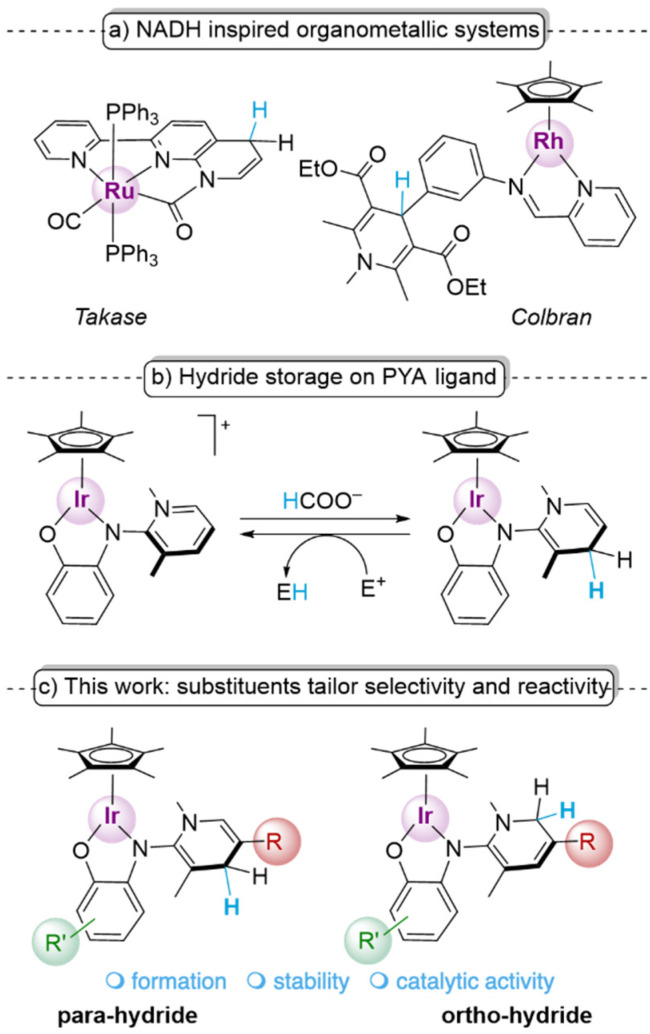
Ligand-centered hydride storage in organometallic complexes: (a) representative Ru and Rh complexes, (b) iridium PYA systems with reversible ligand-centered hydride storage and release, (c) substituent effects influence selectivity and stability of hydride storage.

Classic transition metal-catalyzed hydrogenations feature a metal hydride as the key reactive intermediate for hydrogen transfer to a substrate.^1–6^ In contrast, enzymes rely on the NAD^+^/NADH pair and related cofactors as central vector for hydride transfer in biological systems, as the pyridinium ring in NAD^+^ reversibly stores a hydride *via* aromaticity loss.^7–9^ Inspired by this orthogonal hydride transfer pathways, organometallic complexes bearing NADH-mimicking ligands have been developed that combine metal-centered reactivity with ligand-centered reversible de- and re-aromatization for hydride storage and release. Early work by Fujita showed that Ru complexes containing a pyridine–pyridinium scaffold photochemically generate dihydropyridines by ligand-based hydride binding.^10^ Using a similar ligand, Tanaka and co-workers described hydride transfer from Ru and Rh complexes onto the ligand.^10,11^ More recently, Takase revealed a Ru complex bearing a pyridyl-azaacridine ligand, which is selectively hydrogenated on the pyridine moiety (Fig. 1a).^12^ Similarly, Colbran developed Rh complexes with a Hantzsch ester fragment^13,14^ tethered to an iminopyridine ligand, which triggered intramolecular hydride transfer between the metal and the ligand and afforded stable ligand-bound hydrides (Fig. 1a).^15,16^

Several families of complexes exhibit hydride reactivity at ligands also beyond pyridinium-type systems. For example, Waymouth reported cobalt–azopyridine complexes that reductively bind hydrogen at the azo moiety to generate reactive N–H functionalities as hydride donors.^17^ Heyduk demonstrated nickel–ONO complexes capable of transferring hydrides localized on the redox-active ligand,^18^ and Radosevich devised ruthenium–phosphorane complexes in which hydride storage occurs at the phosphorus.^19^

We recently discovered that donor-flexible pyridinium amidate (PYA) ligands^20–24^ bound to iridium(iii) react with formate to release CO_2_ and form a hydride that is stored *via* pyridinium ring dearomatization (Fig. 1b),^25^ reminiscent to the reactivity of NAD^+^. The ligand-bound hydride is stable in the presence of air and moisture, yet it reacts readily with electrophiles. Moreover, its formation and consumption are fully reversible, which provided access to catalytic turnover. We hypothesized that careful selection of different substituents on the ligand influences the stability and reactivity of the hydride, thus providing valuable mechanistic insights. Here we have introduced substituents at key positions of the PYA ligand as a methodology to shed light on the factors governing the formation, reactivity, and catalytic activity of these ligand-bound hydride systems. These studies unveil diverging electronic effects of substituents at the pyridinium unit compared to modification of the aminophenolate unit, with direct consequences on the reactivity, including an interplay of metal-hydride, and different ligand-bound hydrides. Specifically, we have identified hydride binding both in *ortho* and in *para* position of the pyridinium fragment (Fig. 1c) as well as mutual isomerization, which suggests that hydride reactivity is metal-based, though storage is mostly ligand-centered.

## Results and discussion

### Synthesis of iridium complexes

**Scheme 1:**
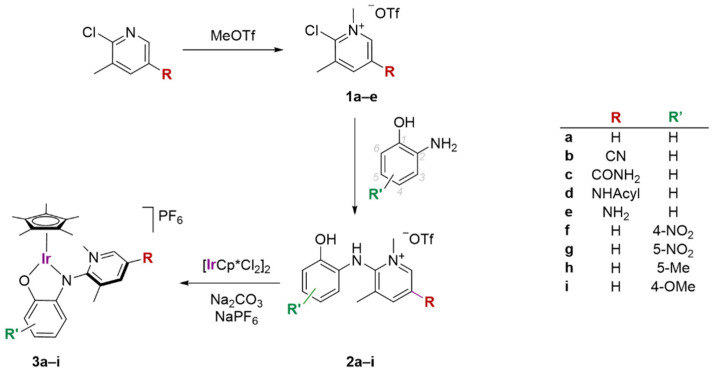
Synthesis of complexes 3a–3i from the appropriate chloro-pyridine precursors involving formation of pyridinium salts 1a–e, aminophenolate condensation to afford the ligand precursors 2a–i, and final base-assisted iridation.

Iridium complexes with substituents either at the aminophenolate or the pyridinium amidate unit were synthesized from commercially available or synthetically readily accessible 2-chloropyridines. Thus, the chloro-pyridinium triflate salts 1 were prepared by reacting the appropriately substituted pyridine with methyl triflate (Scheme 1). Condensation of these chloro-pyridinium salts with the (un)substituted 1,2-aminophenol in MeCN using NaHCO_3_ as a base gave the ligand precursors 2a–i. The less electrophilic amine-functionalized pyridinium salt 1e required NEt_3_ as a slightly stronger base. Complexation of the ligand precursors was accomplished with [IrCl_2_Cp*]_2_ in MeCN in the presence of Na_2_CO_3_ and NaPF_6_ and yielded complexes 3a–i as air- and moisture-stable orange/red solids.

All complexes were characterized by ^1^H and ^13^C NMR spectroscopy, HR-MS and elemental analysis. The NMR spectra of the new complexes feature general resonance patterns similar to 3a, therefore suggesting a similar two-legged piano stool geometry as determined for 3a.^25^ Comparison of the ^1^H NMR chemical shifts across the series indicates distinct electronic substituent effects. Specifically, pyridinium modification exerts little impact on the resonance of the aminophenolate hydrogen resonances (all chemical shifts ±0.02 ppm from those of 3a), though a pronounced effect was noted for pyridinium proton H4_pyr_ which shifts from *δ*_H_ = 8.76 in 3a to 9.12 (3b), 8.81 (3c), 8.44 (3d), and 7.70 (3e). The latter reflects the strongly electron-donating nature of the NH_2_ substituent in 3e, which also induced a substantial upfield shift of the pyridinium H6_pyr_ resonance from *δ*_H_ = 9.04–9.69 ppm in complexes 3a–d to *δ*_H_ = 7.83 in 3e. Apart from complex 3e, the chemical shifts do not correlate with the Hammett parameter of the substituents. A stronger correlation was observed upon aminophenolate modification. Complexes 3f and 3g with electron-withdrawing nitro substituents feature a downfield shift of the phenolate hydrogen H6_phen_ at *δ*_H_ = 7.54 and 7.89, respectively, compared to 7.16 for 3a, while electron-donating Me and OMe groups induce an upfield shift with resonances at 6.94 ppm for 3h and 7.00 ppm for 3i. Notably, an upfield shift relative to 3a was also observed for the pyridinium protons. The magnitude of this shift is weakly correlated to the substituent Hammett parameter (*e.g.* for H6_pyr_*δ*_H_ = 8.64, 8.64, 8.58 and 8.59 for 3f, 3g, 3h, and 3i, respectively). This correlation may suggest a weak electronic coupling of the aminophenolate and pyridinium electronic systems, though the absence of any reverse inductive effects of pyridinium substituents on aminophenolate hydrogen shifts points to the contrary and fully decoupled systems.

### Crystallographic analysis

**Fig. 2:**
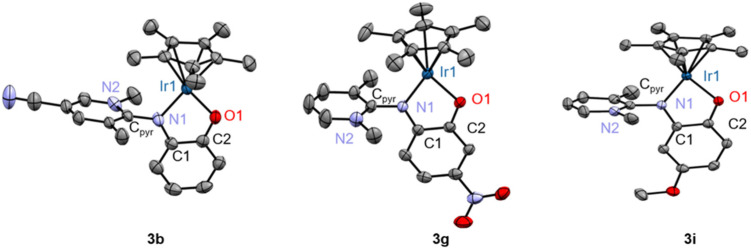
Thermal ellipsoid plots of the molecular structures of 3b, 3g, and 3i (50% probability level, H atoms, PF_6_^−^ anion, and co-crystallized solvent molecules omitted for clarity).

Complexes 3b, 3g, and 3i were selected as representatives for solid state analysis. The complexes were crystallized by slow diffusion of Et_2_O into a solution of the complex in a mixture of MeCN and CH_2_Cl_2_. All molecular structures show two-legged piano-stool structures^26–30^ with the iridium center coordinated to a Cp* ligand and the *N*,*O*-bidentate phenolate-PYA ligand (Fig. 2). As a consequence of this geometry, the pyramidalization angle *α*,^28^ defined by the angle of the O–Ir–N plane with the axis from iridium to the Cp* centroid, is essentially linear at 180°, which also excludes any stabilizing agostic interactions.^31^ The length of the Ir–N1 bonds (1.99(1) Å) and in particular of the Ir–O1 bonds (2.004(5) Å) are identical within all complexes, indicating no significant differences upon ligand substitution (Table 1). Both the N1–C1 and N1–C_pyr_ bonds consistently show partial double bond character in all complexes, with bond lengths between 1.37–1.40 Å. The sum of the angles around N1 is 360° (∑∠N1), indicating a planar, sp^2^-hybridized nitrogen and the absence of pyramidalization due to partial sp^3^ hybridization. These structural features are consistent with π-delocalization of the nitrogen lone pair. Similarly, the O1–C2 bond reveals partial double bond character with a bond length in the 1.32–1.35 Å range.^32^ In all complexes, the pyridinium and aminophenolate rings are quasi perpendicular to each other with dihedral angles *θ* between 80.63° and 89.91°. Such a strong tilting further supports the lack of π interactions between the pyridinium ring and the N donor site.

**Table 1:** Selected bond lengths and angles for complexes 3a, 3b, 3g, 3i

	3a^a^	3b	3g	3i
Ir1–O1	2.009(5)	2.003(3)	2.010(4)	1.999(2)
Ir1–N1	1.982(7)	2.001(3)	1.987(4)	2.001(3)
N1–C1	1.404(10)	1.412(5)	1.388(6)	1.394(3)
N1–C_pyr_	1.386(10)	1.376(5)	1.402(7)	1.397(3)
O1–C2	1.351(8)	1.343(5)	1.327(5)	1.348(3)
*θ* b	89.26	80.63	89.61	88.17
*α* c	179.44	178.72	179.67	178.69
∑∠N1	360	359	360	360

a Data for 3a from ref. 25.

b Dihedral angle between phenolate and pyridinium planes.

c Pyramidalization angle between N–Ir–O and the Ir–Cp_centroid_ axis (see ref. 28).

### UV-vis spectroscopic analysis

**Fig. 3:**
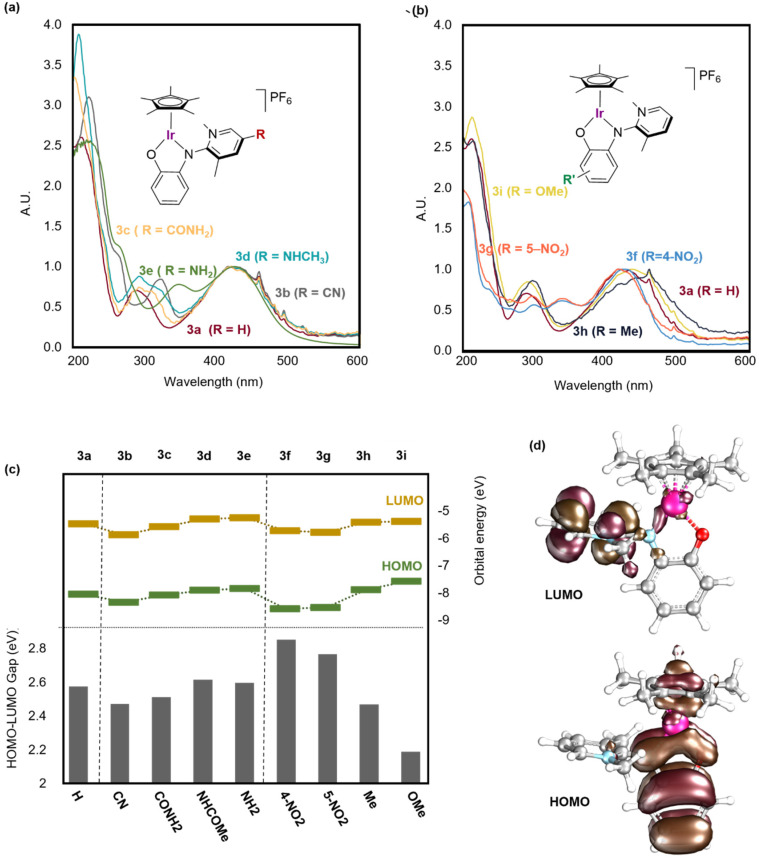
Superimposed UV-Vis spectra of (a) pyridinium modified complexes 3a–3e and (b) aminophenolate modified complexes 3a, 3f–3i, all in MeCN solution; (c) computed HOMO–LUMO energy levels and energy gap for complexes 3a–3i; (d) orbital visualization of HOMO and LUMO for the representative complex 3a, indicating the involvement of the iridium aminophenolate in the HOMO and the pyridinium site in the LUMO.

The electronic structure of the complexes was further investigated by UV-Vis spectroscopy. The spectra of all complexes 3a–i display two main absorption regimes, *viz.* two ligand-centered π → π* transition around 230 nm and 300 nm, and a charge-transfer (CT) band in the 420–450 nm range. The CT band is essentially independent of the electronic nature of the substituents on the pyridinium ring with a maximum at about 420 nm regardless of whether the substituent is electron-withdrawing (3b, 3c) or electron-donating (3d, 3e; Fig. 3a). Conversely, complexes with modified phenolate rings reveal a substituent-dependent shift of the absorption maximum *λ*_max_. Complexes bearing electron-donating groups (3h, 3i) feature a red shift relative to 3a with *λ*_max_ = 431 and 447 nm, respectively, whereas electron-withdrawing-substituents induce a blue shift, (*λ*_max_ = 415 nm for 3f, 421 nm for 3g; Fig. 3b). This trend may suggest a ligand-to-metal charge transfer (LMCT), with a smaller energy gap as the ligand donor properties increase. Additionally, it suggests that the orthogonality of the two aromatic rings in the PYA phenolate ligand imposes an electronic barrier between the pyridinium unit and the first coordination sphere of iridium, thus preventing pyridine modification to influence the CT bands. Unsurprisingly, the bands associated with ligand-centered transitions (220–310 nm) are affected by both types of modifications.

To identify the transition responsible for this absorption, ground-state DFT calculations were performed at the B3LYP^33,34^-D3BJ^35,36^/def2-TZVP^37,38^ level with a continuous solvent model CPCM(MeCN).^38–40^ Geometry optimization followed by single-point orbital energy calculations yielded HOMO–LUMO gaps across the series. The energy gap follows a trend qualitatively similar to the *σ*_*para*_ Hammett parameters of the *para*-substituents (Fig. S1), although the correlation is not strictly linear. The calculations confirm that modification of the pyridinium has only a minor impact on the HOMO–LUMO gap, while phenolate modifications exhibit a much more substantial effect (Fig. 3c). While both types of ligand modifications shift the HOMO and LUMO energies, the phenolate modification has a more pronounced impact on the HOMO energy and hence modulates the gap stronger.

Analysis of the frontier orbitals showed a metal-centered HOMO, while the LUMO has predominantly pyridine π* character (Fig. 3d). Modification of the substituents on the aminophenolate unit therefore impacts predominantly the energy level of the HOMO. Conversely, substituent changes on the pyridinium ring directly influences the π* LUMO by redirecting electron density around the heterocycle. However, modifications on the pyridinium ring are less effective and modulate the HOMO–LUMO gap only little, in agreement with the essentially unchanged CT bands in the UV-vis spectra. Moreover, TD-DFT calculations revealed that the dominant excitation at around 430 nm is a HOMO → LUMO transition, thus rationalizing the correlation of the HOMO–LUMO gap energies with the absorption features in the visible region (Fig. S2).

### Electrochemical analysis of selected complexes

**Fig. 4:**
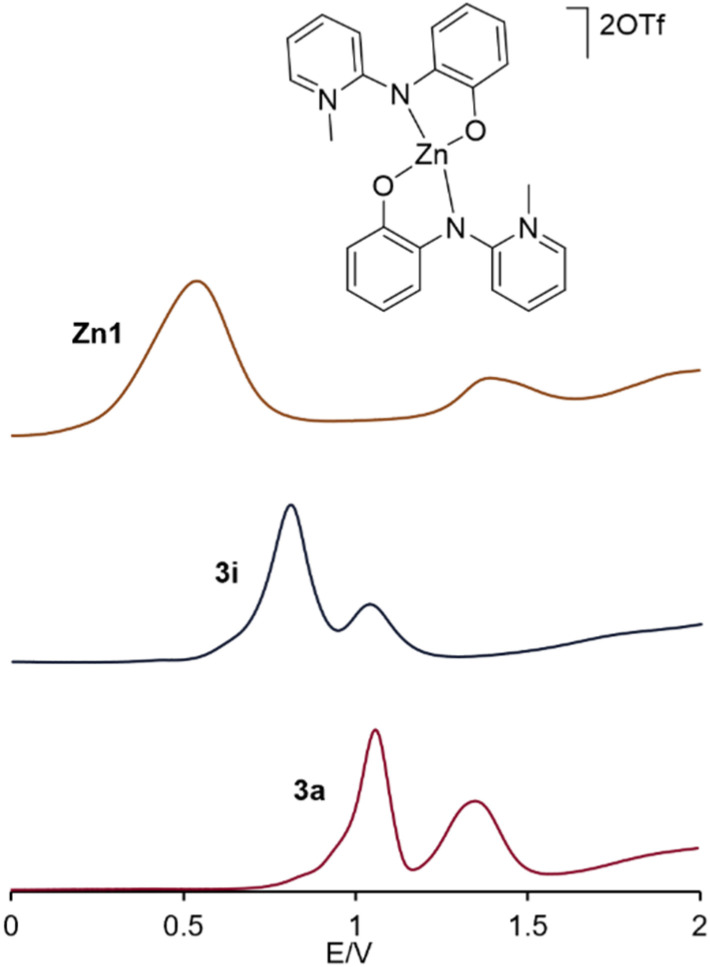
Stacked normal-pulse voltammograms (NPV) of complexes 3a, 3i, and Zn1 (in CH_2_Cl_2_, potentials *vs.* Fc^+^/Fc, with (Bu_4_N)PF_6_ as supporting electrolyte), together with the molecular structure of Zn1.

The electrochemical properties were investigated by cyclic voltammetry (CV) and normal-pulse voltammetry (NPV) experiments on selected complexes. CV scans in the 0 to +2.0 V potential range (*vs.* Fc^+^/Fc in CH_2_Cl_2_ using (Bu_4_N)PF_6_ as supporting electrolyte) revealed three distinct oxidation processes for complex 3a at +0.8, +1.05 V, and +1.35 V, the latter two as reversible processes according to NPV analysis. The relative area of these two oxidations suggests both to be one-electron processes (Fig. S3a). To better assign these processes, we synthesized the Zn^II^ complex Zn1 with a redox-innocent metal center.^14^ The CV and NPV plots of complex Zn1 feature a reversible redox process at 0.55 V, indicative of a redox-active ligand (Fig. 4 and Fig. S3b).^41–46^ Given the similarities of the redox behavior of the two ligands, we attribute one of the reversible oxidations of 3a to a ligand-centered phenolate/phenoxyl oxidation^47–50^ and the other one to a metal-centered Ir^III^/Ir^IV^ process, though the available data do not allow to distinguish which of the two processes occurs at lower potential. To shed further light on this redox behavior, complexes 3f and 3i were analyzed as they contain an electron-withdrawing nitro group and an electron-donating methoxy group, respectively. The nitro-substituted complex 3f shifts both oxidations anodically (*E*_1/2_ = 1.39 V and *E*_1/2_ > 2 V) relative to 3a (*E*_1/2_ = 1.05 V and *E*_1/2_ = 1.35 V). Conversely, electron-donating MeO substituent in complex 3i shifted both processes to lower potential (*E*_1/2_ = 0.81 V and *E*_1/2_ = 1.05 V), as expected for processes involving the phenolate and the iridium metal center (Fig. 4 and Fig. S3d). These results also align with previous studies on pyridylidene-amine Ir^III^ complexes, for which the oxidation potential linearly correlates with the Hammett *σ*_p_ parameter of the substituent.^51^

### Hydride storage

**Scheme 2:**
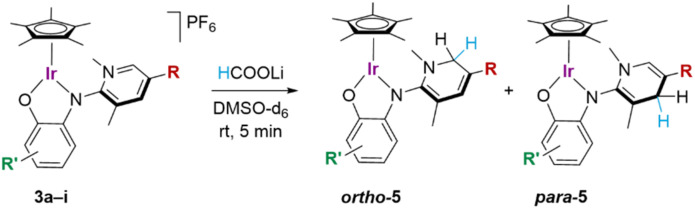
Hydride storage on the ligand upon reaction of complexes 3a–i with HCOOLi (10 eq.) in DMSO-d_6_, yielding ***ortho***- and *para*-5a–i in different ratios (see Table 2).

Complex 3a is known to react with formate to undergo pyridinium dearomatization through hydride storage at the pyridinium C4 position.^25^ This reactivity is expected to be strongly affected by ligand substitution effects and was therefore investigated with complexes 3b–3i under standard conditions, that is, with an excess of HCOOLi (10 eq.) as the hydride source in DMSO-d_6_. Characterization of the reaction mixture by ^1^H NMR spectroscopy indeed revealed distinct reactivity patterns. Specifically, for complexes 3b, 3c, and 3d, two sets of signals were identified after formate addition, attributed to the pyridinium C6-bound hydride complex *ortho*-5 as well as the analogous *para*-5 with the hydride bound to the pyridinium C4 (Scheme 2).^52–54^ Key distinction of the two isomers is the characteristic AB doublet for the heterocyclic CH_2_ group, which appeared around 4.1 ppm with a diagnostic 12.3 Hz vicinal coupling in *ortho*-5b, while the doublets are upfield shifted to 3.34 and 3.28 ppm in *para*-5b due to the absence of a heteroatom adjacent to the CH_2_ group, and the coupling constant is larger with ^2^*J*_HH_ = 17.3 Hz (Fig. S4). Additional evidence for the distinct hydride bonding to either C4 or C6 was provided by nuclear Overhauser experiments which revealed spatial correlation between the C4 methylene protons and the C5-bound methyl group at 1.35 ppm in *para*-5b. In contrast *ortho*-5b displays a correlation between the methylene protons and the resonance of the N-bound methyl group at 2.54 ppm (Fig. S5). Similar reactivity patterns were observed in the reaction of 3c and 3d with formate.

The hydride storage in either *ortho* or *para* position depends on the substituent at the pyridinium unit. Without substituents, *ortho*-5a is detectable only in trace amounts (<5%), yet the isomer is increasing in relative ratio with acyl (10% *ortho*-5c), cyano (15% *ortho*-5b), and *N*-acyl substituents (26% *ortho*-5d; Table 2, entries 1–4). The larger ratio of *ortho*-isomer in 5b and 5c compared to 5a suggest a direct role of electron-withdrawing substituents, while in 5d, H-bond stabilization by the acetamide group may favor the *ortho*-product. Interestingly, complex 3e with a strongly electron-donating amine substituent at the pyridinium ring was inert towards formate, indicating that electron-rich substituents prevent the pyridinium heterocycle to accept hydrides. In contrast, phenolate-modified complexes 3f–i yielded the *para*-isomers exclusively with no *ortho*-5 formed apart from nitro-functionalized 3g, which gave traces of *ortho*-5g (<5%). The methylene complexes *para*-5f–i were characterized by the diagnostic AB doublets at 3.18 ppm and 3.27 ppm with large 18–21 Hz coupling constants as well as ^1^H–^1^H NOESY correlations between H6 and the *N*-methyl CH_3_ group, and between the methylene hydrogens bound to C4 and the C5-methyl group. Notably, reaction of 3f and 3g—both containing electron-withdrawing nitro substituents—also produced trace amounts (<5%) of several metal hydride species upon reaction with formate, indicated by small but detectable resonances in the negative ppm region (−11 to −15 ppm). No transient iridium-hydride species was detected with any of the complexes when monitoring the reaction within the first minutes.

**Table 2:** *ortho*-5*vs.para*-5 product distribution upon reaction of complexes 3a–i with formate^a^

Complex	*ortho*-5	*para*-5
3a	<5%	95%
3b	15%	85%
3c	10%	90%
3d	26%	73%
3e	n.d.	n.d.
3f	n.d.	>95%^b^
3g	<5%	>95%^b^
3h	n.d.	>95%
3i	n.d.	>95%

a Reaction conditions as in Scheme 2, products quantified by integration of the pyridinium H4 and H6 ^1^H NMR resonances, n.d. = not detected.

b Appearance of several minor resonances in the hydridic region, attributed to iridium hydrides (<5% in total).

The substituent dependence of the *ortho*/*para* selectivity of hydride formation supports the notion of an electronic separation between the pyridinium ring and the iridium aminophenolate unit in these complexes. Moreover, an electron-withdrawing substituent on the phenolate ring depletes electron density from the iridium center, which promotes the stabilization of metal hydride species.

**Fig. 5:**
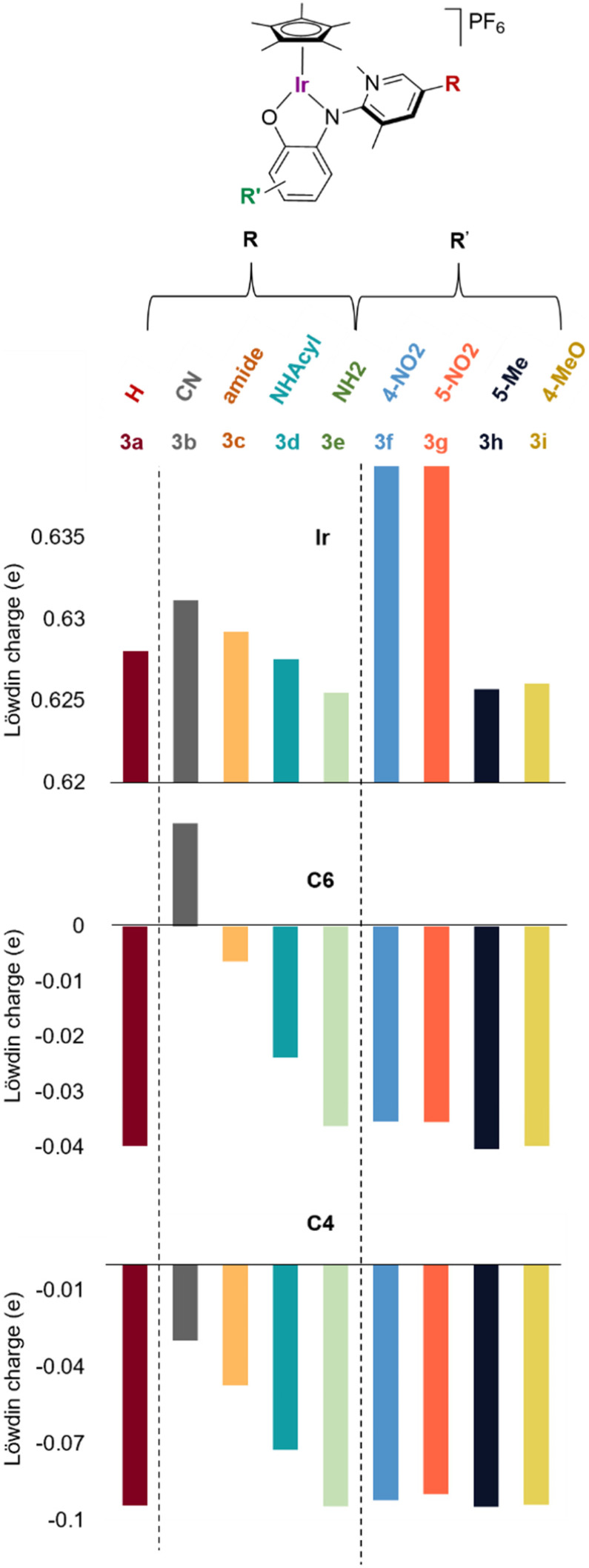
Löwdin population analysis on the optimised DFT geometries (B3LYP-D3(BJ)/def2-TZVP and CPCM(DMSO)) of complexes 3a–i, depicting the substituent-dependent electronic density around (a) the iridium (b) pyridinium C6 (formation of *ortho*-5) and (c) pyridinium C4 (formation of *para*-5).

To elucidate the distinct selectivity preferences for hydride storage at iridium, or pyridinium *ortho vs. para* positions, Löwdin population analyses^55–58^ were performed for all complexes on the DFT optimized geometries (B3LYP-D3(BJ)/def2-TZVP and CPCM (DMSO)). Variations at the pyridinium moiety have only a minor effect on the electron density at the iridium center, as indicated by the small changes in Löwdin population relative to the unmodified parent complex 3a (Fig. 5a). Aminophenolate modulation has a more pronounced effect, in particular electron-donating substituents increase the electron density at iridium, though the overall charge differences remain relatively small with *Δ* around 0.01 units. Analysis of the pyridinium ring revealed a clear and systematic correlation of charge population with the substituents. The Löwdin populations at C4 and C6 decrease progressively as the pyridinium substituent becomes more electron-withdrawing (Fig. 5b and c). For example at the pyridyl C4 site, the population difference to 3a increases from 3d (R = NHCOMe, *Δ*_3d_ = 0.022) to 3c (R = CONH_2_, *Δ*_3c_ = 0.047) and even stronger for 3b (R = CN, *Δ*_3b_ = 0.064). The trend is very similar for C6 with *Δ*_3d_ = 0.016, *Δ*_3c_ = 0.033, and *Δ*_3b_ = 0.058. In contrast, the NH_2_ substitution in 3e imposes essentially no changes. Likewise, substituents in the aminophenolate unit have only a very minor effect on the electron population at the pyridinium carbons (*Δ* < 0.003), again indicating that electronic communication between the phenolate and pyridinium fragments is limited.

**Fig. 6:**
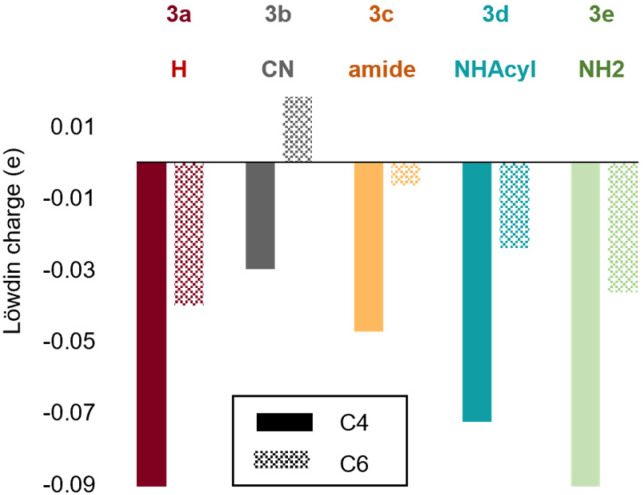
Comparison of Löwdin populations at the pyridinium C4 (solid bars) and C6 (patterned bars) positions for the pyridinium-modified complexes 3a–3e, indicating the preference of C4 (leading to *para*-5) *vs.* C6 (affording the *ortho* isomer).

The Löwdin population qualitatively predicts the regioselectivity of hydride storage, with a much larger coefficient on C4 leading to *para*-5 compared to C6 producing the *ortho* isomer (Fig. 6). However, the actual ratios are not reflected by the Löwdin charge differences which are all in the 0.04–0.05 range, while the *para vs. ortho* ratio varies from 19 : 1 to 3 : 1. These different ratios correspond to about 1 kcal mol^–1^ difference, presumably within the error of the DFT calculations.

Cumulatively, these experiments suggest a pathway involving reversible hydride formation at the iridium center, which is stabilized by electron depletion through incorporation of electron-withdrawing nitro substituents on the aminophenolate unit. In the absence of such stabilizing effects, the hydride migrates to the pyridinium ring to induce dearomatization with a 1,2-(*ortho*) *vs.* 1,4-(*para*) reduction selectivity that is triggered by the pyridinium substituent. Electron-donating NH_2_ substituents do not favor any hydride formation, neither at the metal nor stored on the ligand.

### Hydride reactivity

**Scheme 3:**
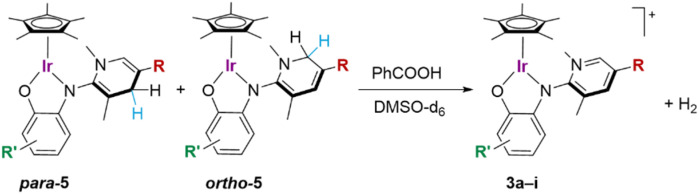
Reactivity of *in situ* generated iridium hydride species 5a–i with an excess benzoic acid (6 eq.) in deuterated DMSO.

To investigate the stability of the hydrides, the *in situ* prepared iridium complexes 5a–i were reacted with benzoic acid (6 eq.) as a proton source, and the depletion of *para*-5 and *ortho*-5 was monitored by ^1^H NMR spectroscopy (Scheme 3). The hydride of the parent unsubstituted complex *para*-5a was consumed over the course of 5 h, while the *ortho*-isomer was detectable much longer and required 10 h to be fully converted. The initial concentration of *ortho*-5a as the minor species was very low from the start, which prevented any accurate quantification of conversion. Interestingly, complex 5d showed an opposite isomer stability with *ortho*-5d fully converted within 8 h, while some 30% *para*-5d remained beyond 12 h. The stabilizing effect of electron-withdrawing pyridinium substituents was even more striking with complexes 5b and 5c. These complexes do not show any reactivity with benzoic acid during 12 h, both as the *ortho*- and *para*-isomers. This inertness correlates with the reduced electron density at C4 and C6 as predicted by Löwdin population analysis, thus stabilizing the hydride much better than in complexes 5a or 5d.

**Fig. 7:**
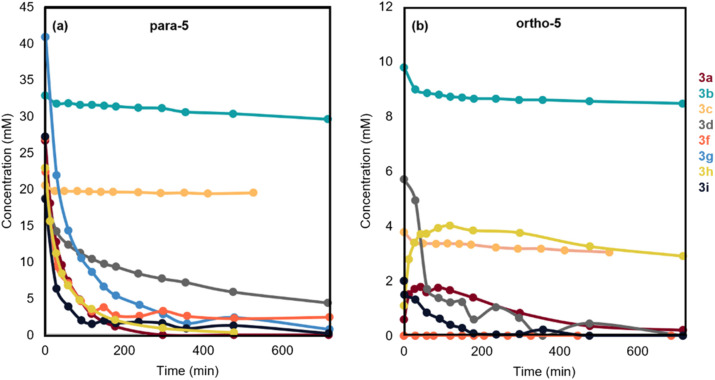
Stability profile of *in situ* generated (a) *para*-5 and (b) *ortho*-5 upon addition of 10 eq. benzoic acid.

Aminophenolate modifications showed no significant effect on the stability of the hydride in complexes 5f–i with conversion rates very similar to those of the parent complex 5a. In all these complexes 5f–i, the *para*-isomer was prevailing with no *ortho*-isomer detectable apart from traces of *ortho*-5g. Remarkably, however, addition of benzoic acid to *para*-5h induced the formation of *ortho*-5h with a concentration increase over the first hour followed by a gradual and slow hydride consumption that is not complete within 12 h. A similar but less pronounced isomerization was observed for 5a, suggesting proton-induced hydride migration (Fig. 7).

While reactions of complexes 5a–i with benzoic acid yielded the original complexes 3a–i as the major products, complexes 5a and 5d also produced a hydride species identified by an upfield resonance at −11.96 ppm, which gradually increased over time. Similarly, reaction of benzoic acid with complexes 5h and 5i yielded two hydridic signals, one of which at the same −11.96 ppm chemical shift. Interestingly, this signal does not correlate with any resonances in the positive region, suggesting the formation of iridium hydride clusters without the PYA ligand. Complexes 5f and 5g with electron-poor metal centers featured several metal hydrides already upon addition of HCOOLi (*vide supra*), and formation of new metal hydrides was observed upon exposure to benzoic acid (Fig. S28), including one resonance at −11.96 ppm in trace amounts (<5%). Since this resonance is independent of the ligand substitution pattern and gradually increases over time for all complexes, it is presumably related to a decomposition product involving Cp* iridium hydride units.^59,60^

### Catalytic activity

**Scheme 4:**
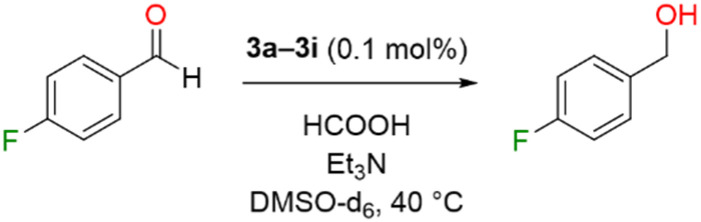
Catalytic hydrogenation of *p*-fluorobenzaldehyde (1 M) in the presence of 0.1 mol% 3a–3i using formic acid (1.2 eq.) and NEt_3_ (1.5 eq.) as additive in DMSO-d_6_ (0.5 mL).

**Fig. 8:**
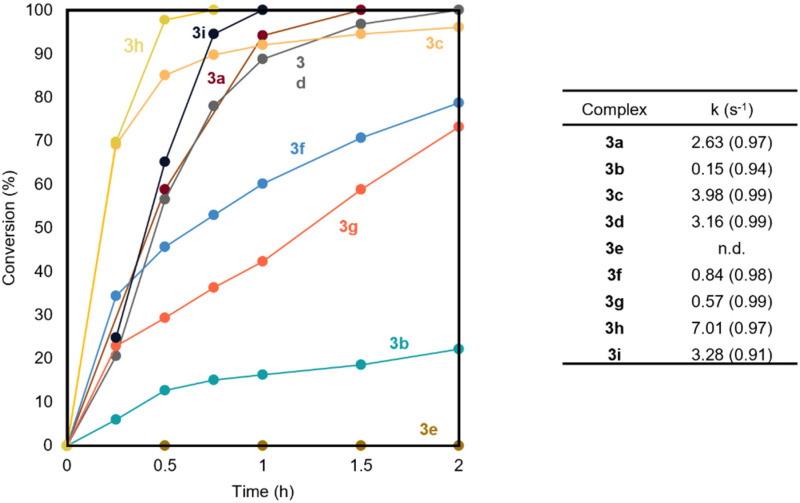
Catalytic profiles of complexes 3a–3i in the transfer hydrogenation of *p*-fluorobenzaldehyde and corresponding rate constants *k*_obs_ (correlation coefficient *R*^2^ in parentheses) assuming pseudo first-order kinetics. General conditions: complex 3 (1 µmol), HCOOH (1.2 mmol), NEt_3_ (1.5 mmol), *p*-fluorobenzaldyhde (1 mmol).

Having assessed the electronic parameters of the complexes and their hydride formation and stability, we aimed at correlating these data with catalytic activity. For this purpose, transfer hydrogenation of 4-fluorobenzaldehyde with formic acid/NEt_3_ as the hydrogen source was used as a model reaction (Scheme 4), allowing for direct comparison with known catalytic systems.^25^ All catalytic runs were performed with 0.1% mol catalyst loading, 1 M substrate concentration, and using a slight excess of formic acid (1.2 eq.) and NEt_3_ (1.5 eq.). Monitoring substrate conversion and product formation revealed a large diversity of activities (Fig. 8). Complex 3e is catalytically inactive, in agreement with the lack of hydride stabilization and no detectable formation of 5e. Alternatively, 5e might be formed transiently, for example, with a largely unfavorable equilibrium constant that prevents detection, though also with an insufficiently long lifetime for catalytic turnover. In contrast, the parent complex 3a reached full conversion within 1.5 h with a rate constant *k*_obs_ = 2.6 h^−1^ assuming first-order kinetics. Pyridinium modification had diverging effects, ranging from poor activity of nitrile-functionalized complex 3b (*k*_obs_ = 0.15 h^−1^), to higher performance of acyl-substituted complex 3c and 3d, both featuring rates exceeding the benchmark complex 3a with *k*_obs_ = 4.0 h^−1^ and 3.2 h^−1^, respectively. This sequence of activity 3b < 3d < 3c is not correlating with hydride stability (3d ≪ 3b, 3c) nor with the HOMO–LUMO gap or electron density at the metal, thus indicating other factors at play. For example, competitive nitrile bonding to the iridium center may prevent substrate coordination and thus lower the activity of 3b. It is remarkable, however, that complex 3c forms a hydride with extended stability towards protonation (*cf.* stability of 5c) yet provides one of the most active complexes of the series.

Modifications at the aminophenolate unit provided a clearer relationship between the electron density at the metal center, the HOMO–LUMO gap, and the catalytic activity. Iridium centers with reduced electron-density as in 3g and 3f were considerably less efficient catalysts than 3a, reaching 30–40% conversion within 30 min with *k*_obs_ = 0.84 h^−1^ and 0.57 h^−1^ respectively. Conversely, electron-donating substituents as in complexes 3i and especially 3h enhance activity with 65% and 98% conversion at the 30 min mark and *k*_obs_ = 3.3 h^−1^ and 7.0 h^−1^, respectively. Notably, these complexes show similar stability for the ligand-stored hydride complexes 5f–5i, though complex *para*-5h revealed an increased hydride mobility through acid-mediated formation of *ortho*-5h. This hydride mobility may be correlated with the enhanced catalytic activity of this complex. Moreover, complexes 3g and 3f form various metal hydrides upon reaction with formate and even more so upon hydride transfer under acidic conditions, thus providing a rationale for the low performance and gradual deactivation of those complexes under catalytic turnover conditions. Catalyst lifetimes differ throughout the series, yet not in a systematic manner. Thus, the conversion profiles indicate a compromised stability of the active species for complex 3c, yet not for 3d and the aminophenolate-substituted complexes 3h and 3i.

## Conclusions

This work discloses synthetic approaches for introducing a variety of substituents into pyridinium aminophenolate ligands. The effect of these substituents on the properties of underligated iridium complexes has been investigated in detail by using solid state X-ray diffraction, solution state NMR and UV-vis spectroscopies, electrochemistry, as well as DFT analysis, including Löwdin population analysis. These analyses suggest electronic separation of the iridium aminophenolate unit from the pyridinium residue of the ligand. Assessing the reactivity of these complexes in the reversible hydride storage *via* dearomatization of the pyridinium site indicates diverging effects of the substituents, revealing in particular a substituent-dependent propensity to store the hydride *ortho* or *para* to the pyridine nitrogen. Moreover, the stability of the hydride towards acid-induced dehydrogenation varies greatly, with electron-withdrawing substituents on the pyridinium unit leading to a very stable hydride. Notably, in some cases, acid-induced isomerization of the hydride from *para*- to *ortho*-position was observed and may hint to a pathway for hydride migration from the iridium center to different positions of the ligand. Löwdin population analysis was shown to be very efficient in predicting favored positions for hydride bonding. Remarkably, catalytic transfer hydrogenation activity, which requires these hydrides to be formed and released, does not correlate with hydride stability and indicates other factors at play. Most notably, substitution of the aminophenolate ligand impacts the iridium electron density as well as charge transfer and catalytic activity in a correlating manner that aligns with Hammett parameters of the substituents. In contrast, pyridinium substitution does not show any linear effect, though provides opportunities to enhance the stability of the ligand stored hydride as well as improving catalytic activity. Our results thus demonstrate the power of fine-tuning electronic properties of the pyridinium amidate framework for optimizing stability and catalytic activity of these iridium complexes.

## Experimental section

### General aspect

Experiments under inert atmosphere were carried out using standard Schlenk techniques under N_2_ atmosphere and SPS-dried deoxygenated solvents. Compound 1a, 2a, 3a, 4, and Zn1 were synthetized according to literature procedures,^25,51,61^ all other compounds were commercially available and used as received. Nuclear magnetic resonance spectra were recorded on a Bruker Avance Neo spectrometer operating at 300.25 MHz for ^1^H NMR spectroscopy. Coupling constants are given in Hertz, atom labeling schemes are detailed in the SI. Elemental analyses were performed at the DCBP Microanalytic Laboratory using a Thermo Scientific Flash 2000 CHNS-O elemental analyzer. High-resolution mass spectrometry was carried out with a Thermo Scientific LTQ Orbitrap XL (ESI-TOF). Cyclic voltammetry and normal pulse voltammetry was recorded using an Autolab PGSTAT101 from Metrohm using 1 mM complex, and 100 mM (Bu_4_N)PF_6_ as supporting electrolyte in 10 mL solvent. Redox potentials were measured using a glassy carbon working electrode, a Ag/AgCl reference electrode (SSCE), and a Pt-wire auxiliary electrode. All potentials were referenced to ferrocene (*E*_1/2_ = 0 V), which was added as an internal standard. UV-vis spectra were recorded on a Shimadzu UV 1800 Spectrophotometer at 298 K using 50 μM complex in MeCN.

### General procedure for the synthesis of complexes 3b–3i

A mixture of [IrCp*Cl_2_]_2_ (1.0 equiv.), ligand (2.05 equiv.), Na_2_CO_3_ (12 equiv.), and NaPF_6_ (20 equiv.) were placed under a nitrogen atmosphere and CH_3_CN (10 mL) was added. The mixture was stirred at the specified temperature for 18 h in the dark. After cooling to room temperature, the mixture was filtered, and the solvent was removed under reduced pressure. The crude solid was extracted with CH_2_Cl_2_ (5 × 50 mL), and the combined organic layers were concentrated *in vacuo* to afford complex 3 as a dark-red solid. The product was recrystallized by slow diffusion of Et_2_O into a solution of the complex in CH_2_Cl_2_.

#### Synthesis of complex 3b

According to the general procedure, reaction of [IrCp*Cl_2_]_2_ (80.0 mg, 0.10 mmol), 2b (80.5 mg, 0.207 mmol), Na_2_CO_3_ (127.7 mg, 1.20 mmol), and NaPF_6_ (337.3 mg, 2.01 mmol) at 50 °C afforded complex 3b as a dark-red solid (142 mg, 0.196 mmol, 97%). Crystals suitable for X-ray diffraction analysis were obtained by slow diffusion of Et_2_O into a CH_2_Cl_2_ solution of 3b. ^1^H NMR (400 MHz, DMSO-d_6_): *δ* 9.69 (d, *J* = 2.0 Hz, 1H, H_6_), 9.12 (d, *J* = 2.0 Hz, 1H, H_4_), 7.11 (dd, *J* = 8.2, 1.3 Hz, 1H, H_6′_), 6.77 (ddd, *J* = 8.2, 7.0, 1.7 Hz, 1H, H_4′_), 6.57 (ddd, *J* = 8.1, 7.0, 1.3 Hz, 1H, H_5′_), 6.53 (dd, *J* = 8.1, 1.7 Hz, 1H, H_3′_), 3.96 (s, 3H, N–CH_3_), 2.04 (s, 3H, C–CH_3_), 1.67 (s, 15H, Cp–CH_3_). ^13^C{^1^H} NMR (101 MHz, DMSO-d_6_): *δ* 163.44 (C_2′_), 160.39 (C_2_), 149.75 (C_4_), 149.05 (C_6_), 143.80 (C_1′_), 135.43 (C_3_), 121.02 (C_5′_), 117.72 (C_4′_), 115.26 (C_6′_), 114.23 (C_5_), 111.62 (C_3′_), 107.72 (C≡N), 86.78 (C_Cp_), 43.19 (N–CH_3_), 16.12 (C–*C*H_3_), 9.04 (Cp–CH_3_). HRMS *m*/*z*: found: 566.1784; calcd for C_24_H_27_IrN_3_O^+^ (M–PF_6_)^+^: 566.17779. Elem. Anal. calcd for C_24_H_27_F_6_IrN_3_OP × CH_2_Cl_2_: C, 37.74; H, 3.67; N, 5.28%. Found: C, 37.93; H, 3.73; N, 5.60%.

#### Synthesis of complex 3c

According to the general procedure, reaction of [IrCp*(Cl)_2_]_2_ (80 mg, 0.105 mmol), 2c (95.2 mg, 0.215 mmol), Na_2_CO_3_ (133.7 mg, 1.26 mmol) and NaPF_6_ (350.01 mg, 2.10 mmol) at 50 °C afforded complex 3c as a dark-orange solid (137 mg, 0.184 mmol, 92%). ^1^H NMR (400 MHz, CD_3_CN): *δ* 9.06 (d, *J* = 2.1 Hz, 1H, H_6_), 8.81 (d, *J* = 2.1 Hz, 1H, H_4_), 7.12 (dd, *J* = 8.2, 1.3 Hz, 1H, H_6′_), 6.82 (ddd, *J* = 8.2, 7.2, 1.4 Hz, 1H, H_5′_), 6.62 (ddd, *J* = 7.8, 7.2, 1.3 Hz, 1H, H_4′_), 6.37 (dd, *J* = 7.8, 1.4 Hz, 1H, H_3′_), 4.01 (s, 3H, N–CH_3_), 2.16 (s, 3H, C–CH_3_), 1.68 (s, 15H, Cp–CH_3_). ^13^C{^1^H} NMR (101 MHz, CD_3_CN): *δ* 164.65 (C_phen_), 163.54 (C_2_), 160.80 (C_5_), 146.63 (C_6_), 144.74 (C_phen_), 144.36 (C_6_), 136.43 (C_3_), 129.84 (CONH_2_), 121.91 (C_5′_), 118.55 (C_4′_), 115.81 (C_6′_), 111.74 (C_3′_), 87.42 (C_Cp_), 43.92 (N–CH_3_), 16.79 (C–*C*H_3_), 9.37 (Cp–CH_3_). HRMS *m*/*z*: found: 584.1894; calcd for C_24_H_29_IrN_3_O_2_^+^ (M–PF_6_)^+^: 584.18835. Elem. Anal. calcd for C_24_H_29_F_6_IrN_3_O_2_P: C, 39.56; H, 4.01; N, 5.77%. Found: C, 40.24; H, 3.60; N, 5.40%.

#### Synthesis of complex 3d

According to the general procedure, reaction of [IrCp*(Cl)_2_]_2_ (80 mg, 0.1 mmol), 2d (83.86 mg, 0.205 mmol), Na_2_CO_3_ (127.71 mg, 1.20 mmol), and NaPF_6_ (337.30 mg, 2.01 mmol) at 50 °C afforded complex 3d as red-brown solid (120 mg, 0.161 mmol, 76.87%). ^1^H NMR (400 MHz, DMSO-d_6_): *δ* = 10.92 (s, 1H, NH), 9.34 (d, *J* = 2.5, 1H, H_6_), 8.44 (d, *J* = 2.5, 1H, H_4_), 7.09 (dd, *J* = 8.0, 1.2, 1H, H_6′_), 6.74 (ddd, *J* = 7.7, 7.6 1.2 Hz, 1H, H_4′_), 6.56 (ddd, *J* = 8.0, 7.6, 1.4, 1H, H_5′_), 6.42 (dd, *J* = 7.7, 1.4, 1H, H_3′_), 4.00 (s, 3H, N–CH_3_), 2.22 (s, 3H, C–CH_3_), 2.05 (s, 3H, NHCO–C*H*_3_), 1.68 (s, 15H, Cp–CH_3_). ^13^C{^1^H} NMR (101 MHz, DMSO) *δ* = 169.67 (N*C*OCH_3_), 163.54 (C_2′_), 152.62 (C_4_), 144.66 (C_1′_), 136.31 (C_3′_), 135.22 (C_2_), 134.67 (C_4_), 133.34 (C_6_), 120.45 (C_4′_), 117.64 (C_5′_), 115.02 (C_6′_), 110.99 (C_3′_), 87.42 (C_Cp_), 43.03 (N–CH_3_), 23.86 (NCO–*C*H_3_), 16.32 (C–*C*H_3_), 9.14 (Cp–*C*H_3_). HRMS *m*/*z*: found: 598.2046; calcd for C_25_H_31_IrN_3_O_2_^+^ (M–PF_6_)^+^: 598.20400. Elem. Anal. calcd for C_25_H_31_F_6_IrN_3_O_2_P: C, 40.43; H, 4.21; N, 5.66%. Found: C, 40.15; H, 4.24; N, 5.50%.

#### Synthesis of complex 3e

According to the general procedure, reaction of [IrCp*(Cl)_2_]_2_ (80.3 mg, 0.105 mmol), 2e (80 mg, 0.210 mmol), Na_2_CO_3_ (67.1 mg, 0.63 mmol) and NaPF_6_ (354.2 mg, 0.211 mmol) at 50 °C afforded complex 3e as a dark red powder (116 mg, 0.163 mmol, 76.85%). ^1^H NMR (400 MHz, CD_3_CN): *δ* 7.83 (d, *J* = 2.7 Hz, 1H, H_6_), 7.70 (d, *J* = 2.7 Hz, 1H, H_4_), 7.07 (dd, *J* = 8.1, 1.3 Hz, 1H, H_6′_), 6.78 (ddd, *J* = 8.1, 7.3, 1.5 Hz, 1H, H_5′_), 6.60 (ddd, *J* = 7.8, 7.3, 1.3 Hz, 1H, H_4′_), 6.35 (dd, *J* = 7.8, 1.5 Hz, 1H, H_3′_), 5.17 (s, 2H, NH_2_), 3.83 (s, 3H, N–CH_3_), 2.02 (s, 3H, C–CH_3_), 1.71 (s, 15H, Cp–CH_3_). ^13^C{^1^H} NMR (101 MHz, CD_3_CN): *δ* 165.10 (C_2′_), 146.42 (C_1′_), 145.58 (C_5_), 136.43 (C_2_), 132.84 (C_4_), 127.68 (C_6_), 121.67 (C_5′_), 118.71 (C_4′_), 115.84 (C_6′_), 111.73 (C_3′_), 87.31 (C_Cp_), 43.60 (N–CH_3_), 17.02 (C–*C*H_3_), 9.84 (Cp–CH_3_). HRMS *m*/*z*: found: 556.1942; calcd for C_23_H_29_IrN_3_O^+^ (M–PF_6_)^+^: 556.19344. Elem. Anal. calcd for C_23_H_29_F_6_IrN_3_OP: C, 39.43; H, 4.17; N, 6.00%. Found: C, 39.25; H, 4.46; N, 5.68%.

#### Synthesis of complex 3f

According to the general procedure, reaction of [IrCp*(Cl)_2_]_2_ (70.0 mg, 0.088 mmol), 2f (71.93 mg, 0.176 mmol), Na_2_CO_3_ (55.9 mg, 0.527 mmol), and NaPF_6_ (295 mg, 1.76 mmol) at room temperature afforded complex 3f as a red solid (115.3 mg, 0.155 mmol, 89%). ^1^H NMR (400 MHz, CD_3_CN): *δ* 8.64 (td, *J* = 6.4, 1.4 Hz, 1H, H_6_), 8.55 (dd, *J* = 7.8, 1.4 Hz, 1H, H_4_), 7.82 (dd, *J* = 7.8, 6.4 Hz, 1H, H_5_), 7.82 (dd, *J* = 9.0, 2.5 Hz, 1H, H_5′_), 7.24 (d, *J* = 2.5 Hz, 1H, H_6′_), 7.15 (d, *J* = 9.0 Hz, 1H, H_3′_), 4.01 (s, 3H, N–CH_3_), 2.16 (s, 3H, C–CH_3_), 1.65 (s, 15H, Cp–CH_3_). ^13^C{^1^H} NMR (101 MHz, CD_3_CN): *δ* 170.87 (C_4′_), 158.08 (C_2_), 149.75 (C_4_), 145.54 (C_2′_), 145.10 (C_6_), 140.03 (C_1′_), 137.02 (C_3_), 125.27 (C_5_), 119.15 (C_5′_), 114.69 (C_3′_), 107.26 (C_6′_), 88.75 (C_Cp_), 44.11 (N–CH_3_), 16.94 (C–*C*H_3_), 9.74 (Cp–CH_3_). HRMS *m*/*z*: found: 586.1684; calcd for C_23_H_27_IrN_3_O_3_^+^ (M–PF_6_)^+^: 586.16762. Elem. Anal. calcd for C_23_H_27_F_6_IrN_3_O_3_P: C, 37.81; H, 3.72; N, 5.75%. Found: C, 37.40; H, 3.70; N, 6.02%.

#### Synthesis of complex 3g

According to the general procedure, reaction of [IrCp*(Cl)_2_]_2_ (70.0 mg, 0.087 mmol), 2g (71.9 mg, 0.175 mmol), Na_2_CO_3_ (55.9 mg, 0.527 mmol) and NaPF_6_ (295 mg, 1.76 mmol) at room temperature afforded complex 3g as a red solid (107.3 mg, 0.139 mmol, 82%). Suitable crystals for X-ray diffraction analysis were obtained by slow diffusion of diethyl ether into a DCM solution of 3g. ^1^H NMR (400 MHz, CD_3_CN) *δ* 8.64 (d, *J* = 6.2 Hz, 1H, H_6_), 8.55 (d, *J* = 7.9 Hz, 1H, H_4_), 7.89 (d, *J* = 2.4 Hz, 1H, H_6′_), 7.83 (dd, *J* = 7.9, 6.2 Hz, 1H, H_5_), 7.59 (dd, *J* = 8.8, 2.4 Hz, 1H, H_4′_), 6.43 (d, *J* = 8.8 Hz, 1H, H_3′_), 3.97 (s, 3H, N–CH_3_), 2.14 (s, 3H, C–CH_3_), 1.65 (s, 15H, Cp–CH_3_). ^13^C{^1^H} NMR (101 MHz, CD_3_CN): *δ* 163.92 (C_1′_), 152.11 (C_5′_), 149.71 (C_4_), 144.90 (C_6_), 142.57 (C_2′_), 136.95 (C_3_), 125.29 (C_5_), 115.41 (C_4′_), 110.33 (C_6′_), 110.30 (C_2_), 88.73 (C_Cp_) 44.10 (N–CH_3_), 16.93 (C–*C*H_3_), 9.70 (Cp-*C*H_3_). HRMS *m*/*z*: found: 586.1683; calcd for C_23_H_27_IrN_3_O_3_^+^ (M–PF_6_)^+^: 586.16762. Elem. Anal. calcd for C_23_H_27_F_6_IrN_3_O_3_P × 0.25 CH_2_Cl_2_: C, 37.14; H, 3.69; N, 5.59%. Found: C, 37.09; H, 3.58; N, 5.40%.

#### Synthesis of complex 3h

According to the general procedure, reaction of [IrCp*(Cl)_2_]_2_ (85.9 mg, 0.106 mmol, 1.0 equiv.), 2h (80.0 mg, 0.211 mmol), Na_2_CO_3_ (67.2 mg, 0.634 mmol) and NaPF_6_ (355.1 mg, 2.11 mmol) at room temperature for 36 h afforded complex 3h as a red solid (143.3 mg, 0.200 mmol, 95%). ^1^H NMR (400 MHz, CD_3_CN): *δ* 8.58 (d, *J* = 6.2 Hz, 1H, H_6_), 8.48 (d, *J* = 7.8 Hz, 1H, H_4_), 7.75 (dd, 7.9, 6.2 Hz, 1H, H_5_), 6.94 (d, *J* = 1.8 Hz, 1H, H_6′_), 6.48 (dd, *J* = 8.0, 1.8 Hz, 1H, H_4′_), 6.22 (d, *J* = 8.0 Hz, 1H, H_3′_), 3.96 (s, 3H, N–CH_3_), 2.29 (s, 3H, C_phen_–CH_3_), 2.12 (s, 3H, C_pyr_–CH_3_), 1.66 (s, 15H, Cp–CH_3_). ^13^C{^1^H} NMR (101 MHz, CD_3_CN): *δ* 165.12 (C_phen_), 159.70 (C_2_), 148.93 (C_4_), 144.21 (C_phen_), 143.10 (C_6_), 136.98 (C_3_), 131.99 (C_5′_), 124.49 (C_5_), 111.35 (C_3′_), 87.37 (C_Cp_), 43.72 (N-CH_3_), 20.67 (C_phen_–*C*H_3_), 16.89 (C_pyr_–*C*H_3_), 9.69 (Cp–*C*H_3_). HRMS *m*/*z*: found: 555.1992; calcd for C_24_H_30_IrN_2_O^+^ (M–PF_6_)^+^: 555.19819. Elem. Anal. calcd for C_24_H_30_F_6_IrN_2_OP: C, 41.20; H, 4.32; N, 4.00%. Found: C, 41.34; H, 4.31; N, 4.11%.

#### Synthesis of complex 3i

According to the general procedure, reaction [IrCp*(Cl)_2_]_2_ (82.4 mg, 0.101 mmol), 2i (80.0 mg, 0.203 mmol), Na_2_CO_3_ (64.5 mg, 0.609 mmol) and NaPF_6_ (340.7 mg, 2.04 mmol) at room temperature for 36 h afforded complex 3i as a red solid (145.4 mg, 0.198 mmol, 98%). ^1^H NMR (400 MHz, CD_3_CN): *δ* 8.59 (d, *J* = 6.2 Hz, 1H, H_6_), 8.49 (d, *J* = 7.8 Hz, 1H, H_4_), 7.76 (dd, *J* = 7.8, 6.2 Hz, 1H, H_5_), 7.00 (d, *J* = 8.8 Hz, 1H, H_6′_), 6.51 (dd, *J* = 8.8, 2.7 Hz, 1H, H_5′_), 5.94 (d, *J* = 2.7 Hz, 1H, H_3′_), 3.98 (s, 1H, N–CH_3_), 3.58 (s, 1H, O–CH_3_), 2.14 (s, 1H, C–CH_3_), 1.65 (s, 15H, Cp–CH_3_). ^13^C{^1^H} NMR (101 MHz, CD_3_CN): *δ* 159.68 (C_phen_), 159.35 (C_2_), 154.31 (C_4′_), 149.08 (C_4_), 145.46 (C_1′_), 144.37 (C_6_), 136.95 (C_3_), 124.58 (C_5_), 115.13 (C_6′_), 107.09 (C_5′_), 97.47 (C_3′_), 87.37 (C_Cp_) 56.48 (O–CH_3_), 43.84 (N–CH_3_), 16.99 (C–*C*H_3_), 9.70 (Cp–*C*H_3_). HRMS *m*/*z*: found: 571.194; calcd for C_24_H_30_IrN_2_O_2_^+^ (M–PF_6_)^+^: 571.1931. Elem. Anal. calcd for C_24_H_30_F_6_IrN_2_O_2_P × 0.5 CH_2_Cl_2_: C, 38.81; H, 4.12; N, 3.69%. Found: C, 38.76; H, 4.03; N, 3.81%.

### DFT calculations

All ground-state structures were optimised with ORCA 6.0^38^ using the B3LYP^33,34^ functional augmented by the Grimme D3(BJ)^35,36^ dispersion correction. A def2-TZVP^37^ basis set was employed for every element together with the matching def2/J auxiliary basis; coulomb and exact-exchange integrals were evaluated with the RIJCOSX^62^ approximation. Implicit solvation by DMSO (*ε* = 46.8) was included throughout *via* the CPCM model.^38–40^ Tight SCF convergence criteria were applied (energy change <10^−8^ Eh; RMS density change <10^−7^) and a maximum of 500 SCF iterations was allowed. Optimisations used analytic gradients and were deemed converged when the largest Cartesian gradient component dropped below 3 × 10^−4^ Eh a_0_^−1^. Each stationary point was confirmed as a true minimum by an analytic frequency calculation at the same level of theory (no imaginary frequencies). Zero-point energies and 298 K thermal corrections reported in the main text derive from these frequency runs. All jobs ran on 16 CPU cores (%pal nprocs 16 end) with 8 GB RAM per core (%maxcore 8000). Single-point calculations on the optimised geometries were carried out at the same B3LYP^33,34^-D3(BJ)^35,36^/def2-TZVP^37^/CPCM(DMSO)^38–40^ level, but with extended print options to obtain detailed electronic information (MOs, overlap matrix, Löwdin^55^ and fragment charges, Wiberg/Loewdin bond orders, orbital populations). The Loewdin bond-order threshold was set to 0.05. Vertical singlet excitation energies were obtained by time-dependent DFT^38^ at the same level of theory. Thirty lowest-energy roots were computed (NROOTS 30). Solvent, basis set, dispersion, RIJCOSX^62^ and SCF settings were identical to those used in the SCF step.

### Hydride formation

In an NMR tube, the desired complex 3a–3i (0.02 mmol) and HCOOLi·H_2_O (0.20 mmol) were dissolved in DMSO-d_6_ (0.5 mL) and HCOOLi·H_2_O (0.20 mmol). ^1^H-NMR spectra was recorded after 1 hour and an additional ^1^H-NMR and ^1^H–^1^H NOEY spectra were recorded after 6 hours.

### Hydride stability

In an NMR tube, the desired complex (0.02 mmol) was dissolved in DMSO-d_6_ (0.30 mL). An aqueous solution of HCOOLi·H_2_O (0.10 mL of a 20 mM solution, 0.02 mmol) was added, and the reaction mixture was kept at room temperature and monitored by ^1^H NMR spectroscopy until full conversion (*ca.* 1 h), as indicated by the exclusive presence of the reduced form of the complex. Upon full conversion, benzoic acid (0.20 mL of a 0.30 M solution in DMSO-d_6_, 0.06 mmol) was added. The reaction was monitored over time by ^1^H NMR spectroscopy.

### Crystal-structure determination

Suitable single crystals of complexes 3b, 3g, and 3i immersed in parabar oil were mounted at ambient conditions and transferred into a stream of cold nitrogen (173 K). All measurements were carried out on a RIGAKU XtaLAB Synergy R, HyPix-Arc100 area-detector diffractometer^63^ using mirror optics monochromated Mo Kα radiation (*λ* = 0.71073 Å). The unit cell constants and an orientation matrix for data collection were obtained from a least-squares refinement of the setting angles of reflections in the range 2.10° < *θ* < 33.51°. Frames were collected using ω scans, with 1.6 seconds exposure time, a rotation angle of 0.5° per frame, a crystal-detector distance of 43.0 mm, at *T* = 173.0(1) K. Data reduction was performed using the CrysAlisPro^63^ program. The intensities were corrected for Lorentz and polarization effects, and a numerical absorption correction based on Gaussian integration over a multifaceted crystal model with additional empirical absorption correction using spherical harmonics using SCALE3 ABSPACK in CrysAlisPr^63^ was applied. Data collection and refinement parameters are given in Tables S2–S4. The structures were solved by intrinsic phasing using SHELXT,^64^ which revealed the positions of all non-hydrogen atoms. All non-hydrogen atoms were refined anisotropically. H-atoms were assigned in geometrically calculated positions and refined using a riding model where each H-atom was assigned a fixed isotropic displacement parameter with a value equal to 1.2U_eq_ of its parent atom (1.5U_eq_ for methyl groups). The structure of 3g was refined as an inversion twin. Dynamic disorder of the PF_6_ anions was treated with two disorder components for each of the two symmetry-independent anions. Refinement of the structures was carried out on *F*^2^ using full-matrix least-squares procedures, which minimized the function ∑w(*F*_o_^2^ – *F*_c_^2^)^2^. The weighting scheme was based on counting statistics and included a factor to downweight the intense reflections. All refinements were performed using the SHELXL-2014/7^65^ program in OLEX2.^66^ Data collection and refinement parameters for all compounds are given in Tables S2–S4. Crystallographic data for all structures have been deposited with the Cambridge Crystallographic Data entre (CCDC) as supplementary publication number 2555905 (3b), 2555904 (3g), 2555906 (3i).

## Conflicts of interest

There are no conflicts to declare.

## Supplementary Material

DT-055-D6DT01322H-s001

DT-055-D6DT01322H-s002

## Data Availability

The data supporting this article have been included as part of the supplementary information (SI). Supplementary information: all relevant data, including synthetic and catalytic procedures, analytical data for all new compounds, DFT calculation setup, data for hydride preparation and stability assessment as well as crystallographic details. See DOI: https://doi.org/10.1039/d6dt01322h. CCDC 2555904 (3g), 2555905 (3b) and 2555906 (3i) contain the supplementary crystallographic data for this paper.^67*a*–*c*^

## References

[cit1] Wang D., Astruc D. (2015). Chem. Rev..

[cit2] Halpern J. (1981). Inorg. Chim. Acta.

[cit3] Halpern J. (1982). Science.

[cit4] Clapham S. E., Hadzovic A., Morris R. H. (2004). Coord. Chem. Rev..

[cit5] Wiedner E. S., Chambers M. B., Pitman C. L., Bullock R. M., Miller A. J. M., Appel A. M. (2016). Chem. Rev..

[cit6] Hartwig J. F. , Organotransition Metal Chemistry : From Bonding to Catalysis, University Science Books, 2010

[cit7] Hall M., Bommarius A. S. (2011). Chem. Rev..

[cit8] Paul C. E., Hollmann F. (2016). Appl. Microbiol. Biotechnol..

[cit9] Sellés Vidal L., Kelly C. L., Mordaka P. M., Heap J. T. (2018). Biochim. Biophys. Acta, Proteins Proteomics.

[cit10] Polyansky D. E., Cabelli D., Muckerman J. T., Fukushima T., Tanaka K., Fujita E. (2008). Inorg. Chem..

[cit11] Kobayashi K., Koizumi T. A., Ghosh D., Kajiwara T., Kitagawa S., Tanaka K. (2018). Dalton Trans..

[cit12] Oyama D., Ito K., Ukawa N., Morihara A., Takase T. (2025). Organometallics.

[cit13] Stout D. M., Meyers A. I. (1982). Chem. Rev..

[cit14] Hantzsch A. (1881). Ber. Dtsch. Chem. Ges..

[cit15] McSkimming A., Bhadbhade M. M., Colbran S. B. (2013). Angew. Chem., Int. Ed..

[cit16] McSkimming A., Chan B., Bhadbhade M. M., Ball G. E., Colbran S. B. (2015). Chem. – Eur. J..

[cit17] McLoughlin E. A., Waldie K. M., Ramakrishnan S., Waymouth R. M. (2018). J. Am. Chem. Soc..

[cit18] Charette B. J., Ziller J. W., Heyduk A. F. (2021). Inorg. Chem..

[cit19] Bruch Q. J., Tanushi A., Müller P., Radosevich A. T. (2022). J. Am. Chem. Soc..

[cit20] Race J. J., Albrecht M. (2023). ACS Catal..

[cit21] Leigh V., Carleton D. J., Olguin J., Mueller-Bunz H., Wright L. J., Albrecht M. (2014). Inorg. Chem..

[cit22] Boyd P. D. W., Wright L. J., Zafar M. N. (2011). Inorg. Chem..

[cit23] Doster M. E., Johnson S. A. (2009). Angew. Chem., Int. Ed..

[cit24] Shi Q., Thatcher R. J., Slattery J., Sauari P. S., Whitwood A. C., McGowan P. C., Douthwaite R. E. (2009). Chem. – Eur. J..

[cit25] Lentz N., Reuge S., Albrecht M. (2023). ACS Catal..

[cit26] Nagashima H., Kondo H., Hayashida T., Yamaguchi Y., Gondo M., Masuda S., Miyazaki K., Matsubara K., Kirchner K. (2003). Coord. Chem. Rev..

[cit27] Kindervater M. B., Staroverov V. N., Jackman K. M. K., Fogh A. A., Kelley L. S. G., Lim L., Sirohey S. A., Boyle P. D., Blacquiere J. M. (2023). Dalton Trans..

[cit28] Jiménez-Tenorio M., Puerta M. C., Valerga P. (2004). Eur. J. Inorg. Chem..

[cit29] Gottschalk-Gaudig T., Folting K., Caulton K. G. (1999). Inorg. Chem..

[cit30] Johnson T. J., Folting K., Streib W. E., Martin J. D., Huffman J. C., Jackson S. A., Eisenstein O., Caulton K. G. (1995). Inorg. Chem..

[cit31] Yan C., Chang T., Chen Y.-S., Paterson A. L., McElheny D., Mankad N. P. (2026). Chem. Sci..

[cit32] Orpen A. G., Brammer L., Allen F. H., Kennard O., Watson D. G., Taylor R. (1989). J. Chem. Soc., Dalton Trans..

[cit33] Lee C., Yang W., Parr R. G. (1988). Phys. Rev. B: Condens. Matter Mater. Phys..

[cit34] Becke A. D. (1993). J. Chem. Phys..

[cit35] Grimme S., Antony J., Ehrlich S., Krieg H. (2010). J. Chem. Phys..

[cit36] Grimme S., Ehrlich S., Goerigk L. (2011). J. Comput. Chem..

[cit37] Weigend F., Ahlrichs R. (2005). Phys. Chem. Chem. Phys..

[cit38] Neese F. (2025). Wiley Interdiscip. Rev.: Comput. Mol. Sci..

[cit39] Takano Y., Houk K. N. (2005). J. Chem. Theory Comput..

[cit40] Barone V., Cossi M. (1998). J. Phys. Chem. A.

[cit41] van der Vlugt J. I. (2012). Eur. J. Inorg. Chem..

[cit42] Chirik P. J. (2011). Inorg. Chem..

[cit43] Lyaskovskyy V., de Bruin B. (2012). ACS Catal..

[cit44] Grützmacher H. (2008). Angew. Chem., Int. Ed..

[cit45] Kaim W., Schwederski B. (2010). Coord. Chem. Rev..

[cit46] Complex 4 is comprised of PYA ligands without a methyl group at the pyridine C3 position, since the tetrahedral geometry is not compatible with two ligands derived from 2a. The iridium complex with the exact same ligand as in complex 4 was synthesized following reported procedures (ref. 51) and shows electrochemical behavior very similar to that of 3a, including two reversible one-electron processes at +1.05 V and +1.32 V (Fig. S3c). The 0.15 V shift of all oxidations to higher potential suggests stronger donor properties of the C3-methylated ligand in 3a compared to the C3-protonated system, though the generally similar features indicate no fundamental difference in redox behavior and demonstrate complex 4 as a useful model for identifying ligand-centered processes with these PYA complexes

[cit47] Storr T., Verma P., Shimazaki Y., Wasinger E. C., Stack T. D. P. (2010). Chem. – Eur. J..

[cit48] Pierpont C. G. (2011). Inorg. Chem..

[cit49] Chaudhuri P., Wieghardt K., Weyhermüller T., Paine T. K., Mukherjee S., Mukherjee C. (2005). Biol. Chem..

[cit50] Kaim W. (1987). Coord. Chem. Rev..

[cit51] Lentz N., Reuge S., Beaufils A., Albrecht M. (2024). Organometallics.

[cit52] Trotta C., Menendez Rodriguez G., Zuccaccia C., Macchioni A. (2024). ACS Catal..

[cit53] Saba T., Li J., Burnett J. W. H., Howe R. F., Kechagiopoulos P. N., Wang X. (2021). ACS Catal..

[cit54] Aamer E., Thöming J., Baune M., Reimer N., Dringen R., Romero M., Bösing I. (2022). Sci. Rep..

[cit55] Bruhn G., Davidson E. R., Mayer I., Clark A. E. (2006). Int. J. Quantum Chem..

[cit56] Asundi A. S., Lui K. H., Dressel J. M., Marron D. P., Sokaras D., Waymouth R. M., Sarangi R. (2025). Inorg. Chem..

[cit57] Stroscio G. D., Goldman N. (2025). J. Phys. Chem. A.

[cit58] Zámbó G. G., Esslinger C. A., Sauer M. J., Rüter I., Reich R. M., Demeshko S., Meyer F., Kühn F. E. (2024). Catal. Sci. Technol..

[cit59] Xu Y., Celik M. A., Thompson A. L., Cai H., Yurtsever M., Odell B., Green J. C., Mingos D. M. P., Brown J. M. (2009). Angew. Chem., Int. Ed..

[cit60] Wang H., Pignolet L. H. (1980). Inorg. Chem..

[cit61] Lentz N., Albrecht M. (2022). ACS Catal..

[cit62] Kossmann S., Neese F. (2010). J. Chem. Theory Comput..

[cit63] CrysAlis PRO, Version 1.171.40.37a, Oxford Diffraction Ltd, Yarnton, UK, 2018

[cit64] Sheldrick G. M. (2015). Acta Crystallogr., Sect. A: Found. Adv..

[cit65] Sheldrick G. M. (2015). Acta Crystallogr., Sect. C:Struct. Chem..

[cit66] Dolomanov O. V., Bourhis L. J., Gildea R. J., Howard J. A. K., Puschmann H. (2009). J. Appl. Crystallogr..

[cit67] (a) CCDC 2555904: Experimental Crystal Structure Determination, 2026, 10.5517/ccdc.csd.cc2rsmjq

