# Supplementary material for: Pyridinium amidate (PYA) substituents impact ligand-centered hydride formation and (catalytic) hydride transfer reactivity
Source: Dalton Trans. 2026 Jun 29;55(29):10885–98. doi: 10.1039/d6dt01322h (PMC13347384; doi:10.1039/d6dt01322h)
Supplement: DT-055-D6DT01322H-s001 [file DT-055-D6DT01322H-s001.pdf]

Supplementary Information (SI) for Dalton Transactions.
This journal is © The Royal Society of Chemistry 2026

Electronic Supporting Information

belonging to:

Pyridinium amidate (PYA) substituents impact ligand-centered hydride formation and (catalytic) hydride transfer reactivity

Laura Monte, Nicolas Lentz, Martin Albrecht

Department of Chemistry, Biochemistry, and Pharmaceutical Sciences, University of Bern, Freiestrasse 3, 3012 Bern, Switzerland

E-mail: martin.albrecht@unibe.ch

CONTENTS

1 General Aspects S2
2 Synthetic Procedure S3
2.1 Synthesis of pyridinium salts **1b–1e** S3
2.2 Synthesis of pyridinium aminophenoles **2b–2i** S5
2.3 Synthesis of complexes **3b–3i** S9
3 Electrochemical Analysis S15
4 DFT Calculations S16
4.1 Geometry optimization set up..... S16
4.2 SCF and population analysis set up S16
4.3 TD-DFT calculation set up S17
5 Hydride preparation S20
5.1 Spectra of *para*- and *ortho*-hydrides S20
6 Hydride stability..... S31
7 Catalytic Data..... S43
8 NMR spectra of new compounds S45
9 Crystallography..... S67
10 References S70

1 GENERAL ASPECTS

All reactions were performed under air unless stated otherwise. Experiments under inert atmosphere were carried out using standard Schlenk techniques under N_2 atmosphere and dry deoxygenated solvents. Dry solvents were taken from a solvent purification system (SPS). Compound **1a**, **2a**, **3a, 4**, and **Zn1** were synthetized according to literature procedures.[S1–S3] All other compounds were commercially available and used as received.

Nuclear magnetic resonance spectra were recorded on a Bruker Avance Neo spectrometer operating at 300.25 MHz for ^{1}H spectroscopy. Coupling constants are given in Hertz. The following abbreviations are used: s, singlet; d, doublet; t, triplet; m, multiplet. Elemental analyses were performed at the DCBP Microanalytic Laboratory using a Thermo Scientific Flash 2000 CHNS-O elemental analyzer. High-resolution mass spectrometry was carried out with a Thermo Scientific LTQ Orbitrap XL (ESI-TOF).

UV-vis spectra were recorded on a Shimadzu UV 1800 Spectrophotometer, with a silicon photodiode detector ranging from 190 to 1100 nm. Starna Scientific quartz cuvettes (type 23-N/Q/10) with a path length of 10 mm were used. The spectra were collected at 298 K. For each measured complex a 10 mM solution in MeCN was made and diluted to 0.05 mM.

Cyclic voltammetry and normal pulse voltammetry was recorded using an Autolab PGSTAT101 from Metrohm using 1 mM complex, and 100 mM $(Bu_4N)PF_6$ as supporting electrolyte in 10 mL solvent. Redox potentials were measured using a glassy carbon working electrode, a Ag/AgCl reference electrode (SSCE), and a Pt-wire auxiliary electrode. All potentials were tabulated vs ferrocene ($E_{1/2}$ = 0 V), which was added as an internal standard.

2 SYNTHETIC PROCEDURE

2.1 Synthesis of pyridinium salts 1b–1e

2-chloro-5-cyano-1,3-dimethylpyridin-1-ium triflate (1b)

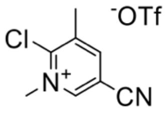

Under a nitrogen atmosphere, 2-chloro-3-methyl-5-cyanopyridine (1.00 g, 6.00 mmol, 1.0) and methyl trifluoromethanesulfonate (0.710 mL, 6.00 mmol) were dissolved in dry CH_2Cl_2 (15 mL). The reaction mixture was stirred at room temperature for 7 h, after which the solvent was removed *in vacuo*. The resulting crude solid was washed with Et_2O (3 × 30 mL) to afford **1b** as a white solid (1.87 g, 99%).

^{1}H NMR (400 MHz, CD_3CN): δ 9.12 (d, J = 2.0 Hz, 1H, H_6), 8.70 (d, J = 2.0 Hz, 1H, H_4), 4.34 (s, 3H, N–CH_3), 2.59 (s, 3H, C–CH_3).

^{13}C{^{1}H} NMR (101 MHz, CD_3CN): δ 150.20 (C_4), 149.93 (C_6), 141.86 (C_3), 113.48 (C_{pyr}), 111.64 (C_{pyr}), 50.14 (N–CH_3), 20.24 (C–CH_3).

HRMS m/z found: 167.0370; calcd for $C_8H_8ClN_2^+$ (M–OTf)$^+$: 167.03705.

5-carbamoyl-2-chloro-1,3-dimethylpyridin-1-ium triflate (1c)

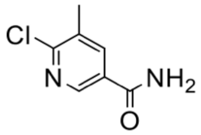

In a round-bottom flask, 6-chloro-5-methylnicotinonitrile (500 mg, 3.28 mmol) was dissolved in a mixture of MeOH/H_2O (1:1 v/v, 10 mL). KOH (367 mg, 6.55 mmol), pre-dissolved in water (0.365 mL), was added and the reaction mixture was stirred at 50 °C for 1.5 h. The reaction mixture was then cooled to 0 °C until a white precipitate formed. The solid was collected by filtration, washed with Et_2O (3 × 40 mL), extracted with CH_2Cl_2 (2 × 20 mL), and dried under vacuum to afford the product as a white powder (289 mg, 1.69 mmol, 52%).

^{1}H NMR (400 MHz, $(CD_3)_2SO$): δ 8.68 (d, *J* = 2.4 Hz, 1H, H_6), 8.21–8.19 (m, 1H, H_4), 8.15 (s, 1H, $CONH_2$), 7.64 (s, 1H, $CONH_2$), 2.38 (s, 3H, C-CH_3)

^{13}C{^{1}H} NMR (101 MHz, $(CD_3)_2SO$): δ 165.44 ($CONH_2$), 152.74 (C_2), 146.30 (C_6), 139.01 (C_4), 131.90 (C_3), 129.29 (C_5), 19.00 (C-*C*H_3)

HRMS m/z found: 171.0316; calcd. for $C_7H_7ClN_2O$ $(M)^+$: 171.03197.

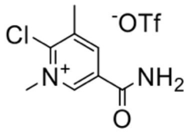

6-chloro-5-methylnicotinamide (1.00 g, 5.86 mmol) was dissolved in CH_2Cl_2 (15 mL). Methyl trifluoromethanesulfonate (0.664 mL, 5.86 mmol) was added under a nitrogen atmosphere, and the reaction mixture was stirred at room temperature for 6 h. All volatiles were then removed *in vacuo*, and the resulting crude solid was washed with Et_2O (3 × 40 mL) to afford **1c** as a white solid (1.29 g, 3.87 mmol, 66%).

^{1}H NMR (400 MHz, CD$_3$CN): δ 9.12–9.04 (m, 1H, H$_6$), 8.76 – 8.70 (m, 1H, H$_4$), 7.33 (br, 1H, CO–NH$_2$), 6.61 (br, 1H, CO–NH$_2$), 4.35 (s, 3H, N–CH$_3$), 2.61 (s, 3H, C–C*H$_3$*).

^{13}C{^{1}H} NMR (101 MHz, CD$_3$CN): δ 163.30 (C$_{pyr}$), 150.58 (C$_{pyr}$), 146.52 (C$_4$), 146.29 (C$_6$), 140.52 (C$_3$), 132.05 (CONH$_2$), 49.66 (N–CH$_3$), 20.20 (C–*C*H$_3$).

HRMS m/z found: 185.0475; calcd. for $C_8H_{10}ClN_2O^+$ (M–OTf)$^+$: 185.04762.

5-acetamido-2-chloro-1,3-dimethylpyridin-1-ium triflate (1d)

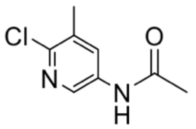

In a round-bottom flask, 6-chloro-5-methylpyridin-3-amine (1.00 g, 7.01 mmol) and NEt$_3$ (1.47 mL, 10.5 mmol) were dissolved in CH$_2$Cl$_2$ (15 mL). The reaction mixture was cooled to 0 °C, and acetyl chloride (0.60 mL, 8.42 mmol) was added dropwise under a nitrogen atmosphere. The mixture was then allowed to warm to room temperature and stirred for 18 h. The resulting suspension was filtered, and the solvent was removed under reduced pressure. The residue was extracted with THF (3 × 50 mL), and all volatiles were evaporated to afford the title compound as a white solid (1.12 g, 5.96 mmol, 85%).

^{1}H NMR (400 MHz, (CD$_3$)$_2$SO): δ 10.24 (s, 1H, NH), 8.40 (d, J = 2.7 Hz, 1H, H$_6$), 8.01 (d, J = 2.7 Hz, 1H, H$_4$), 2.30 (s, 3H, C$_{pyr}$ –CH$_3$), 2.07 (s, 3H, NHCOC*H$_3$*).

^{13}C{^{1}H} NMR (101 MHz, (CD$_3$)$_2$SO): δ 168.90 (NH*CO*CH$_3$), 143.60 (C$_5$), 137.61 (C$_6$), 135.52 (C$_3$), 131.83 (C$_2$), 129.72 (C$_4$), 23.80 (NHCO*CH$_3$*), 19.21 (C$_{pyr}$–*CH$_3$*).

HRMS m/z found: 185.0475; calcd. for $C_8H_{10}ClN_2O^+$ (M)$^+$: 185.04762.

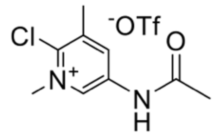

N-(6-chloro-5-methylpyridin-3-yl)acetamide (1.00 g, 5.42 mmol) was dissolved in CH$_2$Cl$_2$ (15 mL). Methyl trifluoromethanesulfonate (0.593 mL, 5.42 mmol) was added under a nitrogen atmosphere, and the reaction mixture was stirred at room temperature for 3 h. All volatiles were then removed *in vacuo*, and the resulting crude solid was washed with Et$_2$O (3 × 40 mL) to afford **1d** as an off-white solid (1.45 g, 4.16 mmol, 77%).

^{1}H NMR (400 MHz, CD$_3$CN): δ 9.41 (s, 1H, NH), 9.32 (d, J = 2.5 Hz, 1H, H$_6$), 8.30 (d, J = 2.5 Hz, 1H, H$_4$), 4.27 (s, 3H, N–CH$_3$), 2.52 (s, 3H, C–CH$_3$), 2.17 (s, 3H, NHCO–C*H$_3$*).

^{13}C{^{1}H} NMR (101 MHz, CD$_3$CN): δ 171.08 (NH*C*OCH$_3$), 140.15 (C$_{pyr}$), 138.13 (C$_{pyr}$), 136.66 (C$_6$), 134.47 (C$_4$), 48.17 (N–CH$_3$), 22.38 (NHCO–*C*H$_3$), 18.52 (C–*C*H$_3$).

HRMS m/z found: 199.0633; calcd. for $C_9H_{12}ClN_2O^+$ (M–OTf)$^+$: 199.0632.

5-amino-2-chloro-1,3-dimethylpyridin-1-ium triflate (1e)

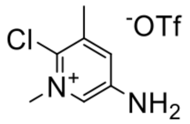

6-Chloro-5-methylpyridin-3-amine (1.00 g, 7.00 mmol) and methyl trifluoromethanesulfonate (0.770 mL, 7.00 mmol) were dissolved in dry CH_2Cl_2 (15 mL). The reaction mixture was stirred at room temperature for 2 h, after which the solvent was concentrated *in vacuo*. The resulting crude solid was washed with Et_2O (3 × 50 mL) to afford **1e** as a light-pink solid (1.94 g, 90%).

^{1}H NMR (400 MHz, $(CD_3)_2SO$): 8.08 (d, J = 2.8 Hz, 1H, H_6), 7.54 (d, J = 2.8 Hz, 1H, H_4), 4.19 (s, 3H, N-CH_3), 2.37 (s, 3H, C-CH_3)

^{13}C{^{1}H} NMR (101 MHz, $(CD_3)_2SO$): δ 146.12 (C_3), 137.30 (C_2), 131.07 (C_5), 129.92 (C_6), 129.34 (C_4), 48.01 (N-CH$_3$), 19.39 (C-CH$_3$)

HRMS m/z found: 157,0527; calcd. for $C_7H_{10}ClN_2$+ (M–OTf)$^+$: 157,05270.

2.2 Synthesis of pyridinium aminophenoles 2b–2i

Cyanopyridinium aminophenol 2b

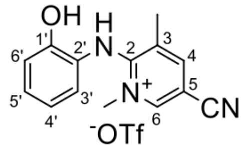

In a microwave vial, **1b** (525 mg, 1.57 mmol), 1,2-aminophenol (178.5 mg, 1.57 mmol), and $NaHCO_3$ (139 mg, 1.57 mmol) were dissolved in MeCN (10 mL). The reaction mixture was stirred at 80 °C for 18 h. It was then allowed to cool to room temperature, and the solvent was removed *in vacuo*. The residue was extracted with CH_2Cl_2 (3 × 30 mL). The combined organic layers were concentrated under reduced pressure, and the resulting oil was dried under high vacuum for 18 h to afford **2b** as a yellow solid (570 mg, 1.46 mmol, 93%).

^{1}H NMR (400 MHz, CD_3CN): δ 7.91 (d, J = 2.2 Hz, 1H, H_6), 6.91–6.89 (m, 1H, H_4), 6.83 (ddd, J = 8.0, 7.3, 1.6 Hz, 1H, $H_{5'}$), 6.74 (dd, J = 8.0, 1.5 Hz, 1H, $H_{6'}$), 6.71 (ddd, J = 7.6, 7.3, 1.5 Hz, 1H, $H_{4'}$), 6.60 (dd, J = 7.6, 1.6 Hz, 1H, $H_{3'}$), 6.15 (br, 1H, NH) 3.52 (s, 3H, N–CH_3), 1.63–1.57 (m, 3H, C–CH_3).

^{13}C{^{1}H} NMR (101 MHz, CD_3CN): δ 151.14 (C_2), 148.97 (C_{phen}), 146.69 (C_6), 138.53 (C_{phen}), 135.52 (C_4), 127.38 (C_2), 123.46 ($C_{5'}$), 121.84 ($C_{3'}$), 119.88 ($C_{4'}$), 118.3 (C_5), 114.70 ($C_{6'}$), 87.91 (CN), 42.15 (N–CH_3), 21.10 (C–CH$_3$)

HRMS m/z found: 240,1130; calcd. for $C_{14}H_{14}ON_3$ (M–OTf)$^+$: 240.11314.

Carbamoyl-pyridinium aminophenole 2c

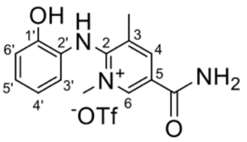

In a microwave vial, **1c** (500 mg, 1.49 mmol), 1,2-aminophenol (163 mg, 1.49 mmol), and $NaHCO_3$ (150.6 mg, 1.79 mmol) were dissolved in MeCN (10 mL). The reaction mixture was stirred at 80 °C for 18 h. It was then allowed to cool to room temperature, and the solvent was removed *in vacuo*. The residue was extracted with CH_2Cl_2 (3 × 30 mL). The combined organic layers were concentrated under reduced pressure, and the resulting oil was dried under high vacuum for 18 h to afford the title compound **2c** as a yellow solid (407,4 mg, 1.39 mmol, 93%).

^{1}H NMR (400 MHz, CD_3CN): δ 8.36 (d, J = 2.1 Hz, 1H, H_4), 7.98 (dd, J = 2.1, 1.1 Hz, 1H, H_6), 7.14 (ddd, J = 8.2, 7.3, 1.7 Hz, 1H, $H_{5'}$), 7.04 (dd, J = 7.8, 1.7 Hz, 1H, $H_{3'}$), 6.93 (dd, J = 8.2, 1.4 Hz, 1H, $H_{6'}$), 6.90 (ddd, J = 7.8, 7.3, 1.4 Hz, 1H, $H_{4'}$), 3.81 (s, 3H, N–CH_3), 1.96 (s, 3H, C–CH_3).

^{13}C{^{1}H} NMR (101 MHz, CD_3CN): δ 165.17 ($CONH_2$), 154.10 (C_2), 151.61 (C_{phenol}), 142.24 (C_6), 141.89 (C_4), 128.99 (C_{phenol}), 128.23 ($C_{5'}$), 127.82 (C_3), 126.25 ($C_{3'}$), 121.19 ($C_{4'}$), 120.81 (C_5), 116.53 ($C_{6'}$), 44.88 (N–CH_3), 19.73F (C–*C*H_3).

HRMS m/z found: 258,1235; calcd. for $C_{14}H_{16}N_30_2^+$ (M–OTf)$^+$: 258,12370.

Acetamido-pyridinium aminophenole 2d

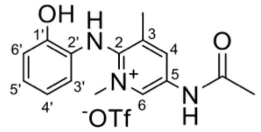

In a microwave vial, **1d** (500 mg, 1.43 mmol), 1,2-aminophenol (156.5 mg, 1.43 mmol), and $NaHCO_3$ (361.3 mg, 4.3 mmol) were dissolved in MeCN (10 mL). The reaction mixture was stirred at 80 °C for 18 h. It was then allowed to cool to room temperature, and the solvent was removed *in vacuo*. The residue was extracted with CH_2Cl_2 (3 × 30 mL). The mixture was concentrated under reduced pressure, and the resulting oil was dried under high vacuum for 18 h to afford the title compound **2d** as a yellow solid (555 mg, 1.32 mmol, 88%).

^{1}H NMR (400 MHz, CD_3CN): δ 8.67 (d, J = 2.4 Hz, 1H, H_6), 7.77 (d, J = 2.4 Hz, 1H, H_4), 7.01 (ddd, J = 8.1, 7.2, 1.7 Hz, 1H, H_{phen}), 6.87 (dd, J = 7.9, 1.6 Hz, 2H, H_{phen}), 6.80 (td, J = 7.5, 1.4 Hz, 1H, H_{phen}), 3.81 (s, 3H, N–CH_3), 2.10 (s, 3H, NCO–CH_3), 2.01 (s, 3H, C–CH_3).

^{13}C{^{1}H} NMR (101 MHz, CD_3CN): δ 170.50 ($NHCOCH_3$), 151.09 (C_{phen}), 149.46 (C_1), 137.17 (C_4), 131.35 (C_{pyr}), 131.29 (C_{pyr}), 130.88 (C_6), 129.41 (C_{phen}), 126.72 (C_{phen}), 123.67 (C_{phen}), 120.68 (C_{phen}), 116.84 (C_{phen}), 45.35 (N–CH_3), 23.90 (NCO–*C*H_3), 19.16 (C–*C*H_3).

HRMS m/z found: 272,13935; calcd. for $C_{15}H_{18}N_30_2^+$ (M–OTf)$^+$: 272.1392**.**

Amino-pyridinium aminophenole 2e

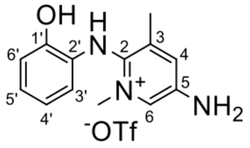

In a microwave vial, **1e** (150 mg, 0.49 mmol), 1,2-aminophenol (53.4 mg, 0.49 mmol), and Na_2CO_3 (81.2 mg, 0.98 mmol) were dissolved in MeCN (4 mL). The reaction mixture was stirred at 100 °C for 18 h. It was then allowed to cool to room temperature, and the solvent was removed *in vacuo*. The residue was triturated/extracted with CH_2Cl_2 (3 × 30 mL). The combined organic layers were concentrated under reduced pressure, and the resulting oil was dried under high vacuum for 18 h to afford the title compound as a yellow/brown solid (135 mg, 0.36 mmol, 73%).

^{1}H NMR (400 MHz, CD_3CN) δ 7.70 (d, J = 2.7 Hz, 1H, H_6), 7.49 (d, J = 2.7 Hz, 1H, H_4), 6.90 (dd, J = 8.0, 1.5 Hz, 1H, $H_{6'}$), 6.82 (ddd, J = 8.0, 7.7, 1.6 Hz, 1H, $H_{5'}$), 6.69 (ddd, J = 7.7, 7.6, 1.5 Hz, 1H, $H_{4'}$), 6.40 (dd, J = 7.6, 1.6 Hz, 1H, $H_{3'}$), 5.07 (br s, 2H, NH_2), 3.88 (s, 3H, N–CH_3), 2.07 (s, 3H, C–*C* H_3).

^{13}C{^{1}H} NMR (101 MHz, CD_3CN) δ 148.25 ($C_{2'}$), 144.55 (C_2) , 141.70 (C_5), 136.51 (C_3), 133.05 (C_4), 131.36 ($C_{1'}$), 127.33 (C_6), 123.59 ($C_{5'}$), 120.55 ($C_{4'}$), 117.17 ($C_{3'}$), 116.81 ($C_{6'}$), 44.94 (N-CH_3), 18.11 (C-*C*H_3).

HRMS m/z found: 230,1288; calcd for $C_{13}H_{16}N_30+$ (M–OTf)$^+$: 230,12879.

Pyridinium 4-nitro aminophenole 2f

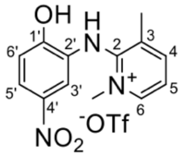

In a microwave vial, **1a** (946 mg, 3.24 mmol), 2-amino-4-nitrophenol (500 mg, 3.24 mmo), and $NaHCO_3$ (816 mg, 9.73 mmol) were dissolved in MeCN (10 mL). The reaction mixture was stirred at 80 °C for 18 h. It was then cooled to room temperature, and the solvent was removed *in vacuo*. The residue was extracted with CH_2Cl_2 (3 × 30 mL) and concentrated under reduced pressure. The resulting oil was dried under high vacuum for 18 h to afford the title compound **2f** as a light brown solid (1.456 g, 3.20 mmol, 98%).

^{1}H NMR (400 MHz, CD_3CN): δ 7.91 (d, J = 6.2 Hz, 1H, H_4), 7.84 (dd, J = 9.0, 2.8 Hz, 1H, $H_{5'}$), 7.77 (d, J = 7.3 Hz, 1H, H_6), 7.68 (d, J = 2.8 Hz, 1H, $H_{3'}$), 7.02 (dd, J = 6.2 Hz, 7,3 Hz 1H, H_5), 6.59 (d, J = 9.0 Hz, 1H, $H_{6'}$), 3.82 (s, 3H, N–CH_3), 2.03 (s, 1H, C–CH_3).

^{13}C{^{1}H} NMR (101 MHz, CD_3CN): δ 162.76 ($C_{4'}$), 153.51 (C_2), 144.62 (C_4), 141.10 (C_6), 137.75 (C_{phen}), 131.42 (C_{phen}), 130.26 (C_3), 122.77 ($C_{4'}$), 118.26 ($C_{3'}$), 116.40 (C_5), 115.85 ($C_{6'}$), 44.42 (N–CH_3), 19.56 (C–*C*H_3).

HRMS m/z: found: 260,10; calcd. for $C_{13}H_{14}N_3O_3$ (M–OTf)$^+$: 260.10297.

Pyridinium 5-nitro aminophenole 2g

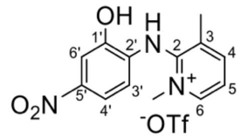

In a microwave vial, **1a** (473.1 mg, 1.62 mmol), 2-amino-4-nitrophenol (250 mg, 1.62 mmol), and $NaHCO_3$ (408.8 mg, 4.87 mmol) were dissolved in MeCN (10 mL). The reaction mixture was stirred at 80 °C for 18 h. It was then cooled to room temperature, and the solvent was removed *in vacuo*. The residue was extracted with CH_2Cl_2 (3 × 20 mL) and concentrated under reduced pressure. The resulting oil was dried under high vacuum for 18 h to afford the title compound **2g** as a light red/brown solid (609.91 mg, 1.49 mmol, 92%).

^{1}H NMR (400 MHz, CD_3CN): δ 7.78 (dd, J = 6.6, 1.7 Hz, 1H, H_6), 7.64 (dd, J = 8.7, 2.6 Hz, 1 $H_{4'}$), 7.57 (d, J = 2.6 Hz, 1H, $H_{6'}$), 7.54 (dd, J = 6.6, 6.2 Hz, 1H, H_4), 7.57–7.50 (m, 1H, H_5), 6.44 (d, J = 8.7 Hz, 1H, $H_{3'}$), 3.77 (s, 3H, N–CH_3), 1.89 (s, 3H, C–CH_3).

^{13}C{^{1}H} NMR (101 MHz, CD_3CN): δ 154.41 (C_3), 148.76 ($C_{1'}$), 144.48 ($C_{5'}$), 142.55 (C_4), 140.56 ($C_{2'}$), 140.09 (C_6), 129.12 (C_2), 116.75 ($C_{3'}$), 116.73($C_{4'}$), 112.40 (C_5), 108.30 ($C_{6'}$), 42.88 (N–CH_3), 19.97 (C–*C*H_3).

HRMS m/z found: 260,1029; calcd. for $C_{13}H_{14}N_3O_3$ (M–OTf)$^+$: 260.10297.

Pyridinium 5-methylaminophenol 2h

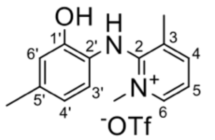

In a microwave vial, **1a** (592.1 mg, 2.03 mmol), 2-amino-4-nitrophenol (250 mg, 2.03 mmol), and $NaHCO_3$ (511.6 mg, 6.09 mmol) were dissolved in MeCN (10 mL). The reaction mixture was stirred at 80 °C for 18 h. It was then cooled to room temperature, and the solvent was removed *in vacuo*. The residue was extracted with CH_2Cl_2 (3 × 20 mL) and concentrated under reduced pressure. The resulting oil was dried under high vacuum for 18 h to afford the title compound **2h** as a light red/brown solid (703 mg, 1.89 mmol, 91%).

^{1}H NMR (400 MHz, , CD_3CN): δ 7.92 (dd, J = 6.6, 1.7 Hz, 1H, H_6), 7.84 (d, J = 7.4 Hz, 1H, H_5), 7.09 (dd, J = 7.4, 6.6 Hz, 1H, H_5), 6.95 (d, J = 8.0 Hz, 1H, $H_{3'}$), 6.78 (d, J = 1.9 Hz, 1H, $H_{6'}$), 6.73 (dd, J = 8.0, 1.9 Hz, 1H, $H_{4'}$), 3.81 (s, 3H, N–CH_3), 2.28 (s, 3H, C_{phen}–CH_3), 2.03 (s, 3H, C_{pyr}–CH_3)

^{13}C{^{1}H} NMR (101 MHz, CD_3CN): δ 153.53 ($C_{2'}$), 151.70 (C_2), 145.57 (C_6), 141.34 (C_4), 139.13 ($C_{5'}$), 129.34 (C_3), 126.40 ($C_{3'}$), 124.60 ($C_{1'}$), 121.92 ($C_{4'}$), 117.42 (C_5), 117.34 ($C_{6'}$), 44.91 (N–CH_3), 21.09 (C_{phenol}–*C*H_3), 19.02 (C_{pyr}–*C*H_3)

HRMS m/z found: 229.13; calcd. for $C_{14}H_{17}N_2O$ (M–OTf)$^+$: 229.13354.

Pyridinium 4-methoxyaminophenole 2i

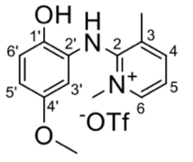

In a microwave vial, **1a** (1.05 g, 3.59 mmol 2-amino-3-methoxyphenol (500 mg, 3.59 mmol), and $NaHCO_3$ (905.53 mg, 10.78 mmol) were dissolved in MeCN (10 mL). The reaction mixture was stirred at 80 °C for 18 h. It was then cooled to room temperature, and the solvent was removed *in vacuo*. The residue was extracted with CH_2Cl_2 (3 × 30 mL) and concentrated under reduced pressure. The resulting oil was dried under high vacuum for 18 h to afford the title compound **2i** as a yellow/brown solid (1.355 g, 3.44 mmol, 95%).

^{1}H NMR (400 MHz, CD_3CN): δ 8.01 (d, J = 6.4 Hz, 1H, H_6), 7.92 (d, J = 7.5 Hz, 1H, H_4), 7.20 (dd, J = 7.5, 6.4 Hz, 1H, H_5), 6.87 (d, J = 8.8 Hz, 1H, $H_{6'}$), 6.72 (dd, J = 8.8, 3.0 Hz, 1H, $H_{5'}$), 6.64 (d, J = 3.0 Hz, 1H, $H_{3'}$), 3.86 (s, 3H, N–CH_3), 3.71 (s, 3H, C–OCH_3), 2.09 (s, 3H, C–CH_3)

^{13}C{^{1}H} NMR (101 MHz, CD_3CN): δ 154.53 ($C_{4'}$), 153.01 (C_2), 146.08 (C_4), 144.89 ($C_{2'}$), 141.72 (C_6), 130.59 (C_3), 127.79 ($C_{1'}$), 118.58 (C_5), 117.56 ($C_{6'}$), 113.27 ($C_{5'}$), 110.97 ($C_{3'}$), 56.36 (C–O*C*H_3), 45.08 (N–CH_3), 18.80 (C–*C*H_3)

HRMS m/z found: 245.1284; calcd. for $C_{14}H_{17}N_2O_2$ $(M–OTf)^+$: 245.12845.

2.3 Synthesis of complexes 3b–3i

Complex 3b

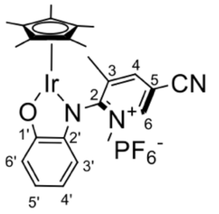

In a microwave vial, **2b** (80.5 mg, 0.207 mmol), $[IrCp^*Cl_2]_2$ (80.0 mg, 0.100 mmol), Na_2CO_3 (127.7 mg, 1.20 mmol), and $NaPF_6$ (337.3 mg, 2.01 mmol) were placed under a nitrogen atmosphere. CH_3CN (10 mL) was added, and the mixture was stirred at 50 °C for 18 h in the dark (vial wrapped in aluminum foil). After cooling to room temperature, the mixture was filtered, and the solvent was removed under reduced pressure. The crude solid was extracted with CH_2Cl_2 (5 × 50 mL), and the combined organic layers were concentrated *in vacuo* to afford **3b** as a dark-red solid (142 mg, 0.196 mmol, 97%). Crystals suitable for X-ray diffraction analysis were obtained by slow diffusion of Et_2O into a CH_2Cl_2 solution of **3b**.

^{1}H NMR (400 MHz, $(CD_3)_2SO$): δ 9.69 (d, J = 2.0 Hz, 1H, H_6), 9.12 (d, J = 2.0 Hz, 1H, H_4), 7.11 (dd, J = 8.2, 1.3 Hz, 1H, $H_{6'}$), 6.77 (ddd, J = 8.2, 7.0, 1.7 Hz, 1H, $H_{4'}$), 6.57 (ddd, J = 8.1, 7.0, 1.3 Hz, 1H, $H_{5'}$), 6.53 (dd, J = 8.1, 1.7 Hz, 1H, $H_{3'}$), 3.96 (s, 3H, N–CH_3), 2.04 (s, 3H, C–CH_3), 1.67 (s, 15H, Cp–CH_3).

^{13}C{^{1}H} NMR (101 MHz, $(CD_3)_2SO$): δ 163.44 ($C_{2'}$), 160.39 (C_2), 149.75 (C_4), 149.05 (C_6), 143.80 ($C_{1'}$), 135.43 (C_3), 121.02 ($C_{5'}$), 117.72 ($C_{4'}$), 115.26 ($C_{6'}$), 114.23 (C_5), 111.62 ($C_{3'}$), 107.72 (C≡N), 86.78 (C_{Cp}), 43.19 (N–CH_3), 16.12 (C–*C*H_3), 9.04 (Cp–CH_3).

HRMS m/z found: 566.1784**;** calcd. for $C_{24}H_{27}IrN_3O^+$ $(M–PF_6)^+$: 566.17779.

Elem. Anal. calcd. for $C_{24}H_{27}F_6IrN_3OP \times CH_2Cl_2$: C, 37.74; H, 3.67; N, 5.28%. Found: C, 37.93; H, 3.73; N, 5.60%.

Complex 3c

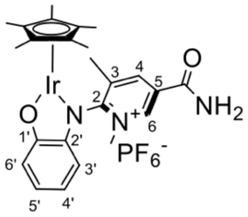

In a microwave vial, $[IrCp^*(Cl)_2]_2$ (80 mg, 0.1 mmol), **2c** (83.86 mg, 0.205 mmol), Na_2CO_3 (127.71 mg, 1.20 mmol), and $NaPF_6$ (337.30 mg, 2.01 mmol) were placed under a nitrogen atmosphere. CH_3CN (10 mL) was added, and the mixture was stirred at 50 °C for 18 h in the dark (vial wrapped in aluminum foil). After cooling to room temperature, the mixture was filtered, and the solvent was removed under reduced pressure. The crude solid was extracted with CH_2Cl_2 (5 × 50 mL), and the combined organic layers were concentrated *in vacuo* to afford **3c** as a dark-orange solid (137 mg, 0.184 mmol, 92%).

^{1}H NMR (400 MHz, CD_3CN): δ 9.06 (d, J = 2.1 Hz, 1H, H_6), 8.81 (d, J = 2.1 Hz, 1H, H_4), 7.12 (dd, J = 8.2, 1.3 Hz, 1H, $H_{6'}$), 6.82 (ddd, J = 8.2, 7.2, 1.4 Hz, 1H, $H_{5'}$), 6.62 (ddd, J = 7.8, 7.2, 1.3 Hz, 1H, $H_{4'}$), 6.37 (dd, J = 7.8, 1.4 Hz, 1H, $H_{3'}$), 4.01 (s, 3H, N–CH_3), 2.16 (s, 3H, C–CH_3), 1.68 (s, 15H, Cp–CH_3).

^{13}C{^{1}H} NMR (101 MHz, CD_3CN): δ 164.65 (C_{phen}), 163.54 (C_2), 160.80 (C_5), 146.63 (C_6), 144.74 (C_{phen}), 144.36 (C_6), 136.43 (C_3), 129.84 ($CONH_2$), 121.91 ($C_{5'}$), 118.55 ($C_{4'}$), 115.81 ($C_{6'}$), 111.74 ($C_{3'}$), 87.42 (C_{Cp}), 43.92 (N–CH_3), 16.79 (C–*C*H_3), 9.37 (Cp–CH_3)

HRMS m/z found: 584.1894; calcd. for $C_{24}H_{29}IrN_3O_2^+$ $(M–PF_6)^+$: 584.18835.

Elem. Anal. calcd. for $C_{24}H_{29}F_6IrN_3O_2P$: C, 39.56; H, 4.01; N, 5.77%. Found: C, 40.24; H, 3.60; N, 5.40%.

Complex 3d

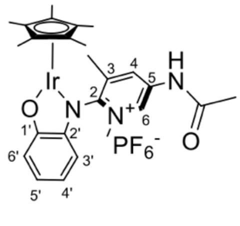

In a microwave vials, $[IrCp^*(Cl)_2]_2$ (80 mg, 0.105 mmol), **2d** (95.2 mg, 0.215 mmol), Na_2CO_3 (133.7 mg, 1.26 mmol) and $NaPF_6$ (350.01 mg, 2.10 mmol) are dissolved in CH_3CN under inert N_2 atmosphere. The reaction was stirred at 50 °C for 18h in the dark (aluminum foil). The mixture was let to cool down at room temperature and filtered. The solvent was removed under reduced pressure. The crude solid was extracted with CH_2Cl_2 (5 x 50 mL) and the solvent was removed *in vacuo* to yield ***3d*** as a red-brown solid (120 mg, 0.161 mmol, 76.87%).

¹H NMR (400 MHz, $(CD_3)_2SO$): δ = 10.92 (s, 1H, NH), 9.34 (d, J=2.5, 1H, H_6), 8.44 (d, J=2.5, 1H, H_4), 7.09 (dd, J=8.0, 1.2, 1H, $H_{6'}$), 6.74 (ddd, J = 7.7, 7.6 1.2 Hz, 1H, $H_{4'}$), 6.56 (ddd, J=8.0, 7.6, 1.4, 1H, $H_{5'}$), 6.42 (dd, J=7.7, 1.4, 1H, $H_{3'}$), 4.00 (s, 3H, N–CH_3), 2.22 (s, 3H, C–CH_3), 2.05 (s, 3H, NHCO–C*H_3*), 1.68 (s, 15H, Cp–CH_3).

$^{13}C\{^1H\}$ NMR (101 MHz, $(CD_3)_2SO$): δ = 169.67 (N*C*OCH_3), 163.54 ($C_{2'}$), 152.62 (C_4), 144.66 ($C_{1'}$), 136.31 ($C_{3'}$), 135.22 (C_2), 134.67 (C_4), 133.34 (C_6), 120.45 ($C_{4'}$), 117.64 ($C_{5'}$), 115.02 ($C_{6'}$), 110.99 ($C_{3'}$), 87.42 (*Cp–C*H_3), 43.03 (N–CH_3), 23.86 (NCO–*C*H_3), 16.32 (C–*C*H_3), 9.14 (Cp–CH_3).

HRMS m/z found: 598.2046; calcd. for $C_{25}H_{31}IrN_3O_2^+$ $(M–PF_6)^+$: 598,20400.

Elem. Anal. calcd. for $C_{25}H_{31}F_6IrN_3O_2P$: C, 40.43; H, 4.21; N, 5.66%. Found: C, 40.15; H, 4.24; N, 5.5%.

Complex 3e

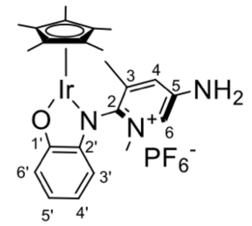

Pyridinium salt **2e** (80 mg, 0.210 mmol), $[IrCp^*(Cl)_2]_2$ (80.3 mg, 0.105 mmol), Na_2CO_3 (67.1 mg, 0.63 mmol) and $NaPF_6$ (354.2 mg, 0.211 mmol) were placed under inert N_2 atmosphere in a microwave vial. CH_3CN (10 mL) was added, and the reaction was stirred at 50 °C for 18 hours in the dark (under aluminum foil). The mixture was let cool down at room temperature and filtered. The solvent was removed under reduced pressure. The crude solid was extracted with CH_2Cl_2 (5 x 50 mL) and the solvent was removed *in vacuo* to yield ***3e*** as a dark red powder (116 mg, 0.163 mmol, 76.85%).

¹H NMR (400 MHz, CD_3CN): δ 7.83 (d, J = 2.7 Hz, 1H, H_6), 7.70 (d, J = 2.7 Hz, 1H, H_4), 7.07 (dd, J = 8.1, 1.3 Hz, 1H, $H_{6'}$), 6.78 (ddd, J = 8.1, 7.3, 1.5 Hz, 1H, $H_{5'}$), 6.60 (ddd, J = 7.8, 7.3, 1.3 Hz, 1H, $H_{4'}$), 6.35 (dd, J = 7.8, 1.5 Hz, 1H, $H_{3'}$), 5.17 (s, 2H, NH_2), 3.83 (s, 3H, N–CH_3), 2.02 (s, 3H, C–CH_3), 1.71 (s, 15H, Cp–CH_3).

$^{13}C\{^1H\}$ NMR (101 MHz, CD_3CN): δ 165.10 ($C_{2'}$), 146.42 ($C_{1'}$), 145.58 (C_5), 136.43 (C_2), 132.84 (C_4), 127.68 (C_6), 121.67 ($C_{5'}$), 118.71 ($C_{4'}$), 115.84 ($C_{6'}$), 111.73 ($C_{3'}$), 87.31 (C_{Cp}), 43.60 (N–CH_3), 17.02 (C–*C*H_3), 9.84 (Cp–CH_3).

HRMS m/z found: 556.1942; calcd. for $C_{23}H_{29}IrN_3O^+$ $(M–PF_6)^+$: 556.19344.

Elem. Anal. calcd. for $C_{23}H_{29}F_6IrN_3OP$: C, 39.43; H, 4.17; N, 6.00%. Found: C, 39.25; H, 4.46; N, 5.68%.

Complex 3f

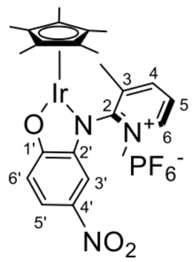

In a microwave vial, $[IrCp^*(Cl)_2]_2$ (70.0 mg, 0.088 mmol), **2f** (71.93 mg, 0.176 mmol), Na_2CO_3 (55.9 mg, 0.527 mmol), and $NaPF_6$ (295 mg, 1.76 mmol) were dissolved in CH_3CN (10 mL) under a nitrogen atmosphere. The reaction mixture was stirred at room temperature for 18 h. After cooling, the mixture was filtered and the filtrate was concentrated *in vacuo*. The residue was extracted with CH_2Cl_2 (5 × 50 mL), and the combined organic layers were concentrated to afford **3f** as a red solid (115.3 mg, 0.155 mmol, 89%).

^{1}H NMR (400 MHz, CD_3CN): δ 8.64 (td, J = 6.4, 1.4 Hz, 1H, H_6), 8.55 (dd, J = 7.8, 1.4 Hz, 1H, H_4), 7.82 (dd, J = 7.8, 6.4 Hz, 1H, H_5), 7.82 (dd, J = 9.0, 2.5 Hz, 1H, $H_{5'}$), 7.24 (d, J = 2.5 Hz, 1H, $H_{6'}$), 7.15 (d, J = 9.0 Hz, 1H, $H_{3'}$), 4.01 (s, 3H, N–CH_3), 2.16 (s, 3H, C–CH_3), 1.65 (s, 15H, Cp–CH_3).

^{13}C{^{1}H} NMR (101 MHz, CD_3CN): δ 170.87 ($C_{4'}$), 158.08 (C_2), 149.75 (C_4), 145.54($C_{2'}$), 145.10 (C_6), 140.03 ($C_{1'}$), 137.02 (C_3), 125.27 (C_5), 119.15 ($C_{5'}$), 114.69 ($C_{3'}$), 107.26 ($C_{6'}$), 88.75 (C_{Cp}), 44.11 (N–CH_3), 16.94 (C–*C*H_3), 9.74 (Cp–CH_3).

HRMS m/z found: 586.1684; calcd. for $C_{23}H_{27}IrN_3O_3^+$ $(M–PF_6)^+$: 586.16762.

Elem. Anal. calcd. for $C_{23}H_{27}F_6IrN_3O_3P$: C, 37.81; H, 3.72; N, 5.75%. Found: C, 37.4; H, 3.7; N, 6.02%.

Complex 3g

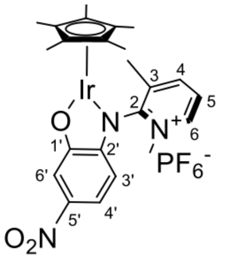

In a microwave vial, **2g** (71.9 mg, 0.175 mmol), $[IrCp^*(Cl)_2]_2$ (70.0 mg, 0.087 mmol), Na_2CO_3 (55.9 mg, 0.527 mmol) and $NaPF_6$ (295 mg, 1.76 mmol) were placed under an inert N_2 atmosphere in a microwave vial. CH_3CN (10 mL) was added, and the reaction mixture was stirred at room temperature for 36 h in the dark (wrapped in aluminum foil). The mixture was then cooled to room temperature and filtered. After removal of all volatiles, the crude residue was extracted with CH_2Cl_2 (5 × 50 mL), and the combined organic layers were concentrated *in vacuo* to give the title compound **3g** as a red solid (107.3 mg, 0.139 mmol, 82%). Suitable crystals for X-ray diffraction analysis were obtained by slow diffusion of Et_2O into a CH_2Cl_2 solution of **3g**.

^{1}H NMR (400 MHz, CD_3CN): δ 8.64 (d, J = 6.2 Hz, 1H, H_6), 8.55 (d, J = 7.9 Hz, 1H, H_4), 7.89 (d, J = 2.4 Hz, 1H, $H_{6'}$), 7.83 (dd, J = 7.9, 6.2 Hz, 1H, H_5), 7.59 (dd, J = 8.8, 2.4 Hz, 1H, $H_{4'}$), 6.43 (d, J = 8.8 Hz, 1H, $H_{3'}$), 3.97 (s, 3H, N–CH_3), 2.14 (s, 3H, C–CH_3), 1.65 (s, 15H, Cp–CH_3).

^{13}C{^{1}H} NMR (101 MHz, CD_3CN): δ 163.92 ($C_{1'}$), 152.11 ($C_{5'}$), 149.71 (C_4), 144.90 (C_6), 142.57 ($C_{2'}$), 136.95 (C_3), 125.29 (C_5), 115.41 ($C_{4'}$), 110.33 ($C_{6'}$), 110.30 (C_2), 88.73 (C_{Cp}) 44.10 (N–CH_3), 16.93 (C–*C*H_3), 9.70 (Cp–CH_3).

HRMS m/z found: 586.1683; calcd. for $C_{23}H_{27}IrN_3O_3^+$ $(M–PF_6)^+$: 586.16762.

Elem. Anal. calcd. for $C_{23}H_{27}F_6IrN_3O_3P \times 0.25\ CH_2Cl_2$: C, 37.14; H, 3.69; N, 5.59%. Found: C, 37.09; H, 3.58; N, 5.40%.

Complex 3h

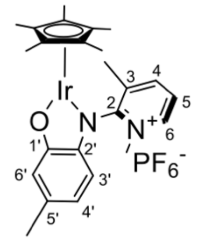

In a microwave vial, **2h** (80.0 mg, 0.211 mmol), $[IrCp^*(Cl)_2]_2$ (85.9 mg, 0.106 mmol, 1.0 equiv), Na_2CO_3 (67.2 mg, 0.634 mmol) and $NaPF_6$ (355.1 mg, 2.11 mmol) were placed under an inert N_2 atmosphere in a microwave vial. CH_3CN (10 mL) was added, and the reaction mixture was stirred at room temperature for 36 h in the dark (wrapped in aluminum foil). The mixture was then cooled to room temperature and filtered. After removal of all volatiles, the crude residue was extracted with CH_2Cl_2 (5 × 50 mL), and the combined organic layers were concentrated *in vacuo* to give the title compound **3h** as a red solid (143.3 mg, 0.200 mmol, 95%).

^{1}H NMR (400 MHz, CD_3CN): δ 8.58 (d, J = 6.2 Hz, 1H, H_6), 8.48 (d, J = 7.8 Hz, 1H, H_4), 7.75 (dd, 7.9, 6.2 Hz, 1H, H_5), 6.94 (d, J = 1.8 Hz, 1H, $H_{6'}$), 6.48 (dd, J = 8.0, 1.8 Hz, 1H, $H_{4'}$), 6.22 (d, J = 8.0 Hz, 1H, $H_{3'}$), 3.96 (s, 3H, N–CH_3), 2.29 (s, 3H, C_{phen}–CH_3), 2.12 (s, 3H, C_{pyr}–CH_3), 1.66 (s, 15H, Cp–CH_3)

^{13}C{^{1}H} NMR (101 MHz, CD_3CN): δ 165.12 (C_{phen}), 159.70 (C_2), 148.93 (C_4), 144.21 (C_{phen}), 143.10 (C_6), 136.98 (C_3), 131.99 ($C_{5'}$), 124.49 (C_5), 111.35 ($C_{3'}$), 87.37 (C_{Cp}), 43.72 (N-CH_3), 20.67 (C_{phen}–CH_3), 16.89 (C–CH_3), 9.69 (Cp–CH_3)

HRMS m/z found: 555.1992; calcd. for $C_{24}H_{30}IrN_2O^+$ $(M–PF_6)^+$: 555.19819.

Elem. Anal. calcd. for $C_{24}H_{30}F_6IrN_2OP$: C, 41.20; H, 4.32; N, 4.00%. Found: C, 41.34; H, 4.31; N, 4.11%

Complex 3i

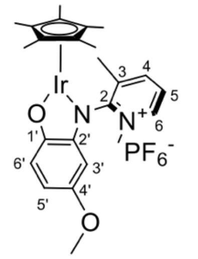

In a microwave vial, **2i** (80.0 mg, 0.203 mmol), $[IrCp^*(Cl)_2]_2$ (82.4 mg, 0.101 mmol), Na_2CO_3 (64.5 mg, 0.609 mmol) and $NaPF_6$ (340.7 mg, 2.04 mmol) were placed under an inert N_2 atmosphere in a microwave vial. CH_3CN (10 mL) was added, and the reaction mixture was stirred at room temperature for 36 h in the dark (wrapped in aluminum foil). The mixture was then cooled to room temperature and filtered. After removal of all volatiles, the crude residue was extracted with CH_2Cl_2 (5 × 50 mL), and the combined organic layers were concentrated *in vacuo* to give the title compound **3i** as a red solid (145.4 mg, 0.198 mmol, 98%). Suitable crystals for X-ray diffraction analysis were obtained by slow diffusion of Et_2O into a CH_2Cl_2 solution of **3i**.

^{1}H NMR (400 MHz, CD_3CN): δ 8.59 (d, J = 6.2 Hz, 1H, H_6), 8.49 (d, J = 7.8 Hz, 1H, H_4), 7.76 (dd, J = 7.8 ,6.2 Hz, 1H, H_5), 7.00 (d, J = 8.8 Hz, 1H, $H_{6'}$), 6.51 (dd, J = 8.8, 2.7 Hz, 1H, $H_{5'}$), 5.94 (d, J = 2.7 Hz, 1H, $H_{3'}$), 3.98 (s, 1H, N–CH_3), 3.58 (s, 1H, O–CH_3), 2.14 (s, 1H, C–CH_3), 1.65 (s, 15H, Cp–CH_3).

^{13}C{^{1}H} NMR (101 MHz, CD_3CN): δ 159.68 (C_{phen}), 159.35 (C_2), 154.31 ($C_{4'}$), 149.08 (C_4), 145.46 ($C_{1'}$), 144.37 (C_6), 136.95 (C_3), 124.58 (C_5), 115.13 ($C_{6'}$), 107.09 ($C_{5'}$), 97.47 ($C_{3'}$), 87.37 (C_{Cp}) 56.48 (O–CH_3), 43.84 (N–CH_3), 16.99 (C–$\mathit{C}H_3$), 9.70 (Cp–CH_3).

HRMS m/z found: 571.194; calcd. for $C_{24}H_{30}IrN_2O_2^+$ (M–PF_6)$^+$: 571.1931.

Elem. Anal. calcd. for $C_{24}H_{30}F_6IrN_2O_2P$ × 0.5 CH_2Cl_2: C, 38.81; H, 4.12; N, 3.69%. Found: C, 38.76; H, 4.03; N, 3.81%.

3 ELECTROCHEMICAL ANALYSIS

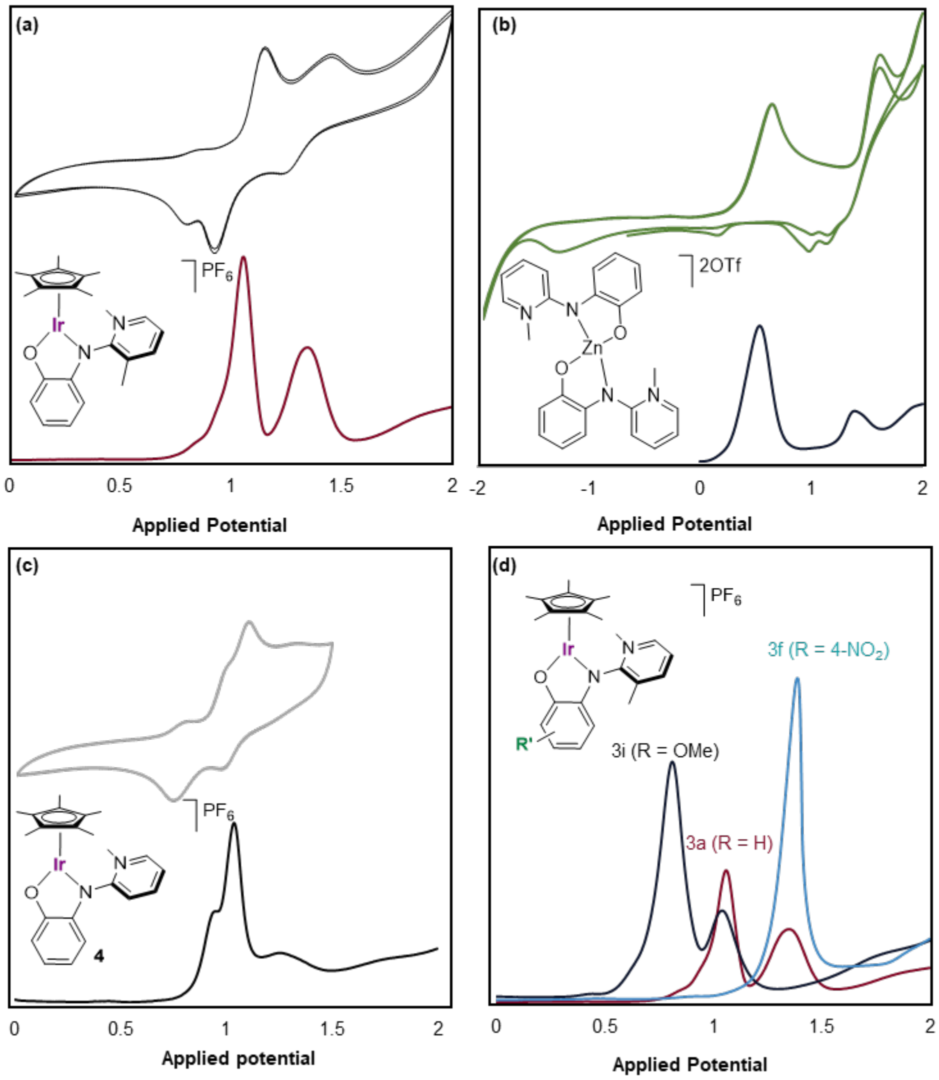

Figure S1: Cyclic voltammetry (CV) and normal pulse voltammetry (NPV) plots of (a) iridium complex **3a**, (b) zinc complex **Zn1,** (c) iridium complex **4** lacking the methyl group on the pyridinium unit, and (d) superimposed NPV of complexes **3a**, **3i**, and **3f**. All potentials vs. Fc^{+}/Fc in MeCN, $(Bu_4N)PF_6$ as supporting electrolyte.

4 DFT CALCULATIONS

4.1 GEOMETRY OPTIMIZATION SET UP

All ground-state structures were optimised with ORCA 6.0[S4] using the B3LYP[S5,S6] functional augmented by the Grimme D3(BJ)[S7,S8] dispersion correction. A def2-TZVP[S9] basis set was employed for every element together with the matching def2/J auxiliary basis; Coulomb and exact-exchange integrals were evaluated with the RIJCOSX[S10] approximation. Implicit solvation by DMSO ($\varepsilon = 46.8$) was included throughout via the CPCM model[S4,S11,S12].

Tight SCF convergence criteria were applied (energy change < 10^{-8} Eh; RMS density change < 10^{-7}) and a maximum of 500 SCF iterations was allowed. Optimisations used analytic gradients and were deemed converged when the largest Cartesian gradient component dropped below 3×10^{-4} Eh a_0^{-1}. Each stationary point was confirmed as a true minimum by an analytic frequency calculation at the same level of theory (no imaginary frequencies). Zero-point energies and 298 K thermal corrections reported in the main text derive from these frequency runs.

All jobs ran on 16 CPU cores (%pal nprocs 16 end) with 8 GB RAM per core (%maxcore 8000).

Example of orca input for geometry optimization

```
! B3LYP D3BJ def2-TZVP def2/J RIJCOSX TIGHTSCF Opt Freq Normalprint CPCM(DMSO)

%pal nprocs 16 end

%base "B3LYPTZVP_OptFreq"

%maxcore 8000

%scf

  MaxIter 500

end

* xyzfile 1 1 molecule.xyz
```

4.2 SCF AND POPULATION ANALYSIS SET UP

Single-point calculations on the optimised geometries were carried out at the same B3LYP[S5,S6] -D3(BJ) [7,8]/def2-TZVP [9] /CPCM(DMSO) [S4,S11,S12] level, but with extended print options to obtain detailed

electronic information (MOs, overlap matrix, Loewdin[S13] and fragment charges, Wiberg/Loewdin bond orders, orbital populations). The Loewdin bond-order threshold was set to 0.05.

4.3 TD-DFT CALCULATION SET UP

Vertical singlet excitation energies were obtained by time-dependent DFTS[4] at the same level of theory. Thirty lowest-energy roots were computed (NROOTS 30). Solvent, basis set, dispersion, RIJCOSX[S10] and SCF settings were identical to those used in the SCF step.

Example of input for SCF, population analysis and TD-DFT

```
#
! B3LYP D3BJ def2-TZVP def2/J RIJCOSX TIGHTSCF Normalprint CPCM(DMSO)
%pal nprocs 16 end

%output
Print[ P_Basis ] 2
Print[ P_MOs ] 2
Print[ P_Overlap ] 1
Print[P_NPA] 1
End

#Population analysis
%output
  Print[ P_AtCharges_M  ] 1           # Print atomic charges
  Print[ P_OrbCharges_M ] 1           # Print orbital charges
  Print[ P_ReducedOrbPop_M ] 1   # Print reduced orb. Charges
  Print[ P_AtPopMO_M    ] 1           # Print atomic charges in each MO
  Print[ P_OrbPopMO_M   ] 1          # Print orbital populaiton for each MO
  Print[ P_ReducedOrbPopMO_M] 1 # Print reduced orbital pop for each MO
  Print[ P_FragPopMO_M  ] 1            # Print the fragment  population for for each MO
end

%method
  LOEWDIN_BONDORDERTHRESH 0.05
end
```

```
%plots
dim1 100
dim2 100
dim3 100
Format Gaussian_Cube
ElDens("electrondensity");
end

%TDDFT
  NROOTS   30
  DONTO    TRUE
END

%base "B3LYPTZVP_NPA_TDDFT"
%maxcore 8000
%scf
        MaxIter 500
end
* xyzfile 1 1 molecule_opt.xyz
```

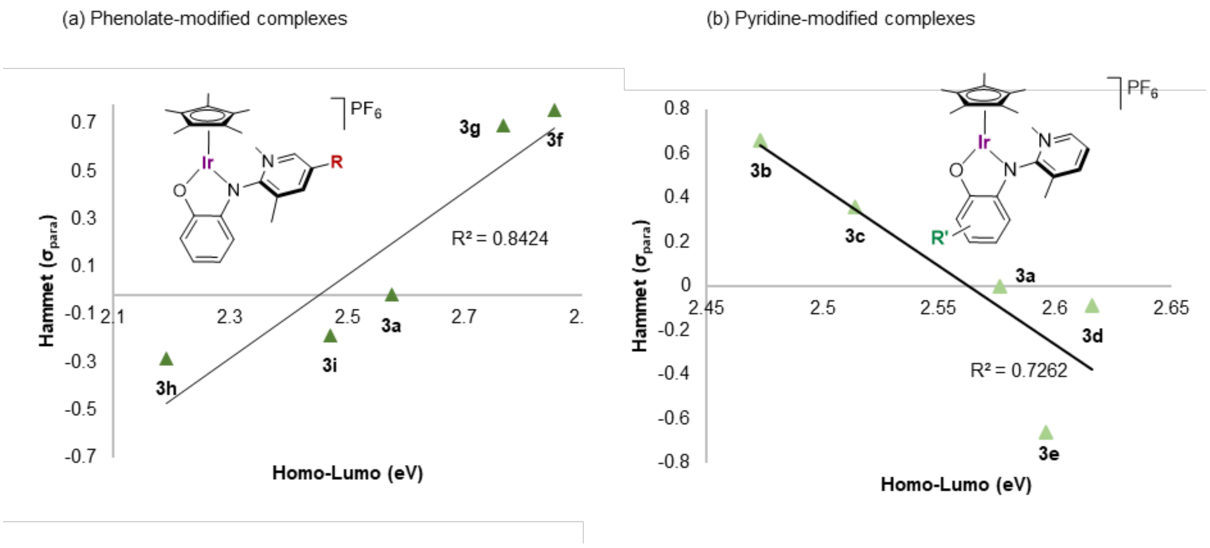

Figure S2: Relationship between Hammet parameter (σ_{para}) and the calculated energy gap (eV) between HOMO and LUMO (a) for the pyridine-modified complexes (b) for the aminophenolate-modified complexes

Table S1: Computed TD-DFT absorption band with B3LYP-D3(BJ)/def2-TZVP/CPCM(DMSO) level and measured λ_{max}

Complex	TD-DFT computed values DMSO (nm)	Measured (nm)	Hammet parameter (σ_{para})
**3a**	427	430	0
**3b**	425	421	0.66
**3c**	418	420	0.36
**3d**	425	420	-0.09
**3e**	428	422	-0.66
**3f**	415	418	0.78
**3g**	421	424	0.71
**3h**	431	430	-0.17
**3i**	447	459	-0.27

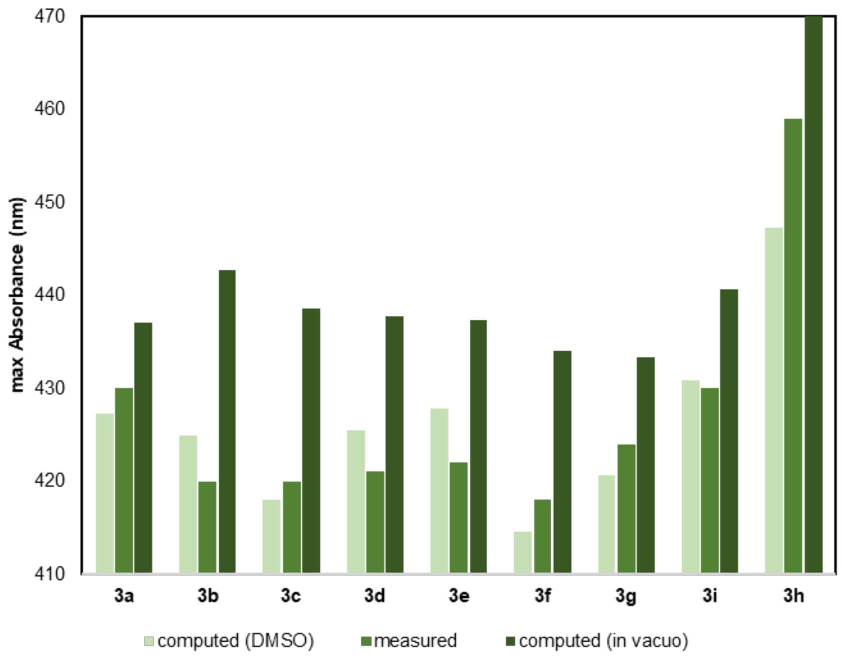

Figure S3: Computed TD-DFT absorption band with B3LYP-D3(BJ)/def2-TZVP level with CPCM(DMSO) and *in vacuo* and measured λ_{max}.

5 HYDRIDE PREPARATION

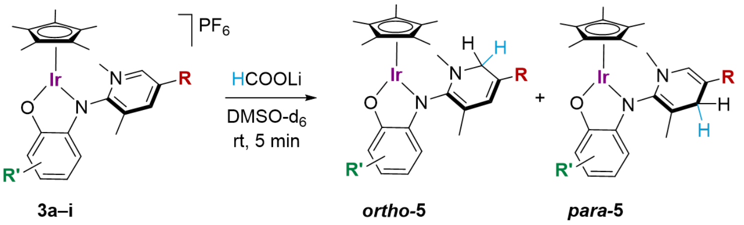

Scheme S1: *In situ* formation of hydrides complexes upon addition of HCOOLi.H_2O (0.20 mmol) in DMSO-d_6 (0.5 mL) containing the desired complex (0.02 mmol)

In an NMR tube, the desired complex (0.02 mmol) and HCOOLi.H_2O (0.20 mmol) were dissolved in DMSO-d_6 (0.5 mL) and HCOOLi.H_2O (0.20 mmol). ^{1}H-NMR spectra was recorded after 1 hour and an additional ^{1}H -NMR and ^{1}H -^{1}H NOEY spectra were recorded after 6 hours.

5.1 SPECTRA OF *PARA*- AND *ORTHO*-HYDRIDES

Spectroscopic data for *para*-5b and *ortho*-5b

^{1}H NMR (400 MHz, CD_3CN): δ 6.91 – 6.79 (m, 4H, $H_{para/ortho\text{-}phenol}$), 6.78 (s, 1H, H_4), 6.75 (s, 1H, H_6), 6.65 – 6.49 (m, 4H, $H_{para/ortho\text{-}phenol}$), 4.14 (d, J = 12.5 Hz, 1H, H_a of CH_2) 4.11 (d, J = 12.5 Hz, 1H, H_b of CH_2), 3.34 (d, J = 17.8 Hz, 1H, H_a of CH_2), 3.29 (d, J = 17.8 Hz, 1H, H_b of CH_2), 2.54 (s, 3H, N–CH_3), 2.47 (s, 3H, N–CH_3), 1.76 (s, 15H, Cp–CH_3), 1.74 (s, 15H, Cp–CH_3), 1.35 (s, 3H, C–CH_3), 1.25 (s, 3H, C–CH_3).

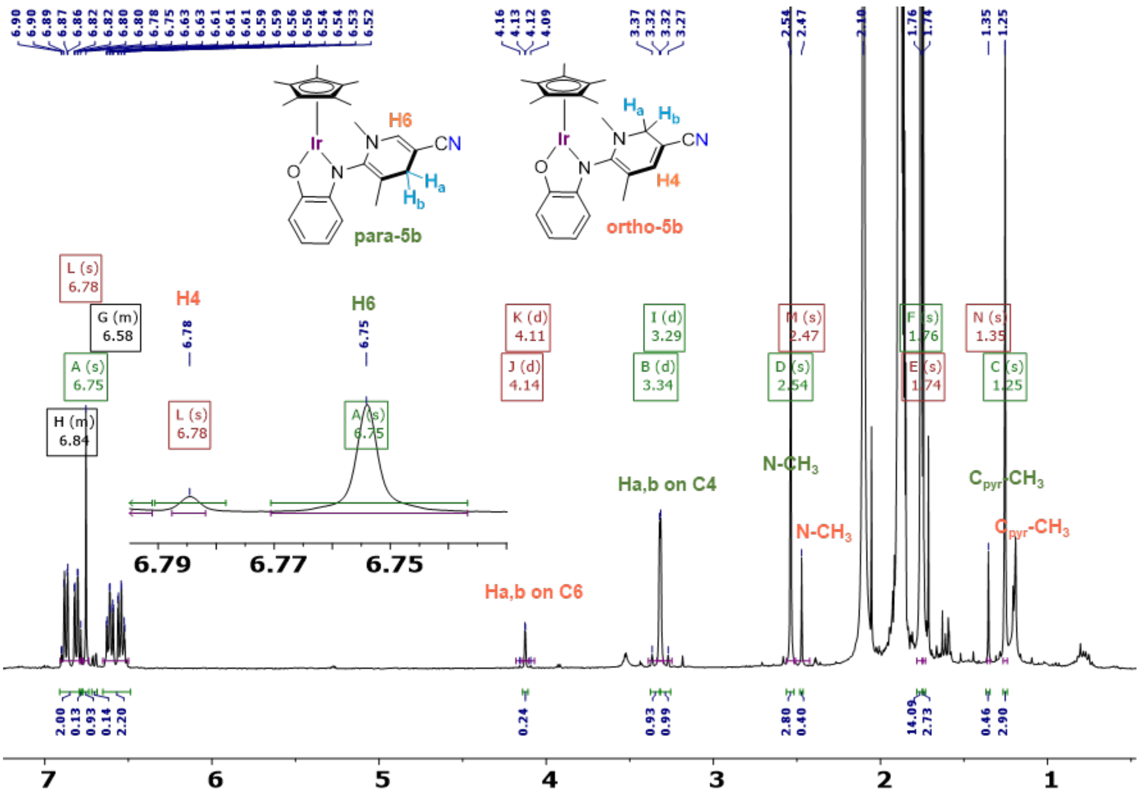

Figure S4: ^{1}H NMR spectrum (CD_3CN, 300 MHz) of ***ortho*-5b** and ***para*-5b.**

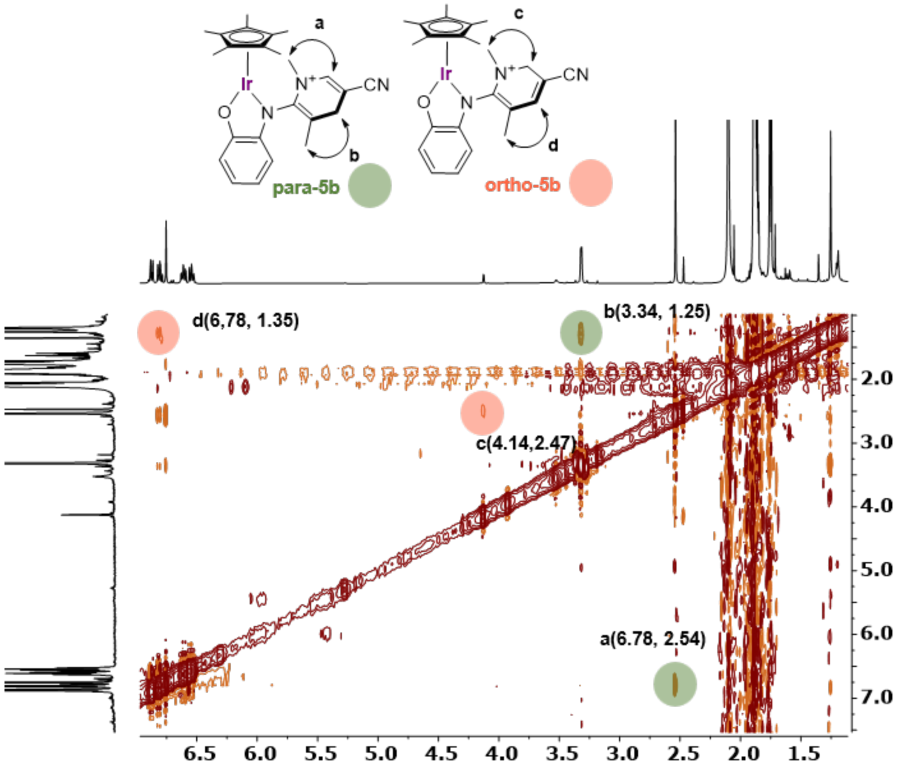

Figure S5: ^{1}H-^{1}H NOESY NMR spectrum (CD_3CN, 300 MHz) of ***ortho*-5b** and ***para*-5b** indicating the spatial proximity of the C^6H_2 protons with the NCH_3 group in ***ortho*-5b** (red spheres), and of the C^4H_2 protons with the CCH_3 group in ***para*-5b** (green spheres).

Spectroscopic data for *para*-5c and *ortho*-5c

^{1}H NMR (300 MHz, $(CD_3)_2SO$): δ 7.12 (s, 1H, H_6), 6.93 (dd, J = 7.8, 1.5 Hz, 2H, $H_{para/ortho\text{-}phenol}$), 6.79 (dd, J = 7.8, 1.5 Hz, 2H, $H_{para/ortho\text{-}phenol}$), 6.69 (s, 2H, NH2 and H_4), 6.66 – 6.48 (m, 4H, $H_{para/ortho\text{-}phenol}$), 4.26 (d, J = 12.6 Hz, 1H, H_a of CH_2), 4.11 (d, J = 12.6 Hz, 1H, H_b of CH_2), 3.35 (d, J = 17.5 Hz, 1H, H_a of CH_2), 3.28 (q, J = 17.5 Hz, 1H, H_b of CH_2), 2.59 (s, 3H, N–CH_3), 2.43 (s, 3, N–CH_3H), 1.80 (s, 15H, Cp–CH_3), 1.79 (s, 15H, Cp–CH_3), 1.40 (s, 1H, C–CH_3), 1.31 (s, 3H, C–CH_3).

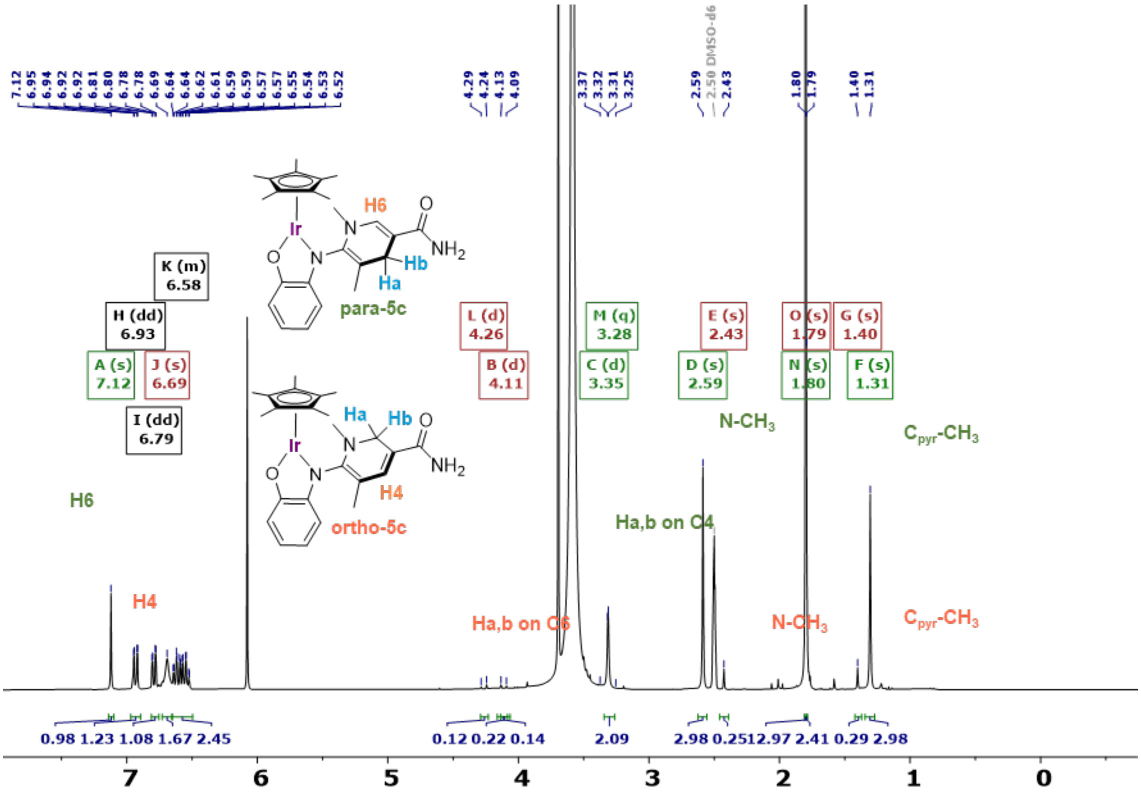

Figure S6: ^{1}H NMR spectrum (DMSO-d_6, 300 MHz) of ***ortho*-5c** and ***para*-5c.**

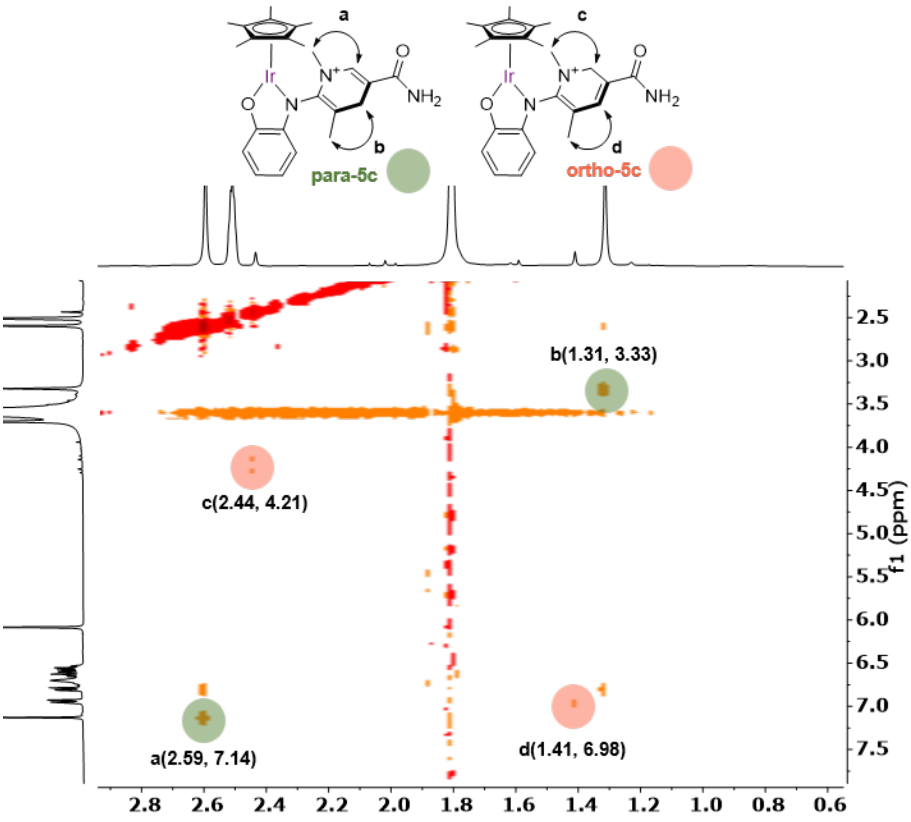

Figure S7: ^{1}H-^{1}H NOESY NMR spectrum (DMSO-d_6, 300 MHz) of ***ortho*-5c** and ***para*-5c** indicating the spatial proximity of the C^6H_2 protons with the NCH_3 group in ***ortho*-5c** (red spheres), and of the C^4H_2 protons with the CCH_3 group in ***para*-5c** (green spheres).

Spectroscopic data for *para*-5d and *ortho*-5d

^{1}H NMR (300 MHz, $(CD_3)_2SO$) δ 9.27 (s, 1H, NH), 8.75 (s, 1H, NH), 6.92 (dd, J = 7.8, 1.4 Hz, 2H, $H_{para\text{-}phenol}$), 6.87 – 6.78 (m, 4H, $H_{para/ortho\text{-}phenol}$), 6.77 (s, 1H, H6), 6.66 – 6.47 (m, 6H, $H_{para/ortho\text{-}phenol}$), 6.27 (s, 1H, H4), 4.02 (t, J = 13.2 Hz, 1H, H_a of CH_2), 3.97 (d, J = 13.2 Hz, 1H, H_b of CH_2), 3.29 (d, J = 18.4 Hz, 1H, H_a of CH_2), 3.25 (d, J = 18.4 Hz, 1H, H_b of CH_2), 2.44 (s, 3H, N–CH_3), 2.28 (s, 3H, N–CH_3), 1.95 (s, 3H, CO–CH_3), 1.91 (s, 3H, CO–CH_3), 1.80 (s, 15H, Cp–CH_3), 1.80 (s, 15H, Cp–CH_3), 1.38 (s, 3H, C–CH_3), 1.24 (s, 3H, C–CH_3).

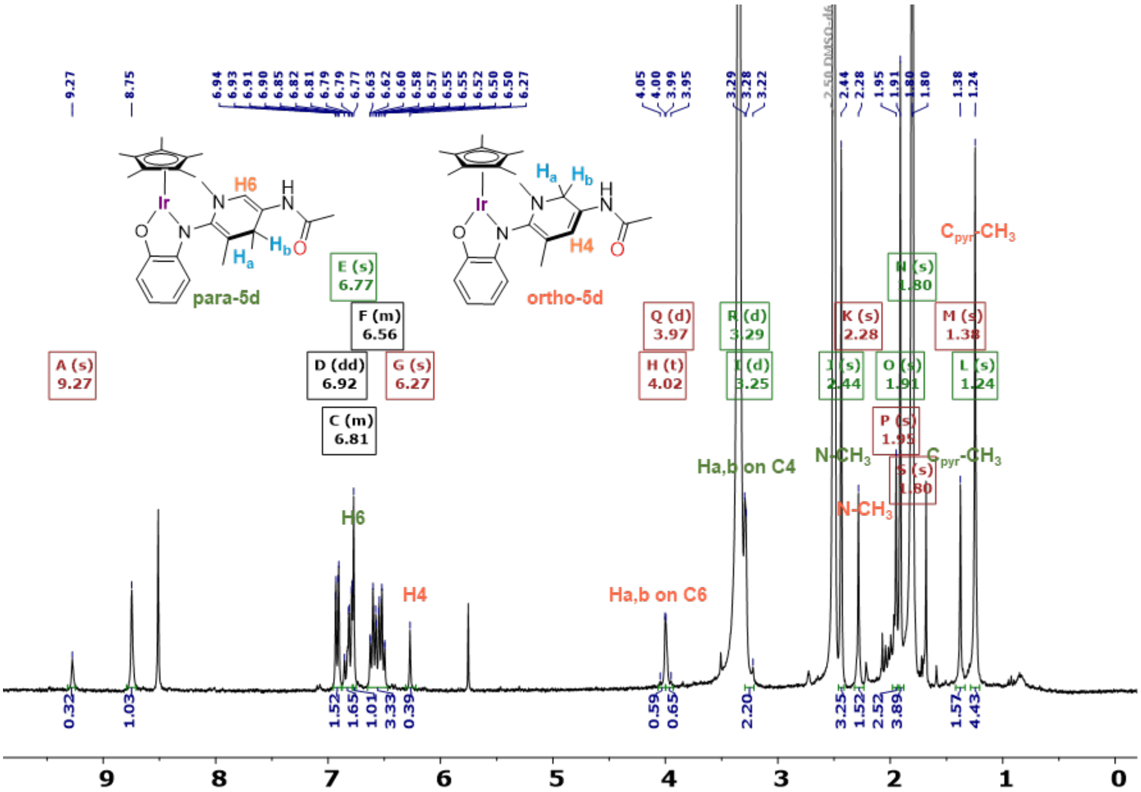

Figure S8: ^{1}H NMR spectrum (DMSO-d$_6$, 300 MHz) of ***ortho*-5d** and ***para*-5d.**

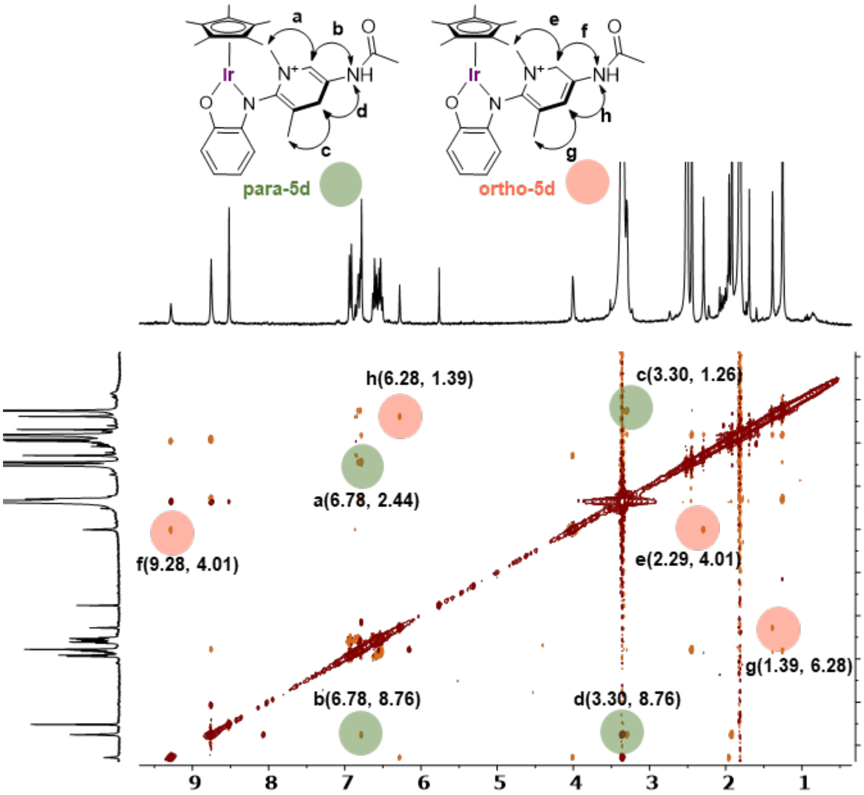

Figure S9: ^{1}H-^{1}H NOESY NMR spectrum (DMSO-d$_6$, 300 MHz) of ***ortho*-5d** and ***para*-5d** indicating the spatial proximity of the C^{6}H$_2$ protons with both the NCH$_3$ and the NHAc group

in ***ortho*-5d** (red spheres), and of the C^4H_2 protons with the CCH_3 and the NHAc group in ***para*-5d** (green spheres).

Spectroscopic data for *para*-5f

^{1}H NMR (300 MHz, $(CD_3)_2SO$) 7.66 – 7.55 (m, 2H, H_{phenol}), 7.02 (dt, J = 8.8, 1.3 Hz, 1H, H_{phenol}), 6.02 (d, J = 7.7 Hz, 1H, H_6), 4.45 (td, J = 7.7, 3.8 Hz, 1H, H_5), 3.27 (dd, J = 18.0, 2.1 Hz, 1H, H_a of CH_2), 3.18 (dd, J = 18.0, 2.1 Hz, 1H, H_b of CH_2), 2.45 (s, 3H, N–CH_3), 1.80 (s, 15H, Cp–CH_3), 1.24 (s, 3H, C–CH_3).

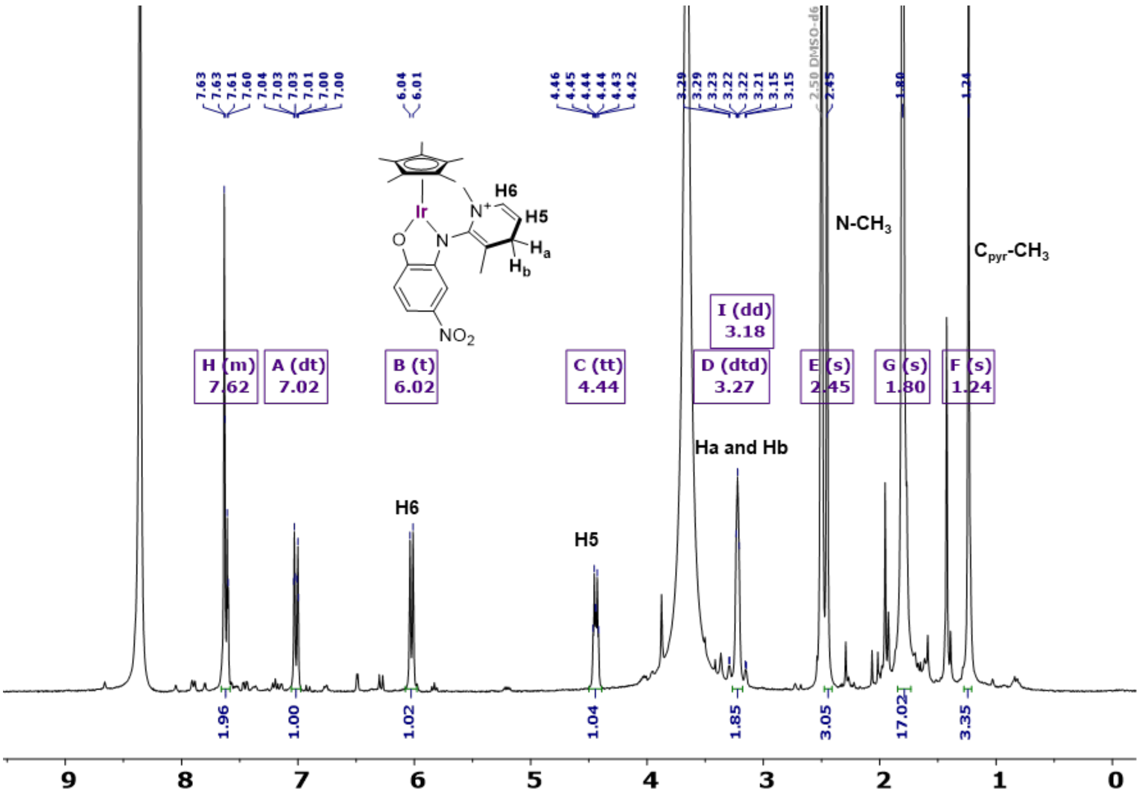

Figure S10: 1H NMR spectrum (DMSO-d6, 300 MHz) of ***para*-5f** with resonances for HCOOLi at 8.35 ppm and for H2O at 3.81 ppm.

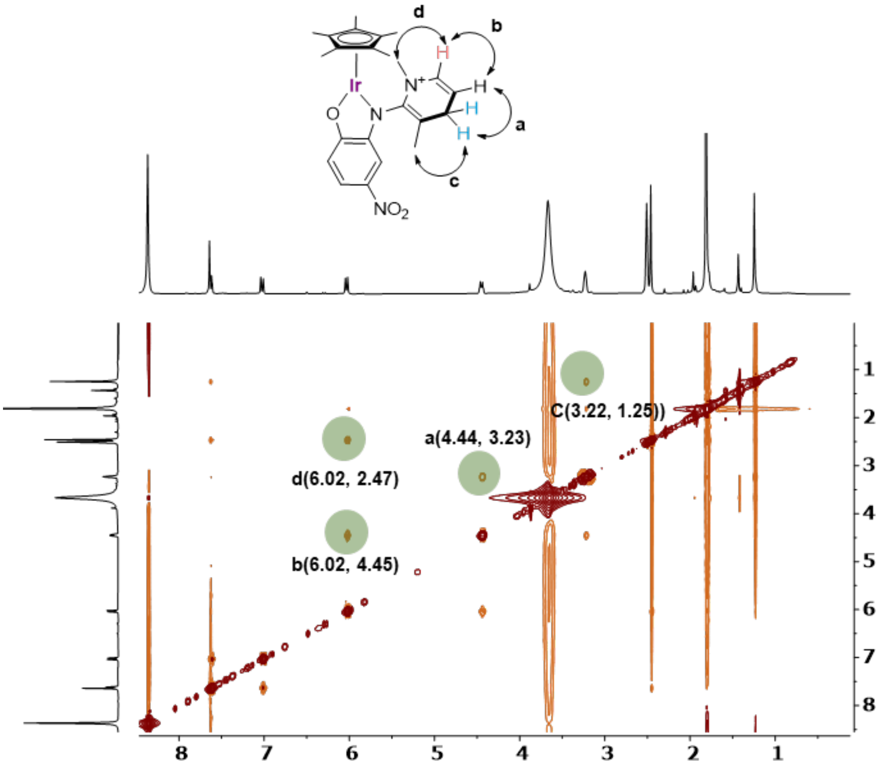

Figure S11: ¹H-1H NOESY NMR spectrum (DMSO-d6, 300 MHz) of ***para*-5f** indicating the spatial proximity of H5 with H6 and both CH_2 protons of C4.

Spectroscopic data for *para*-5g

¹H NMR (300 MHz, $(CD_3)_2SO$) δ 7.68 (d, J = 2.5 Hz, 1H, $H_{3'}$), 7.60 (dd, J = 8.8, 2.5 Hz, 1H, $H_{5'}$), 6.91 (d, J = 8.8 Hz, 1H, $H_{6'}$), 6.02 (dd, J = 7.9, 1.2 Hz, 1H, H_6), 4.44 (dt, J = 7.9, 2.9 Hz, 1H, H_5), 3.24 (dd, J = 19.5, 2.9 Hz, 1H, H_a of CH_2), 3.16 (dd, J = 19.5, 2.9 Hz, 1H, H_b of CH_2), 2.44 (s, 3H, N–CH_3), 1.81 (s, 15H, Cp–CH_3), 1.20 (s, 3H, C–CH_3).

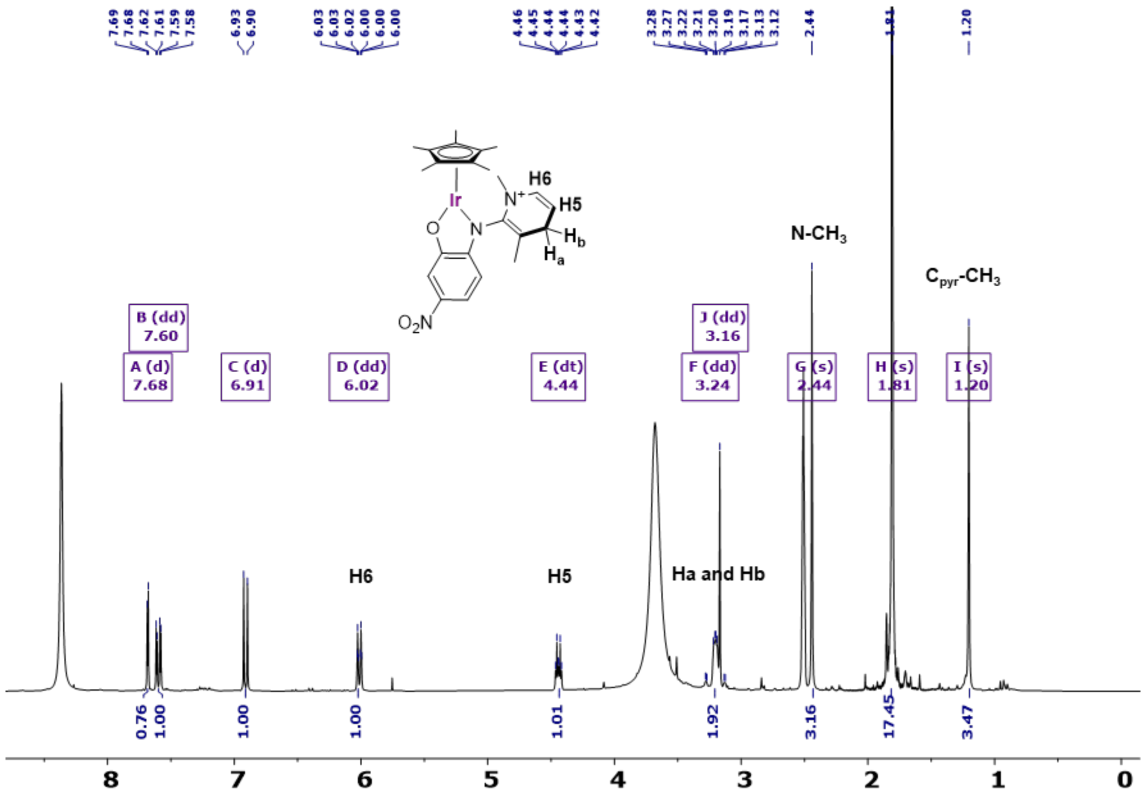

Figure S12: ^{1}H NMR spectrum (DMSO-d6, 300 MHz) of ***para*-5g** with resonances for HCOOLi at 8.35 ppm and for H_2O at 3.81 ppm.

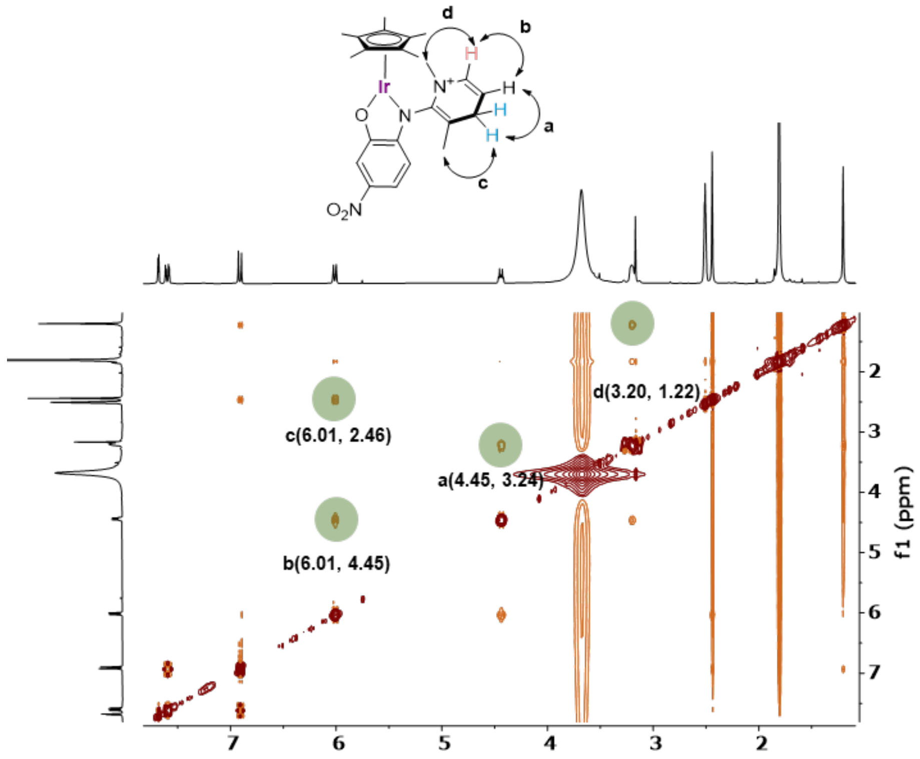

Figure S13: ^{1}H-1H NOESY NMR spectrum (DMSO-d6, 300 MHz) of ***para*-5g** indicating the spatial proximity of H5 with H6 and both CH_2 protons of C4.

Spectroscopic data for *para*-5h

^{1}H NMR (300 MHz, $(CD_3)_2SO$) δ 6.76 – 6.66 (m, 2H, H_{phenol}), 6.38 (ddd, J = 8.0, 1.8, 0.7 Hz, 1H, H_{phenol}), 5.97 (dd, J = 7.9, 1.3 Hz, 1H, H_6), 4.37 (dt, J = 7.9, 3.2 Hz, 1H, H_5), 3.22 (dd, J = 17.6, 3.2 Hz, 1H, H_a of CH_2), 3.15 (dd, J = 17.6, 3.2 Hz, 1H, H_a of CH_2), 2.41 (s, 3H, N-CH_3), 2.19 (s, 3H, C_{phenol}-CH_3), 1.80 (s, 15H, Cp–CH_3), 1.19 (s, 3H, C_{pyr}-CH_3).

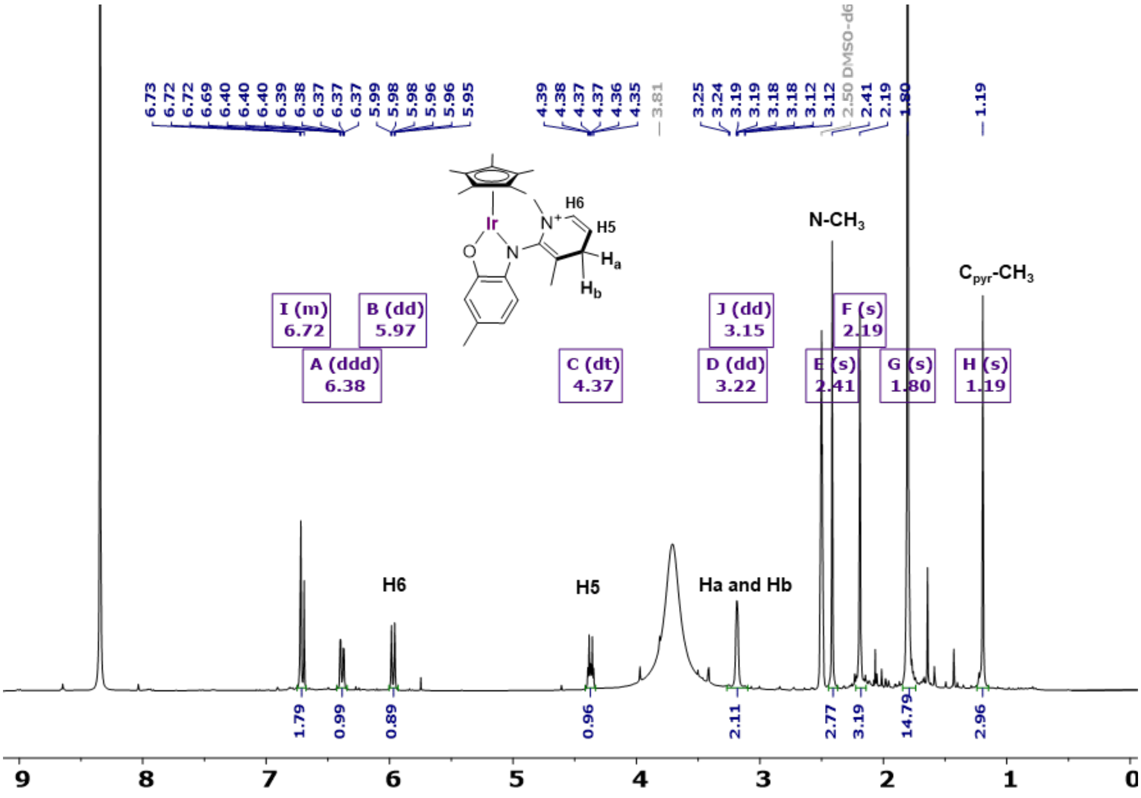

Figure S14: ^{1}H NMR spectrum (DMSO-d_6, 300 MHz) of ***para*-5h** with resonances for HCOOLi at 8.35 ppm and for H_2O at 3.81 ppm.

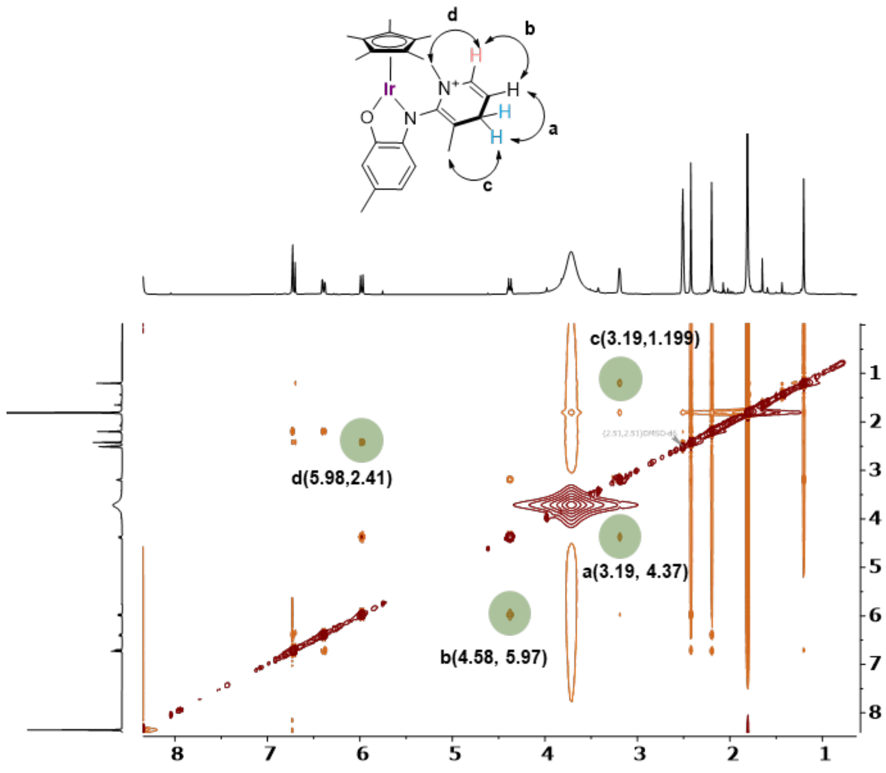

Figure S15: ^{1}H-1H NOESY NMR spectrum (DMSO-d$_6$, 300 MHz) of ***para*-5h** indicating the spatial proximity of H5 with H6 and both CH_2 protons of C4.

Spectroscopic data for *para*-5i

1H NMR (300 MHz, $(CD_3)_2SO$) δ 6.79 (d, J = 8.6 Hz, 1H, $H_{3'}$), 6.39 (d, J = 2.8 Hz, 1H, $H_{6'}$), 6.32 (dd, J = 8.6, 2.8 Hz, 1H, $H_{4'}$), 5.99 (dt, J = 7.8, 1.1 Hz, 1H H_6), 4.38 (dz, J = 7.8, 2.5 Hz, 1H, H_5), 3.60 (s, 3H, O–CH_3), 3.24 (dd, J = 18.3, 2.5 Hz, 1H, H_a of CH_2), 3.14 (dd, J = 18.3, 2.5 Hz, 1H, H_b of CH_2), 2.43 (s, 3H, N–CH_3), 1.80 (s, 15H, Cp–CH_3), 1.21 (s, 3H, C–CH_3).

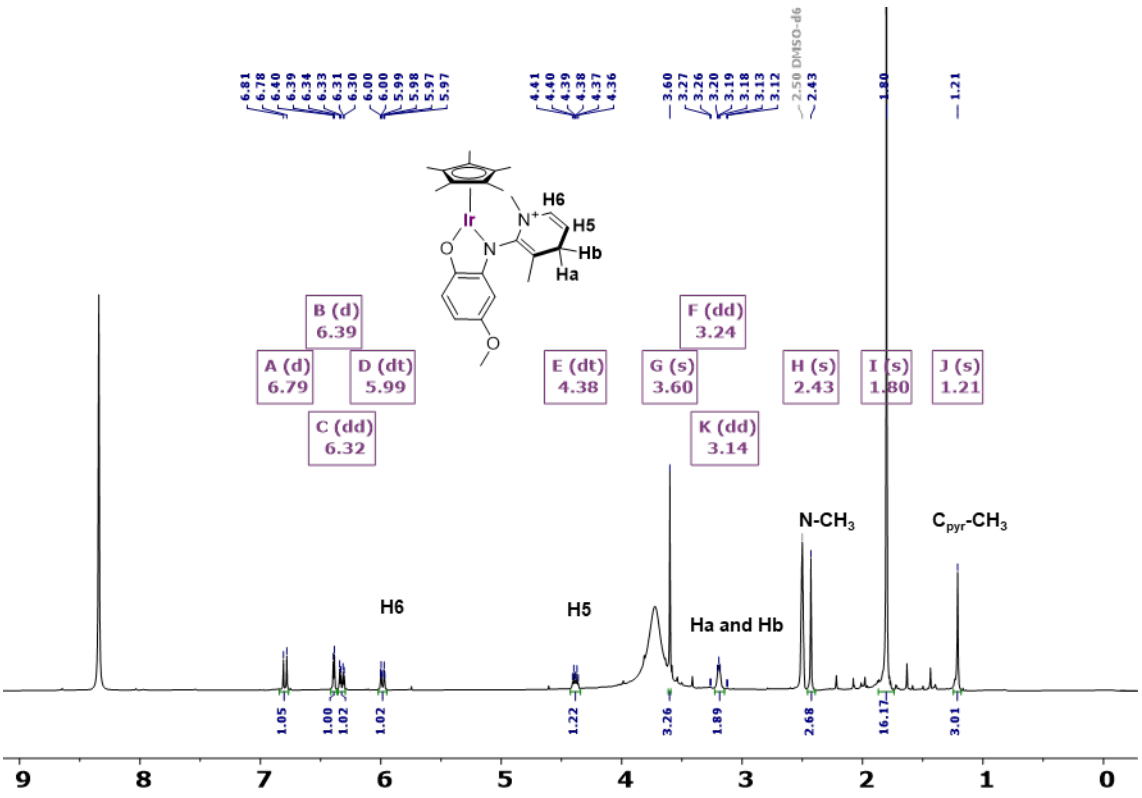

Figure S16: ^{1}H NMR spectrum (DMSO-d$_6$, 300 MHz) of ***para*-5i** with resonances for HCOOLi at 8.35 ppm and for H_2O at 3.81 ppm.

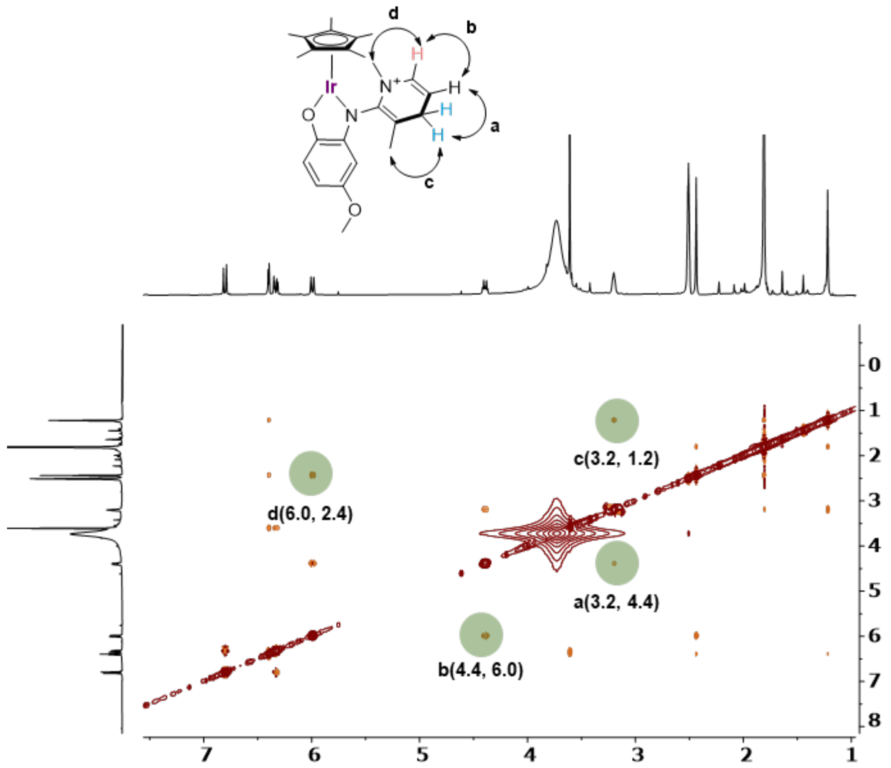

Figure S17: ^{1}H-1H NOESY NMR spectrum (DMSO-d$_6$, 300 MHz) of ***para*-5i** indicating the spatial proximity of H5 with H6 and both CH_2 protons of C4.

6 HYDRIDE STABILITY

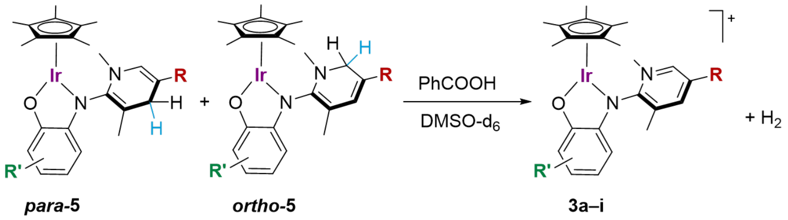

Figure S18: Protonation of the ligand-centered hydride in complexes **5a–5i** with benzoic acid (3 eq.) in DMSO-d$_6$ (0.5 mL) to yield complexes **3a–3i**.

In an NMR tube, the desired complex (0.02 mmol) was dissolved in DMSO–d$_6$ (0.30 mL). An aqueous solution of HCOOLi·H$_2$O (0.10 mL of a 20 mM solution, 0.02 mmol) was added, and the reaction mixture was kept at room temperature and monitored by ^{1}H NMR spectroscopy until full conversion (ca. 1 h), as indicated by the exclusive presence of the reduced form of the complex. Upon full conversion, benzoic acid (0.20 mL of a 0.30 M solution in DMSO–d$_6$, 0.06 mmol) was added. The reaction was monitored over time by ^{1}H NMR spectroscopy (see spectra below).

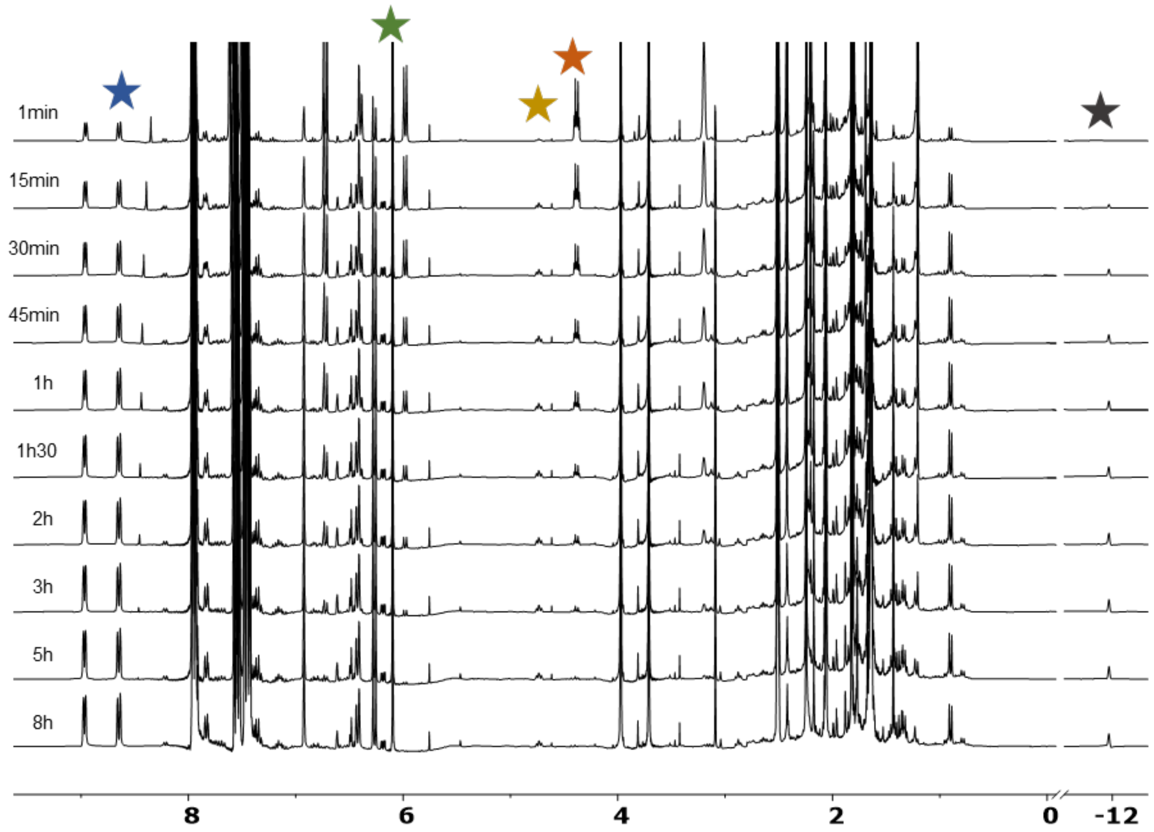

Figure S19: ^{1}H NMR spectra (300 MHz, DMSO-d$_6$) over time of complex **5a** upon addition of benzoic acid. Diagnostic signals are marked for **3a** (blue star), CH$_2$ protons of ***ortho*-5a** (yellow), CH$_2$ protons of ***para*-5a** (orange), metal hydride (black) and trimethoxybenzene as internal standard (green).

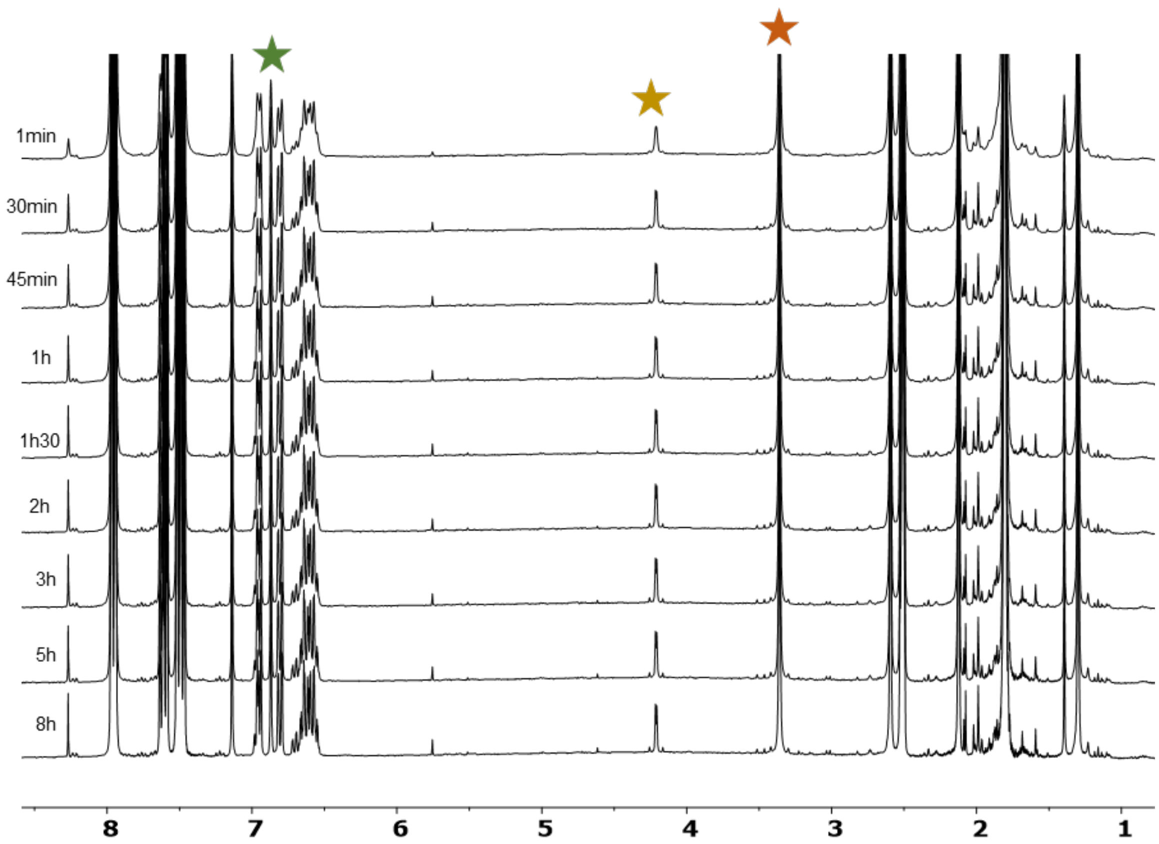

Figure S20: ^{1}H NMR spectra (300 MHz, DMSO-d$_6$) over time of complex **5b** upon addition of benzoic acid. Diagnostic signals are marked for CH_2 protons of ***ortho*-5b** (yellow), CH_2 protons of ***para*-5b** (orange), metal hydride (black) and trimethoxybenzene as internal standard (green).

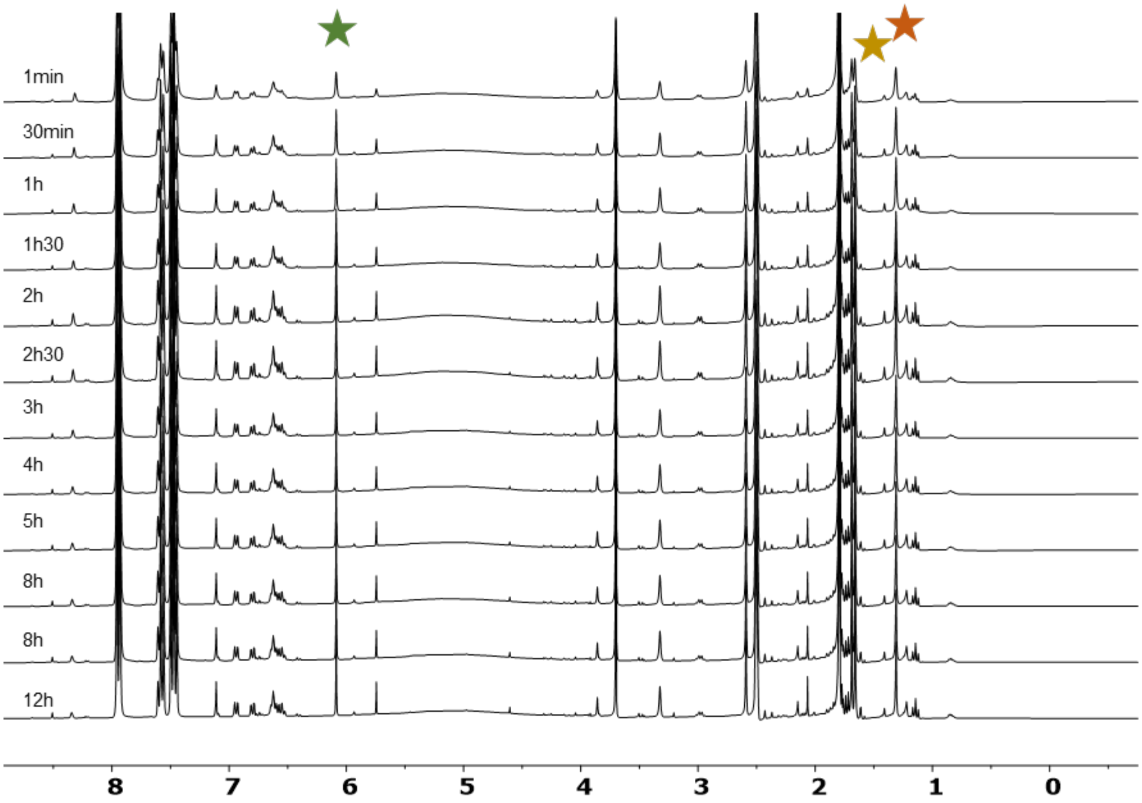

Figure S21: ^{1}H NMR spectra (300 MHz, DMSO-d$_6$) over time of complex **5c** upon addition of benzoic acid. Diagnostic signals are marked for CH_2 protons of ***ortho*-5c** (yellow), CH_2 protons of ***para*-5c** (orange), metal hydride (black) and trimethoxybenzene as internal standard (green).

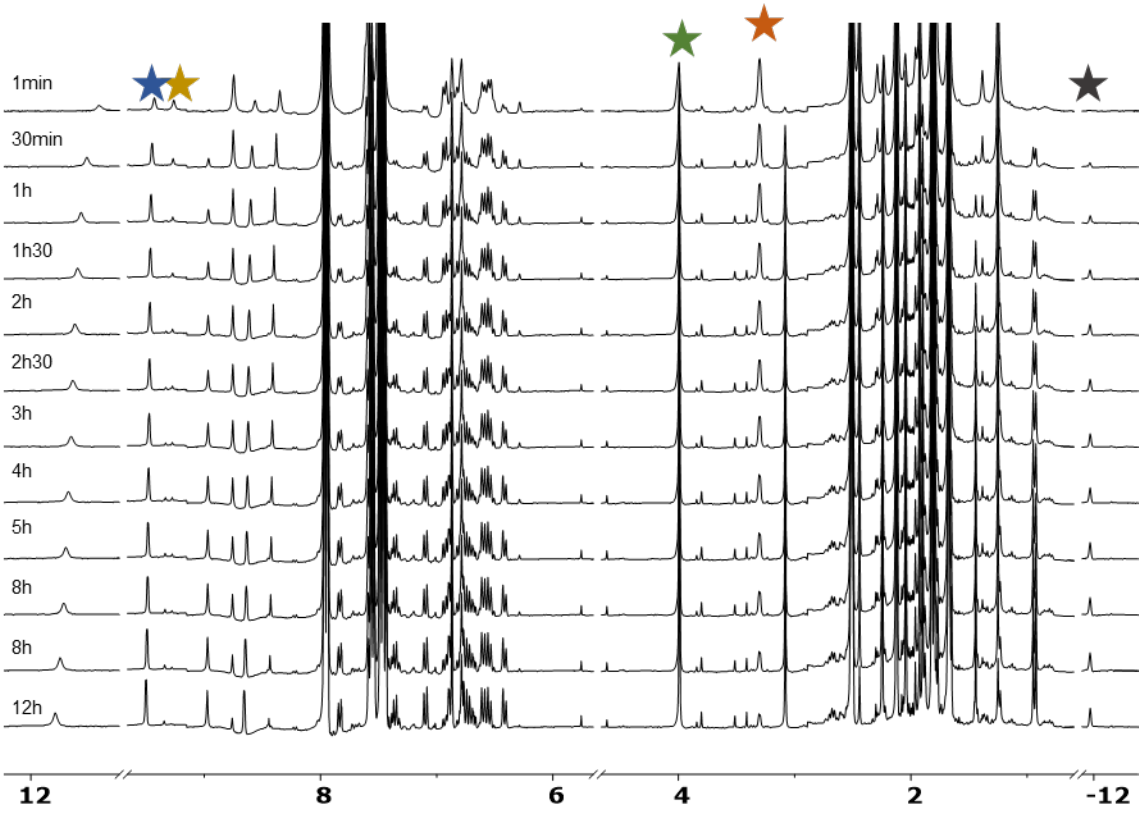

Figure S22: ^{1}H NMR spectra (300 MHz, DMSO-d$_6$) over time of complex **5d** upon addition of benzoic acid. Diagnostic signals are marked for **3d** (blue), CH_2 protons of ***ortho*-5d** (yellow), CH_2 protons of ***para*-5d** (orange), metal hydride (black) and trimethoxybenzene as internal standard (green).

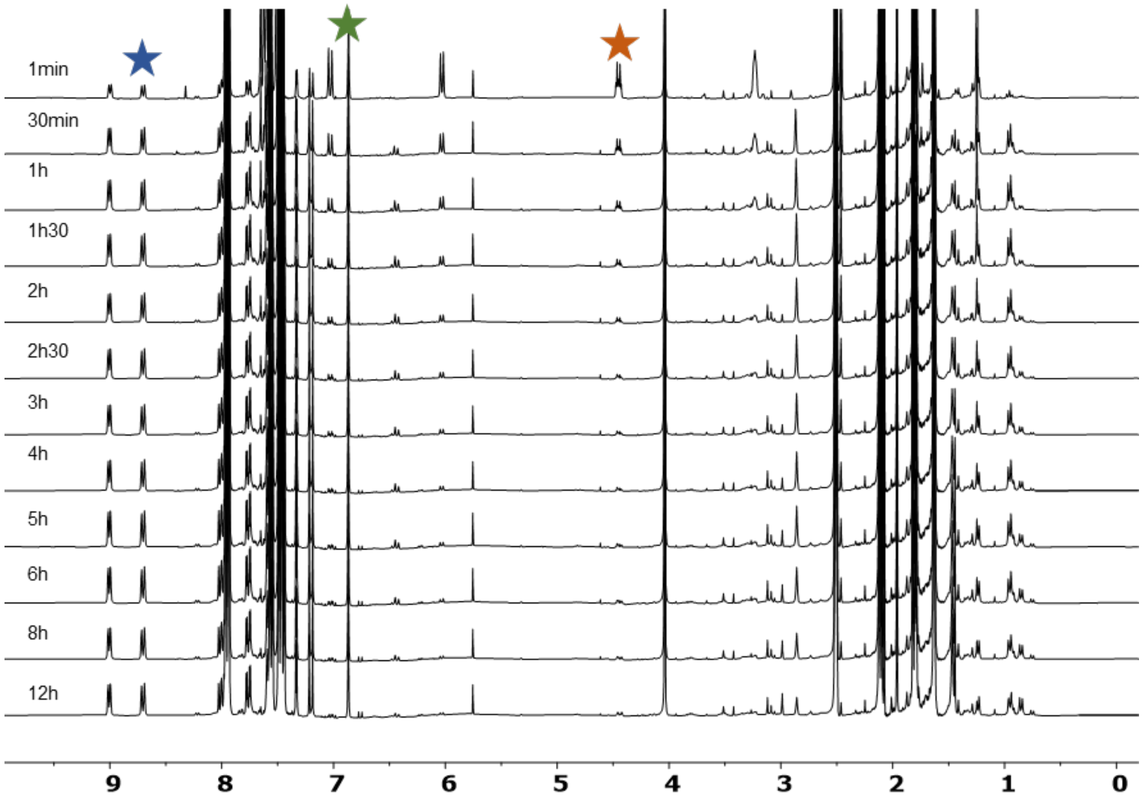

Figure S23: ^{1}H NMR spectra (300 MHz, DMSO-d_6) over time of complex **5f** upon addition of benzoic acid. Diagnostic signals are marked for **3f** (blue), CH_2 protons of ***para*-5d** (orange) and trimethoxybenzene as internal standard (green).

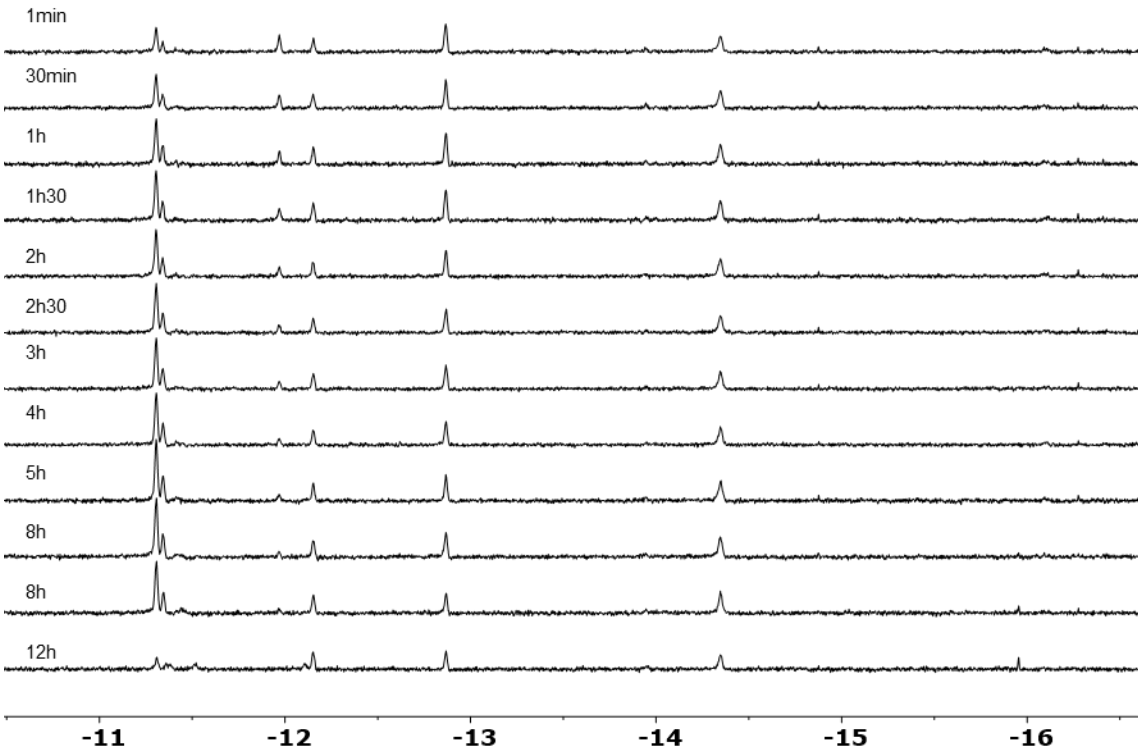

Figure S24: ^{1}H NMR spectra (300 MHz, DMSO-d_6) of the hydric region over time of complex **5f** upon addition of benzoic acid.

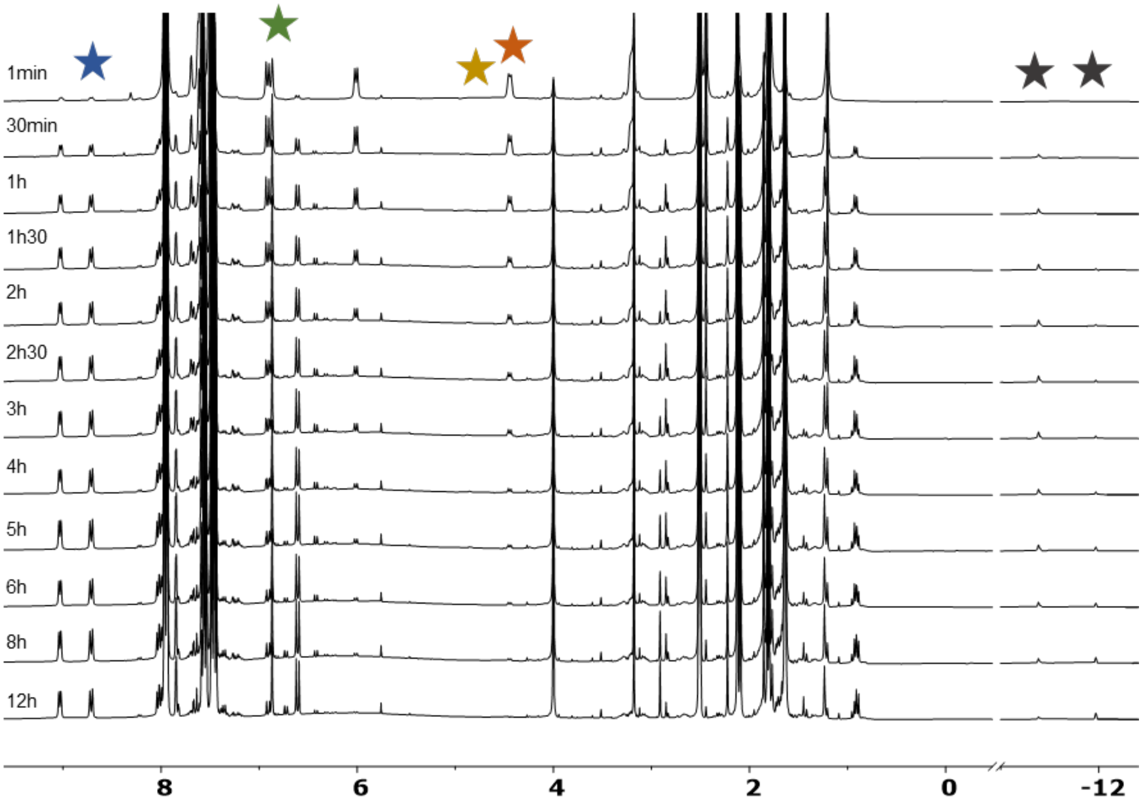

Figure S25: ^{1}H NMR spectra (300 MHz, DMSO-d$_6$) over time of complex **5g** upon addition of benzoic acid. Diagnostic signals are marked for **3g** (blue), CH_2 protons for ***ortho*-5g** (yellow), CH_2 protons of ***para*-5g** (orange), metal hydride (black) and trimethoxybenzene as internal standard (green).

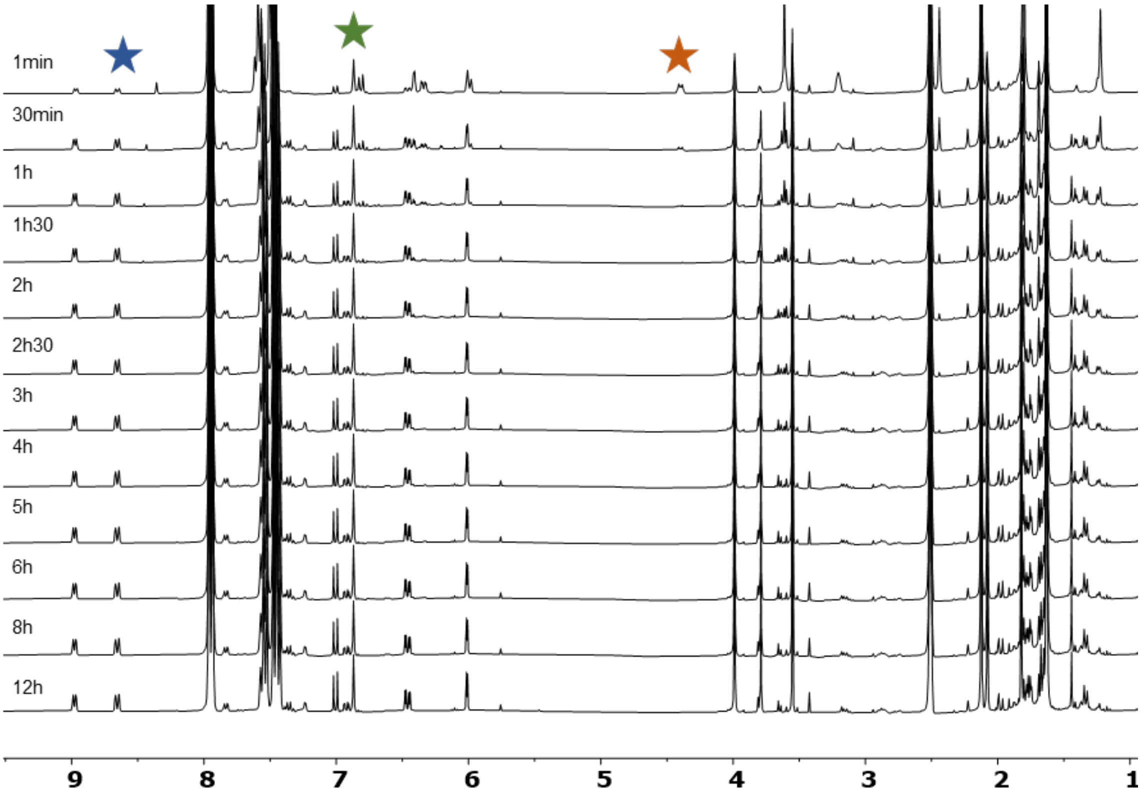

Figure S26: ^{1}H NMR spectra (300 MHz, DMSO-d$_6$) over time of complex **5h** upon addition of benzoic acid. Diagnostic signals are marked for **3h** (blue star), CH_2 protons of ***para*-5h** (orange) and trimethoxybenzene as internal standard (green).

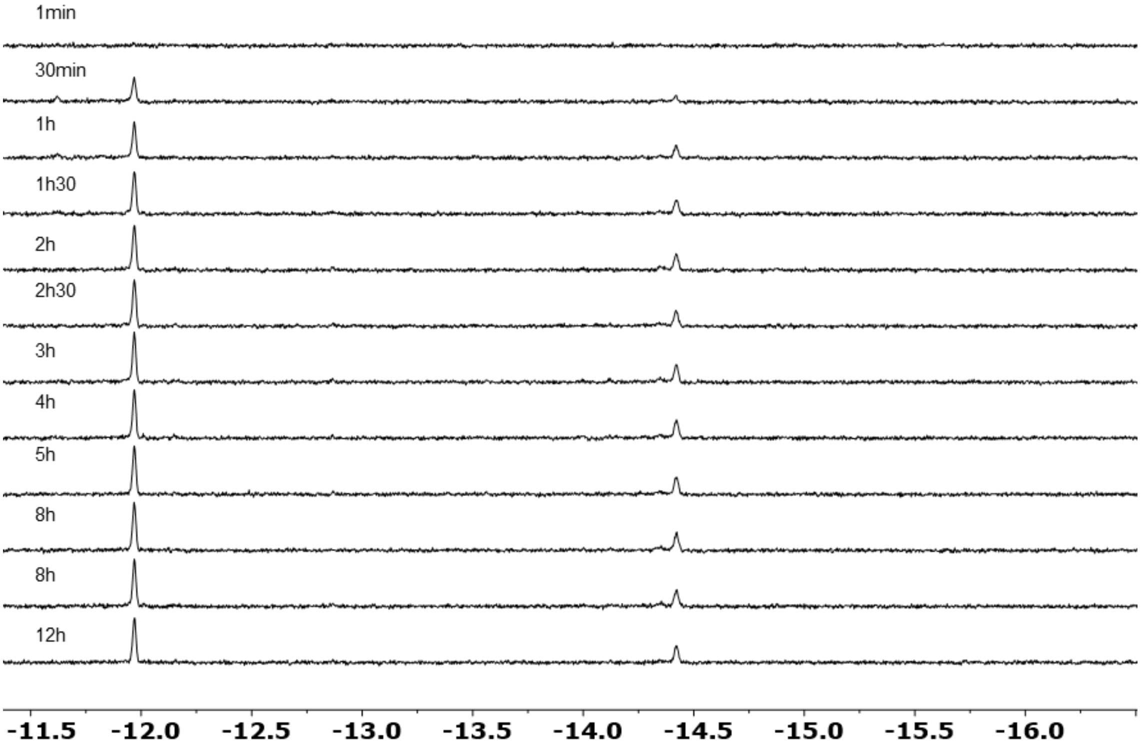

Figure S27: ^{1}H NMR spectra (300 MHz, DMSO-d_6) of the hydric region over time of complex **5h** upon addition of benzoic acid.

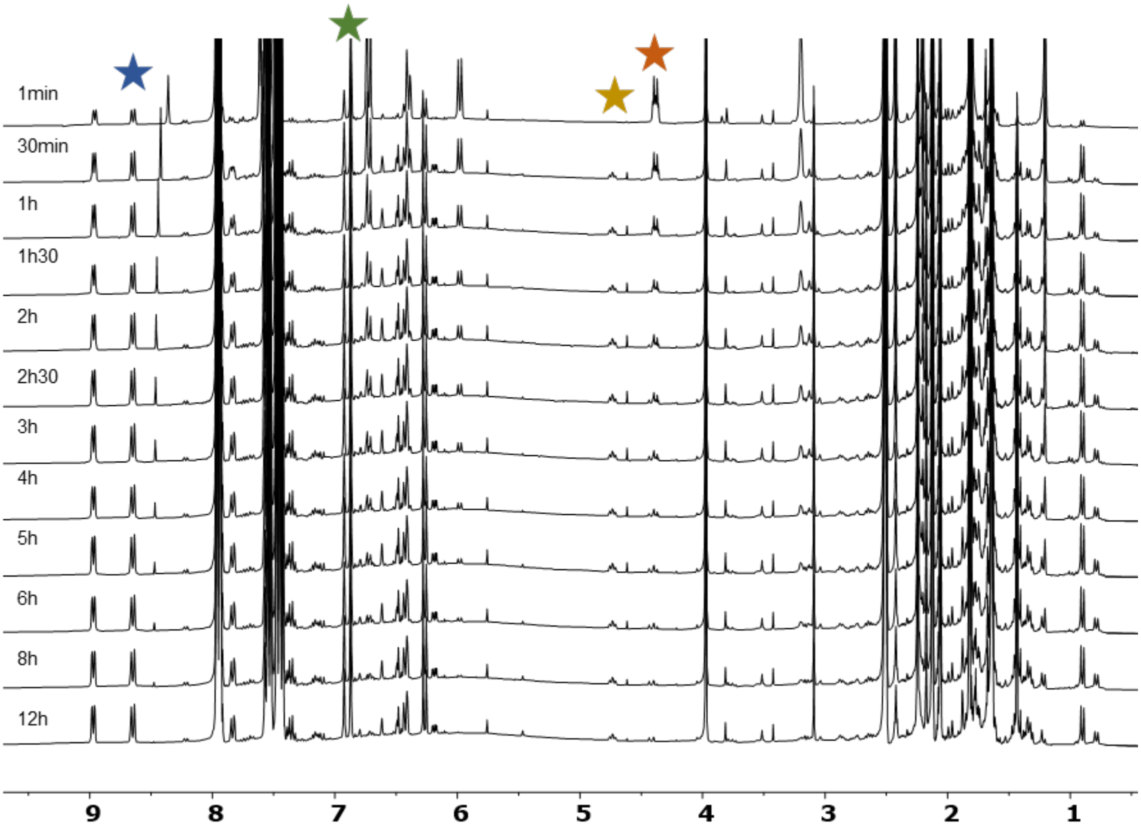

Figure S28: ^{1}H NMR spectra (300 MHz, DMSO-d_6) over time of complex **5i** upon addition of benzoic acid. Diagnostic signals are marked for **3i** (blue star), CH_2 protons of ***ortho*-5i** (yellow), CH_2 protons of ***para*-5i** (orange), metal hydride (black) and trimethoxybenzene as internal standard (green).

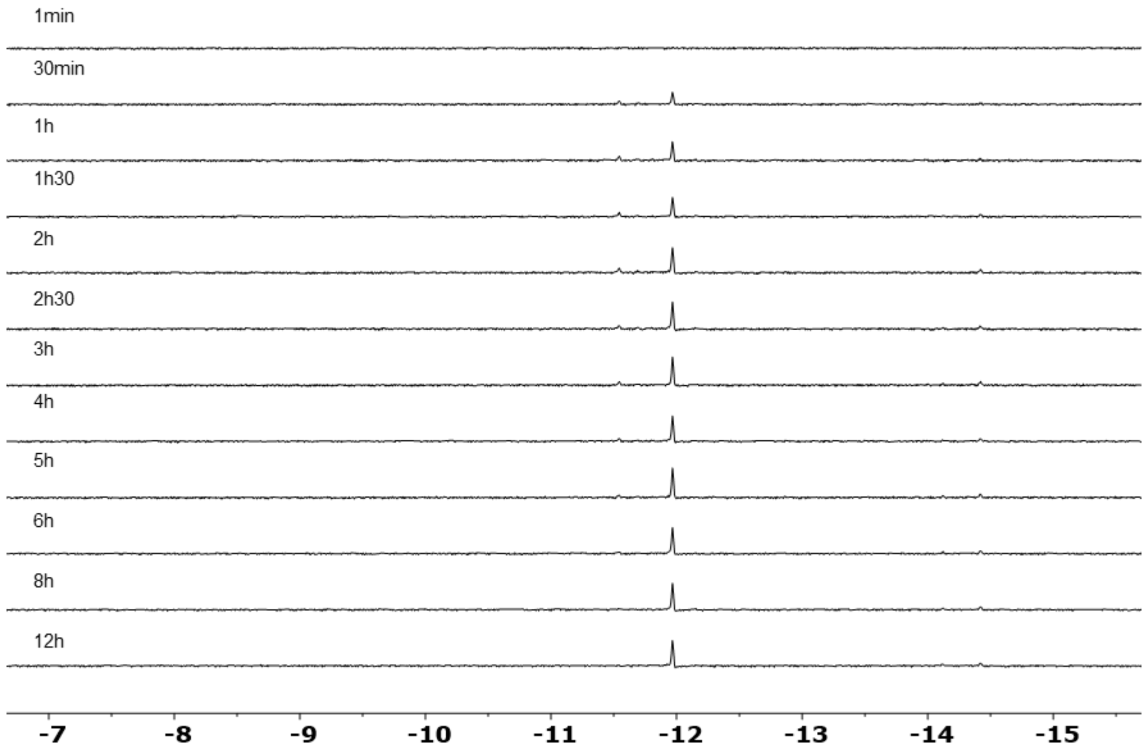

Figure S29: ^{1}H NMR spectra (300 MHz, DMSO-d$_6$) of the hydric region over time of complex **5i** upon addition of benzoic acid.

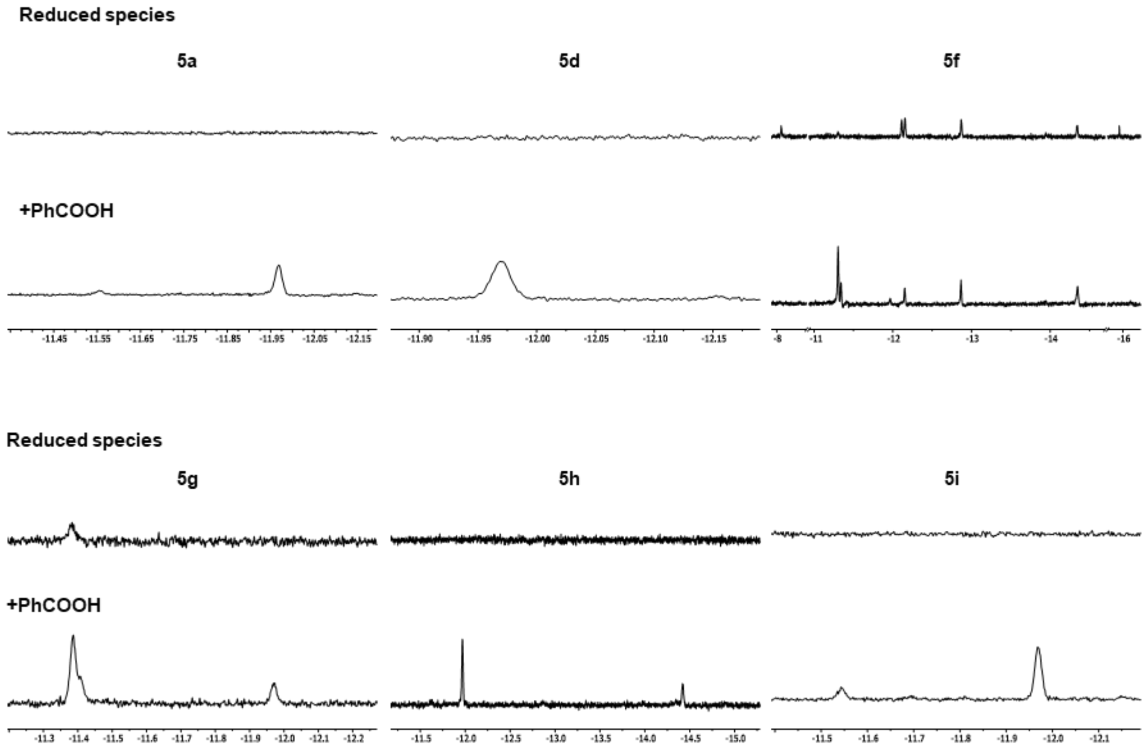

Figure S30: Comparison of ^{1}H NMR spectra (300 MHz, DMSO-d_6) zoomed to the metal hydride region before and after 1 hour from benzoic acid addition in all complexes forming detectable metal hydrides (**5a**, **5d**, **5f**, **5g**, **5h** and **5i**).

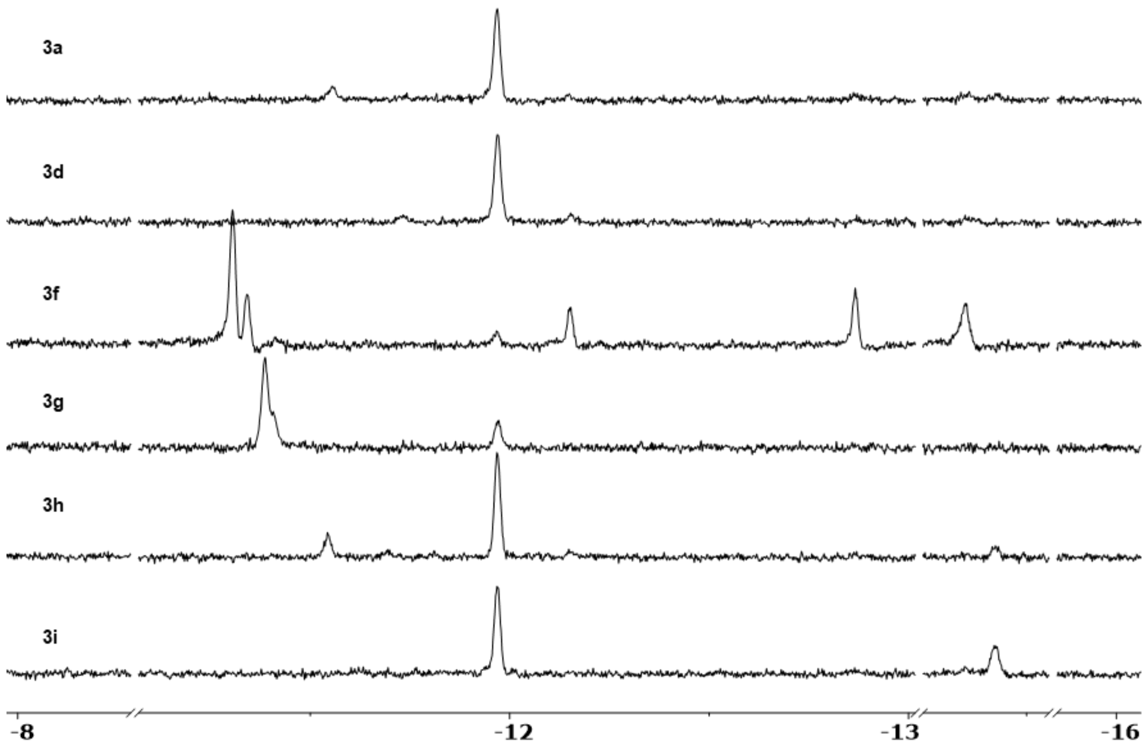

Figure S31:Comparison of metal hydrides observed in the negative ^{1}H NMR region 1 hour after addition of benzoic acid on the reduced complex species.

7 CATALYTIC DATA

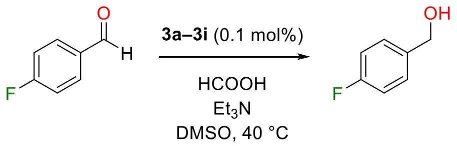

To a solution of the iridium complex **3** (1.0 mM in DMSO–d_6, 0.1 mL, 0.1 mmol) was added durene (13.4 mg, 0.1 mmol), DMSO-d_6 (0.4 mL), HCOOH (45 µL, 1.2 mmol), and Et_3N (210 µL, 1.5 mmol). Then, 4-F-benzaldehyde (105 µL, 1.0 mmol) was added, and the NMR tube was placed in an oil bath at 40° C. Yields of the alcohol and conversion of the carbonyl substrate were determined over time by integrating the signals at 9.98 ppm (starting material), 4.50 ppm (product), and 2.07 ppm (internal standard) by ^{1}H NMR spectroscopy.

The catalytic rate constants were determined assuming pseudo-first-order kinetics with respect to the substrate. The conversion was monitored over time, and the concentration of product formed, $[P]$, was used to calculate the substrate concentration $[S]$ according to:

$$\ln[S] = \ln(1 - [P]) = -k_{\mathrm{obs}} \cdot t$$

Linear regression of $\ln(1 - [P])$versus time afforded the observed rate constant $k_{\mathrm{obs}}$from the slope.

Table S2: ln[S]= ln(1-[P]) over time for complex **3a- i**

Time (h)	ln(1-[P])								
	**3a**	**3b**	**3c**	**3d**	**3e**	**3f**	**3g**	**3h**	**3i**
0	0.00	0.00	0.00	0.00	0	0.00	0.00	0.00	0.00
0.25	-	-0.06	-1.17	-0.25	0	-0.42	-0.27	-1.19	-0.29
0.5	-0.89	-0.14	-1.90	-0.85	0	-0.61	-0.36	-3.78	-1.07
0.75	-	-0.16	-2.28	-1.55	0	-0.75	-0.47	-	-3.01
1	-2.84	-0.18	-2.52	-2.31	0	-0.92	-0.55	-	-
1.5	-	-0.21	-2.91	-3.21	0	-1.23	-0.82	-	-
2	-	-0.25	-3.26	-	0	-1.54	-1.14	-	-
slope	-2.63	-0.15	-3.98	-3.16	n.d.	-0.84	-0.57	-7.01	-3.28
R^2	0.975	0.944	0.992	0.996	n.d.	0.979	0.991	0.975	0.912

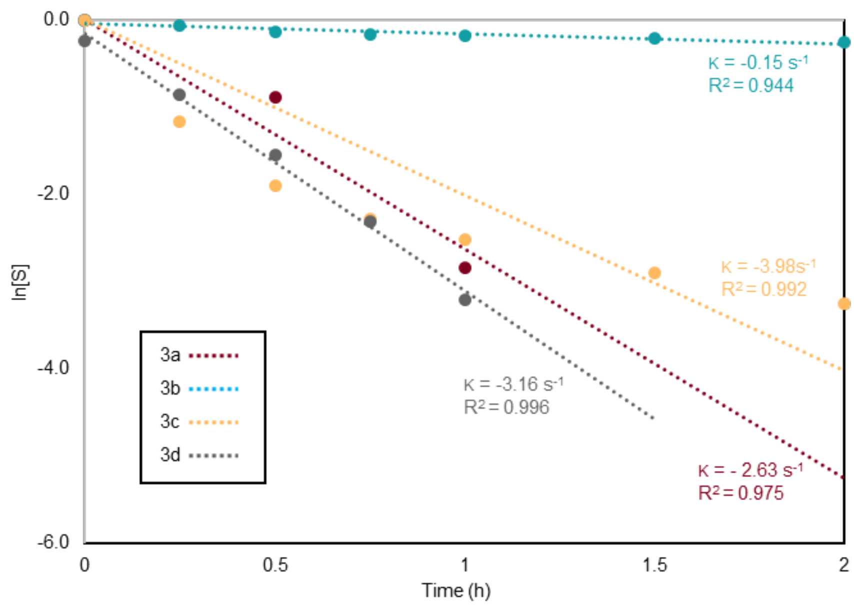

Figure S32: Plot of ln[S] over time for complex **3a- d**

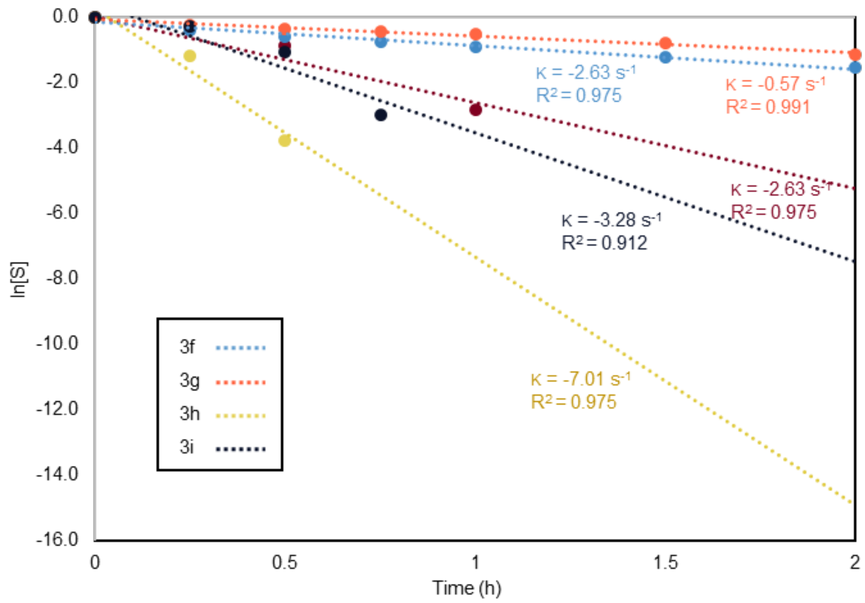

Figure S33: Plot of ln[S] over time for complex **3a** and **3f-i**

8 NMR SPECTRA OF NEW COMPOUNDS

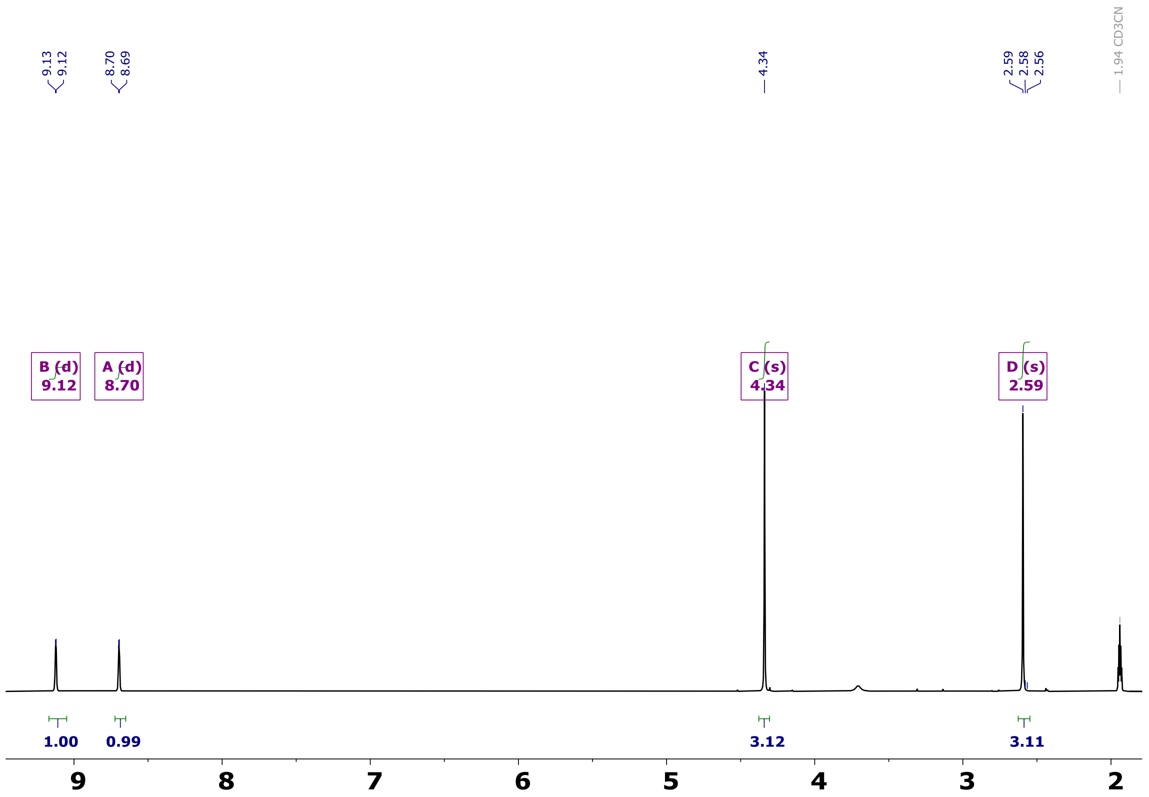

Figure S34: ^{1}H NMR spectrum (CD_3CN, 400 MHz) of **1b**.

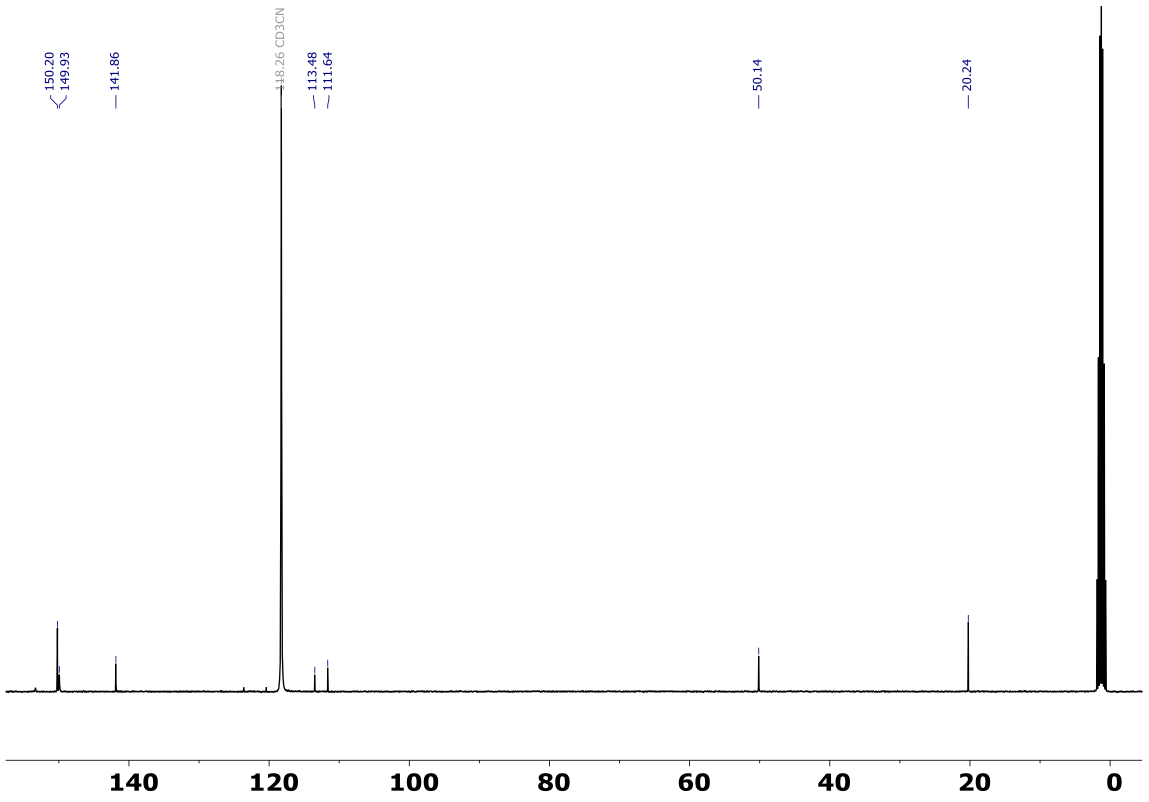

Figure S35: $^{13}C\{^1H\}$ NMR spectrum (CD_3CN, 101 MHz) of **1b**.

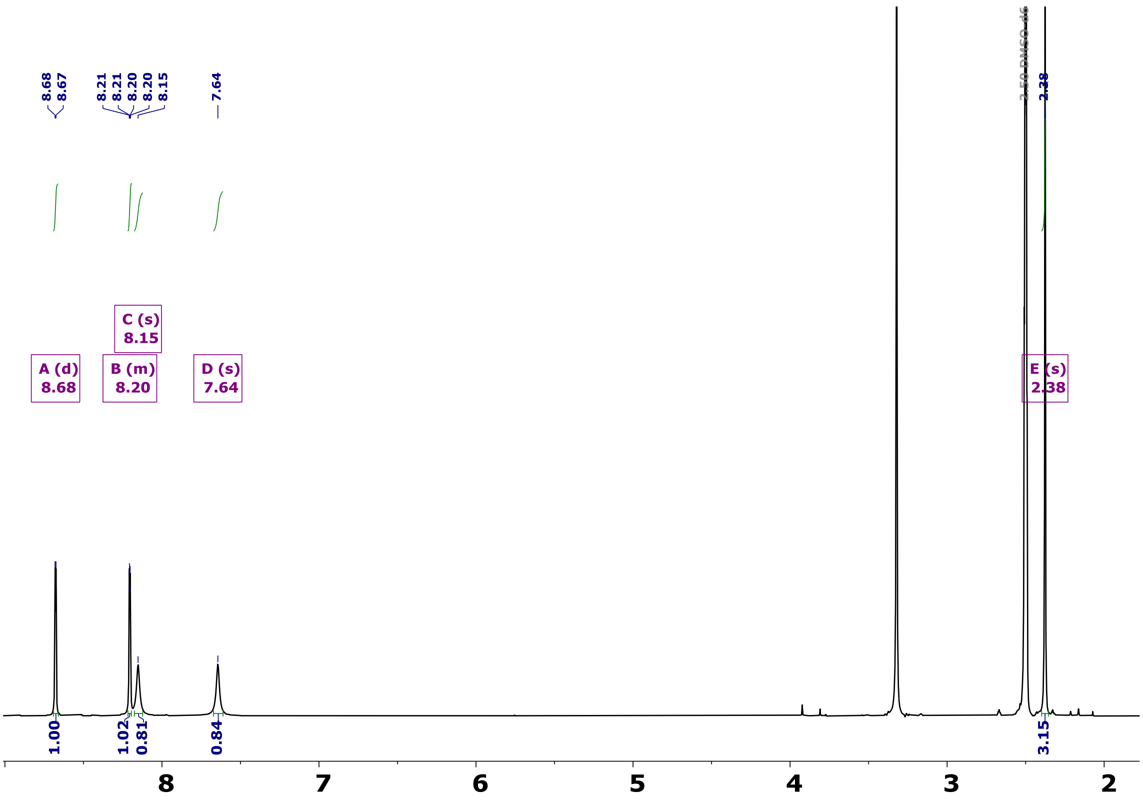

Figure S36: ^{1}H NMR spectrum (DMSO-d$_6$, 400 MHz) of **6-chloro-5-methylnicotinamide**.

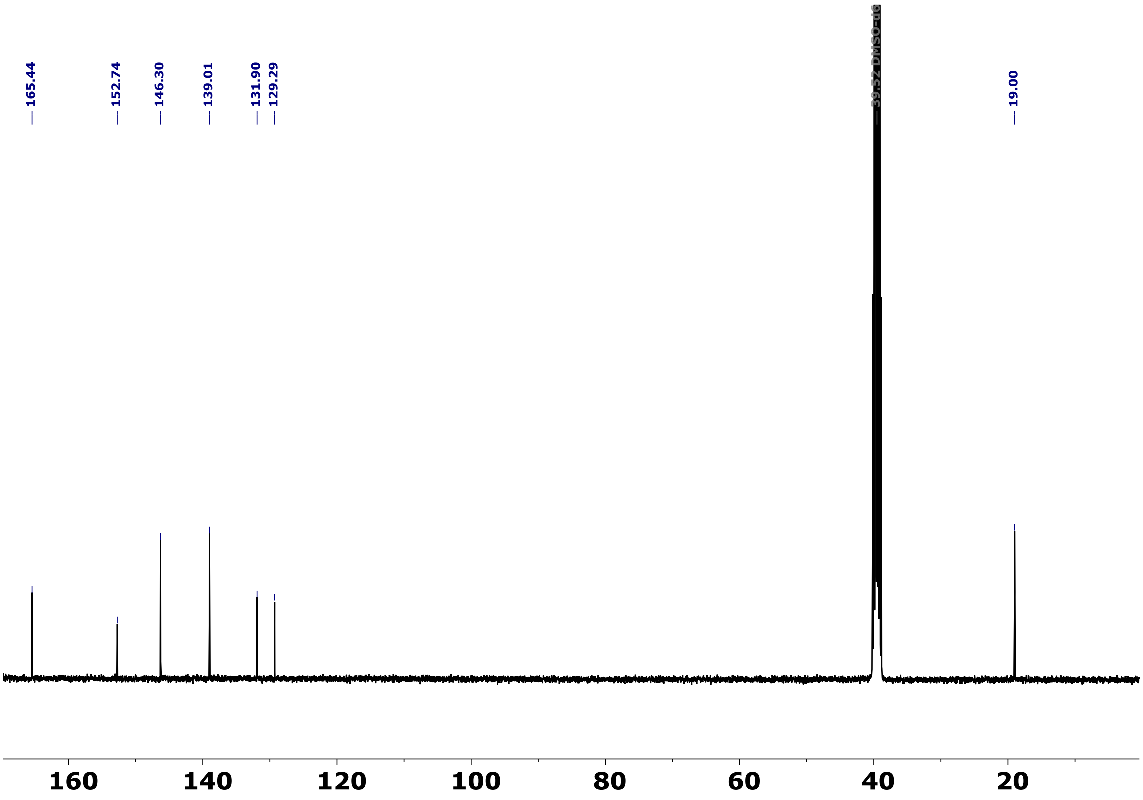

Figure S37: $^{13}C\{^{1}H\}$ NMR spectrum (DMSO-d$_6$, 101 MHz) of **6-chloro-5-methylnicotinamide**.

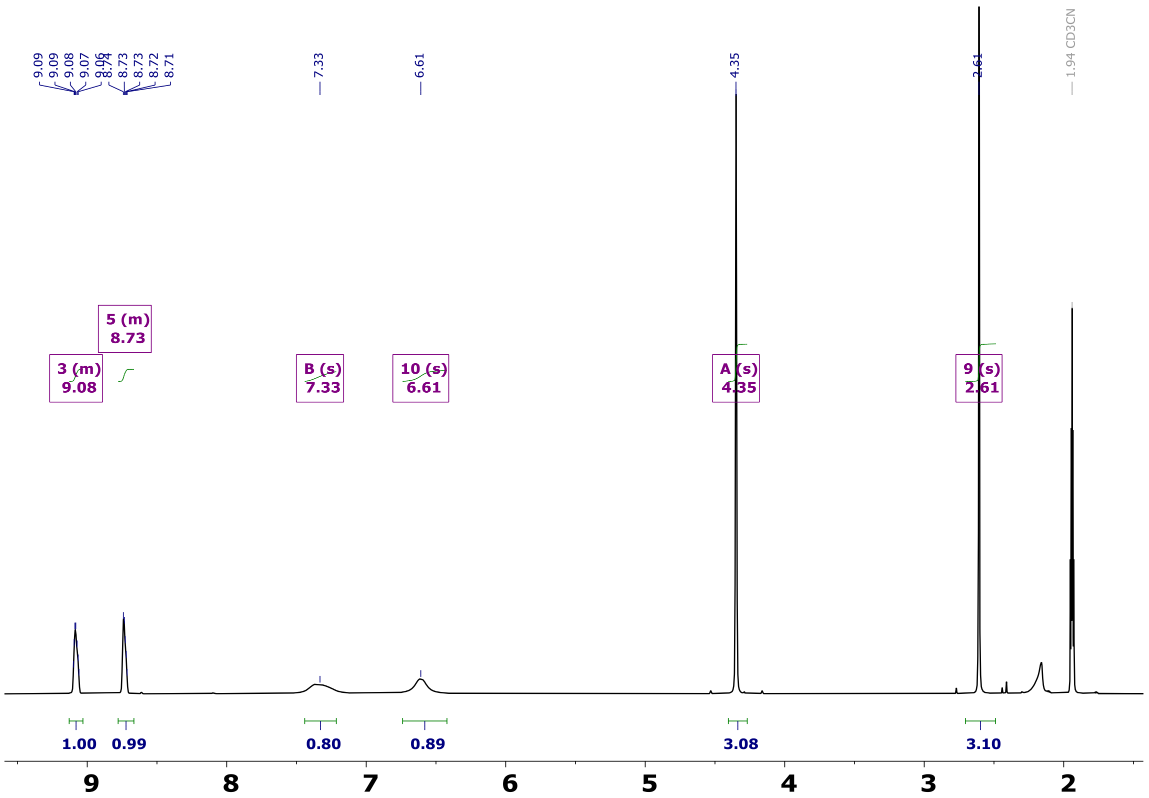

Figure S38: ^{1}H NMR spectrum (CD_3CN, 400 MHz) of **1c**.

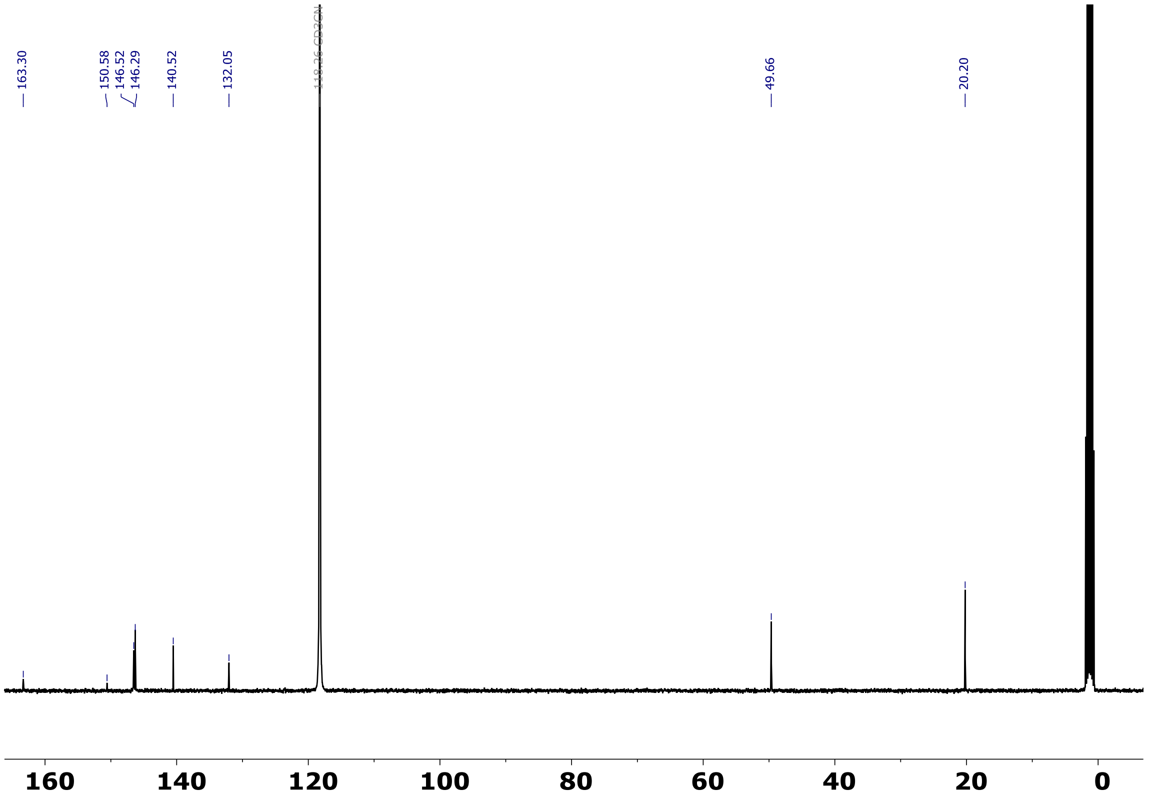

Figure S39: $^{13}C\{^{1}H\}$ NMR spectrum (CD_3CN, 101 MHz) of **1c**.

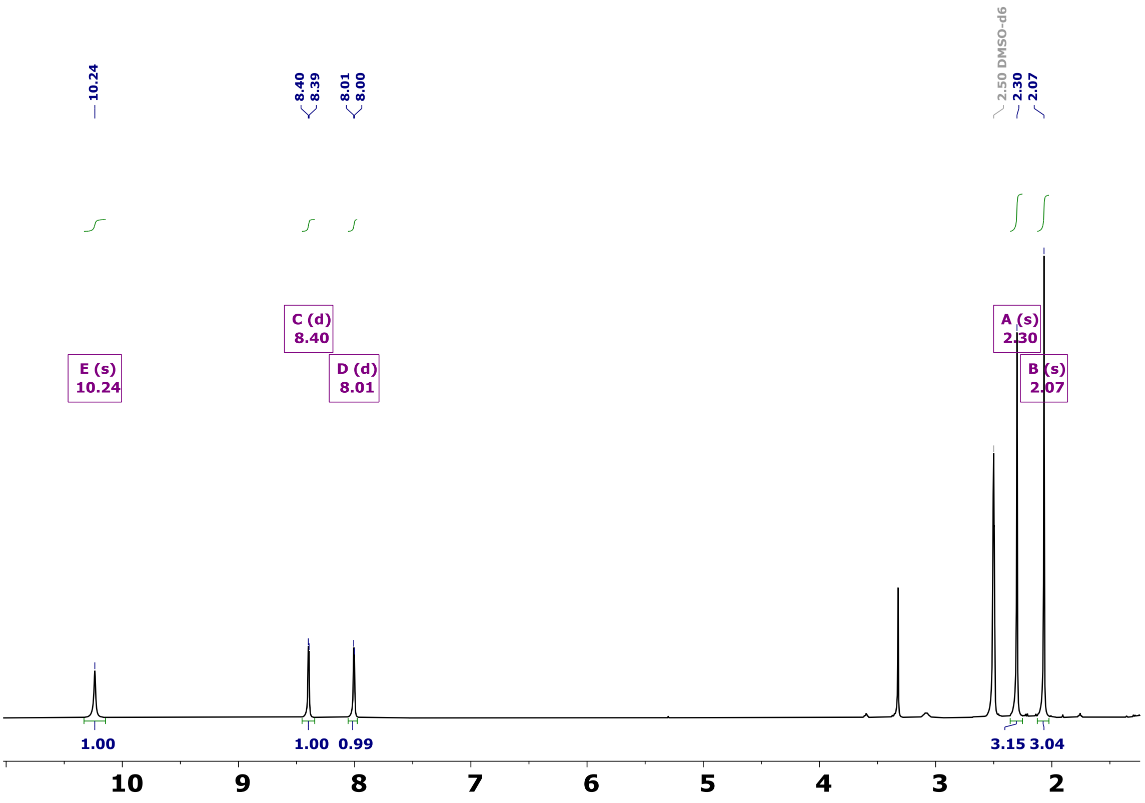

Figure S40: ^{1}H NMR spectrum (DMSO-d$_6$, 400 MHz) of **N-(6-chloro-5-methylpyridin-3-yl)acetamide**.

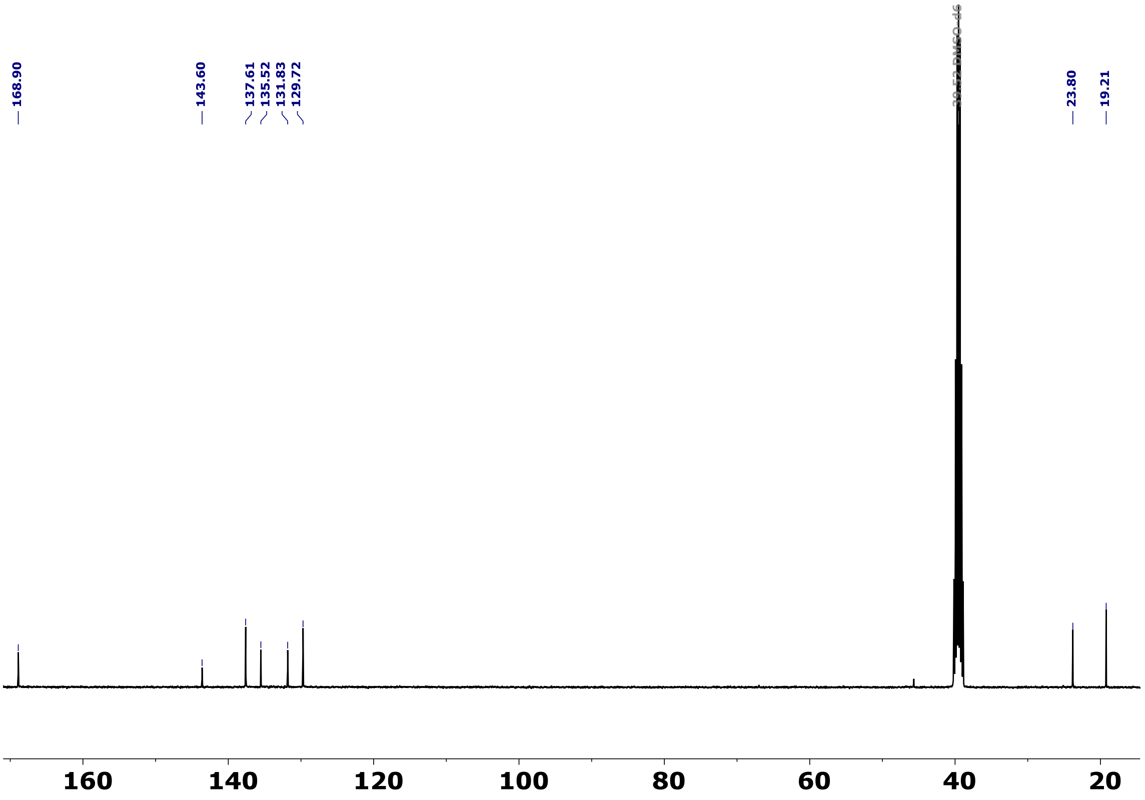

Figure S41: ^{13}C{^{1}H} NMR spectrum (DMSO-d$_6$, 101 MHz) of **N-(6-chloro-5-methylpyridin-3-yl)acetamide**.

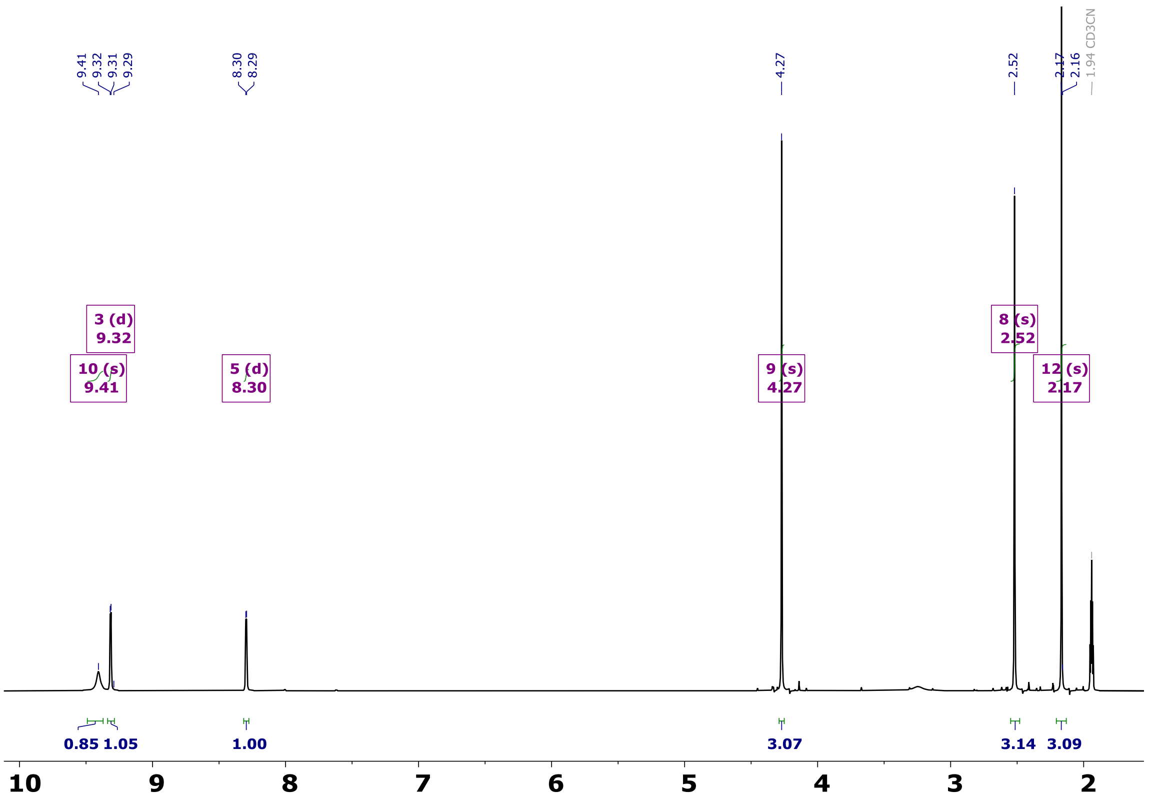

Figure S42: ^{1}H NMR spectrum (CD_3CN, 400 MHz) of **1d**.

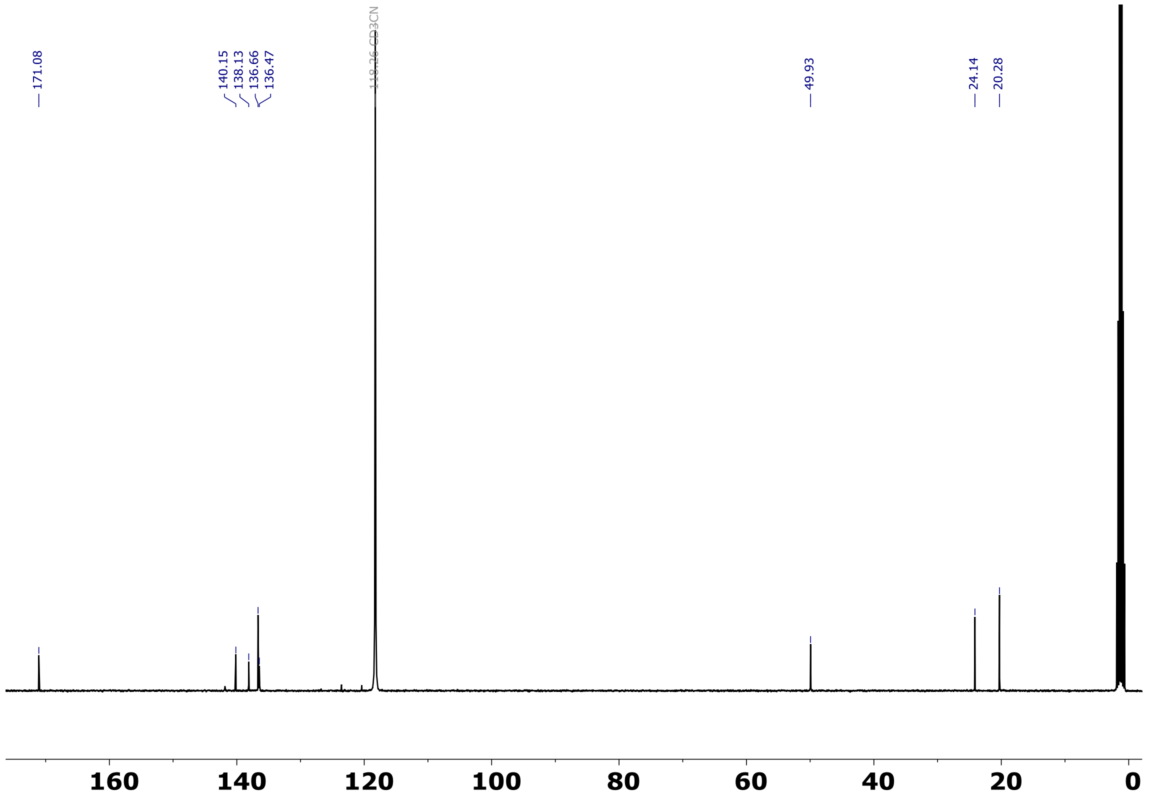

Figure S43: $^{13}C\{^{1}H\}$ NMR spectrum (CD_3CN, 101 MHz) of **1d**.

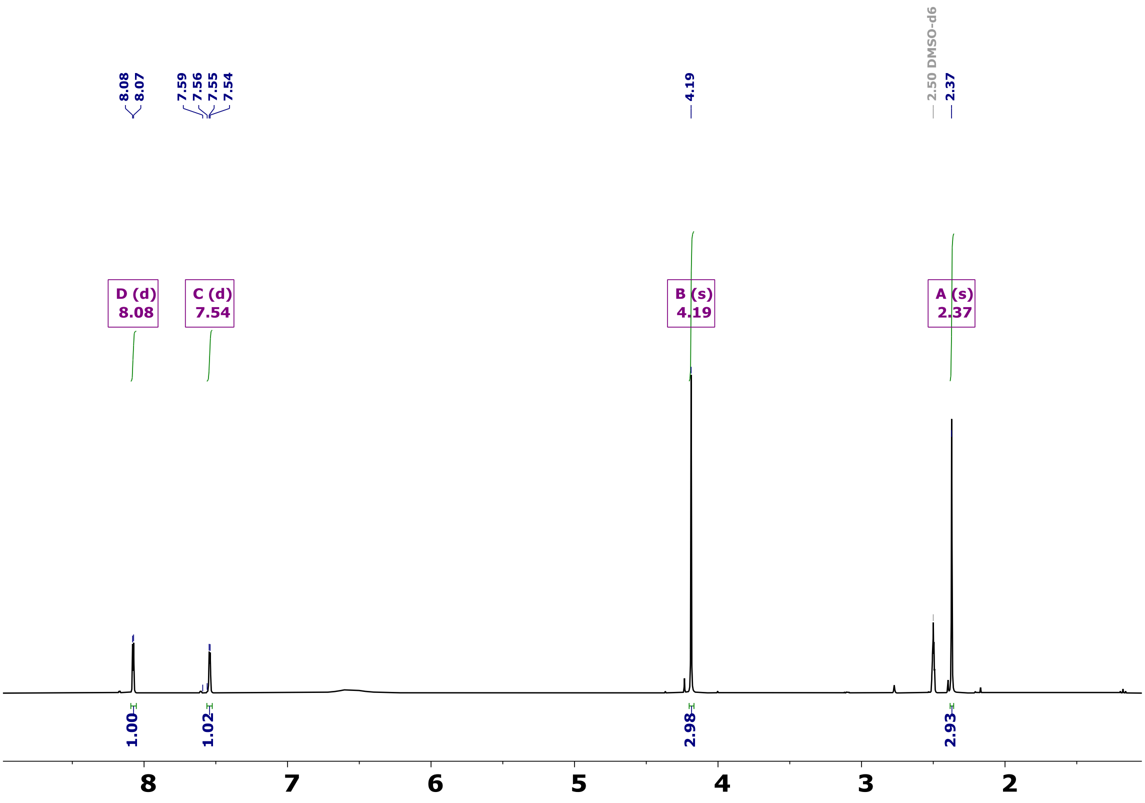

Figure S44: ^{1}H NMR spectrum (DMSO-d_6, 400 MHz) of **1e**.

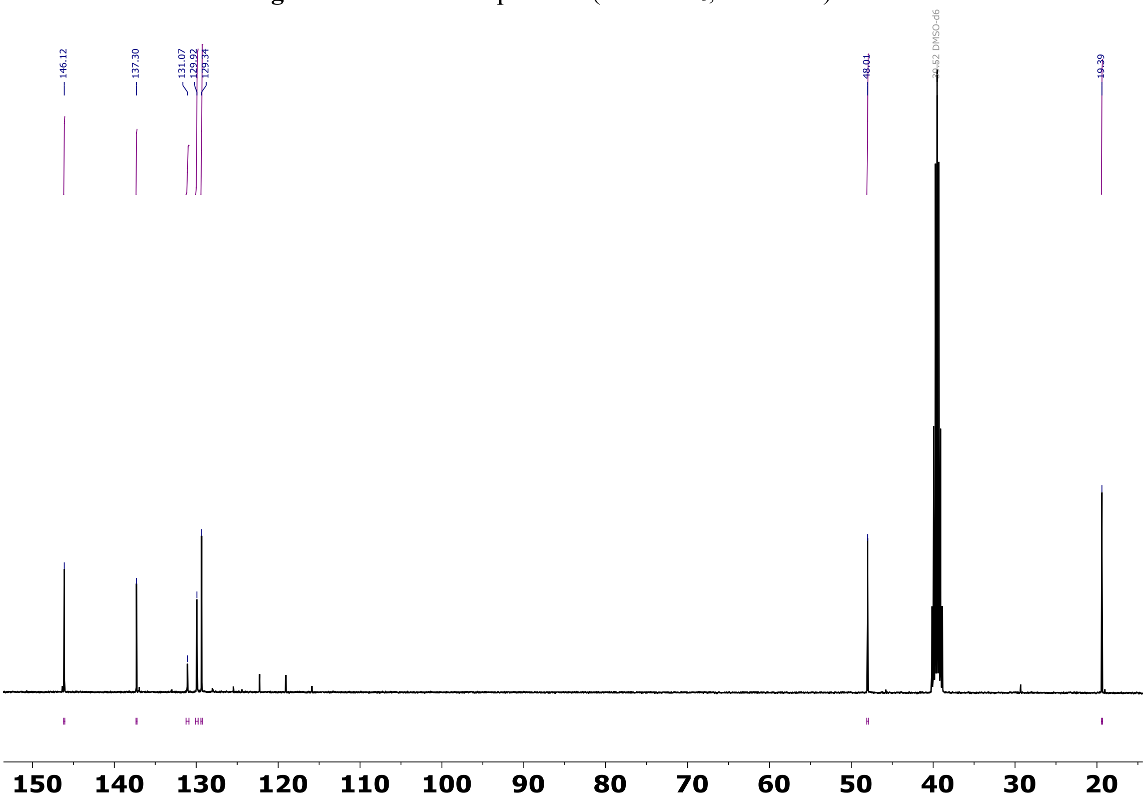

Figure S45: $^{13}C\{^{1}H\}$ NMR spectrum (DMSO-d_6,101 MHz) of **1e**.

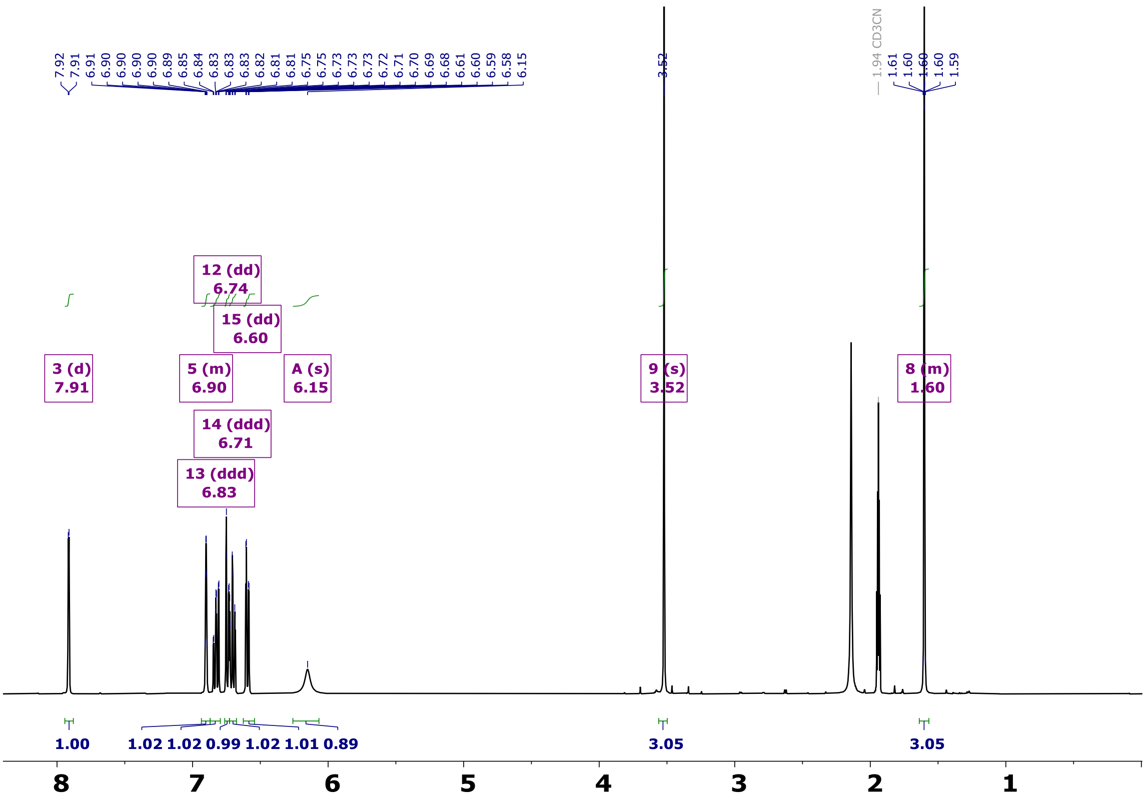

Figure S46: ^{1}H NMR spectrum (CD_3CN, 400 MHz) of **2b**.

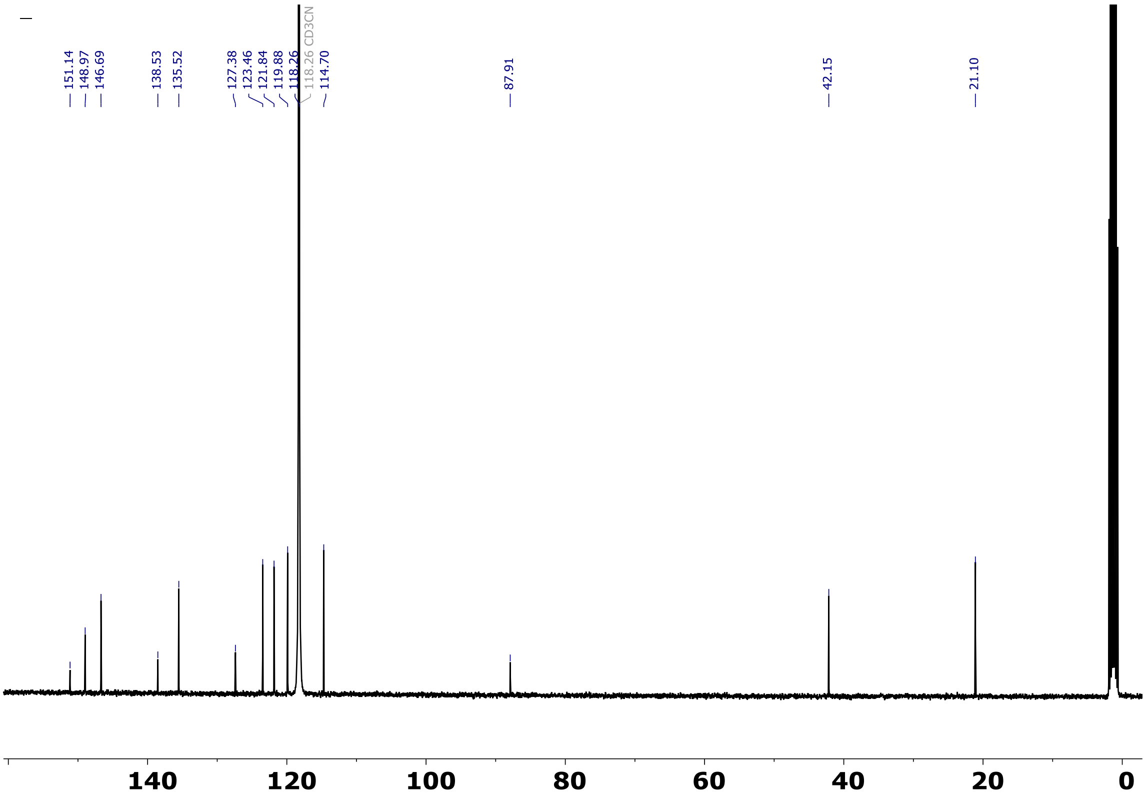

Figure S47: $^{13}C\{^{1}H\}$ NMR spectrum (CD_3CN, 101 MHz) of **2b**.

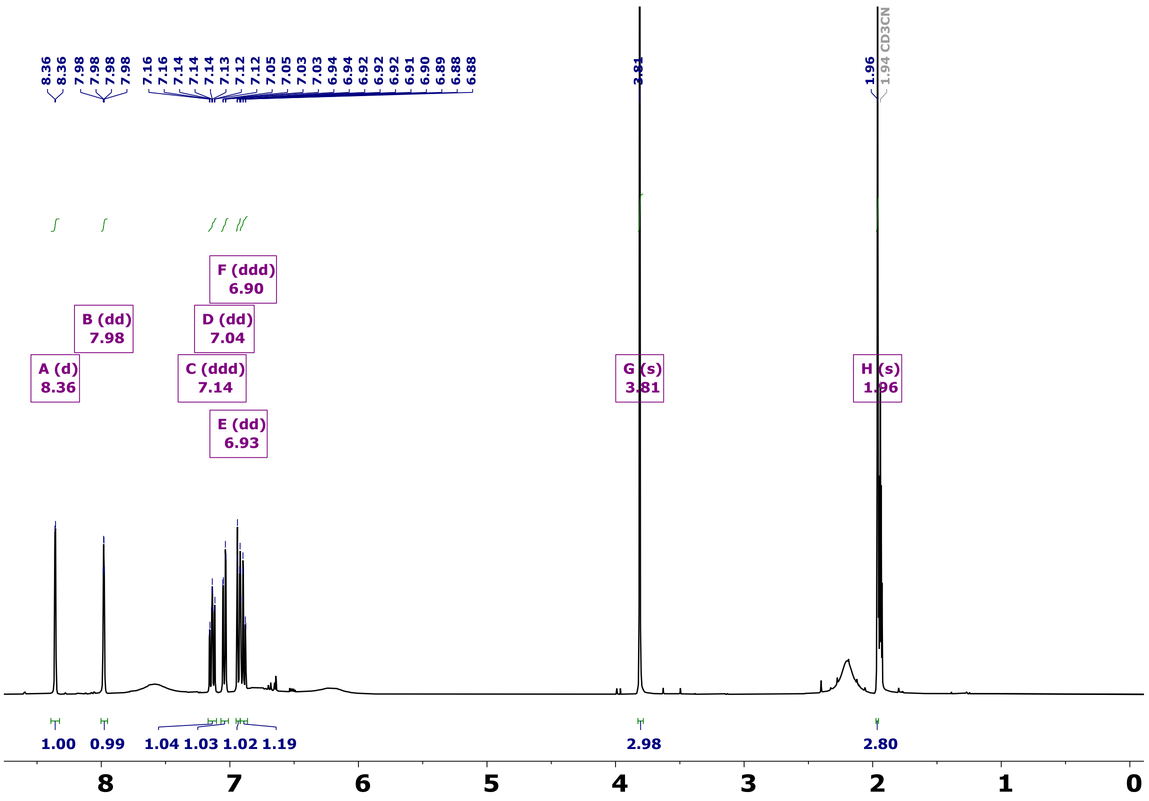

Figure S48: ^{1}H NMR spectrum (CD_3CN, 400 MHz) of **2c**.

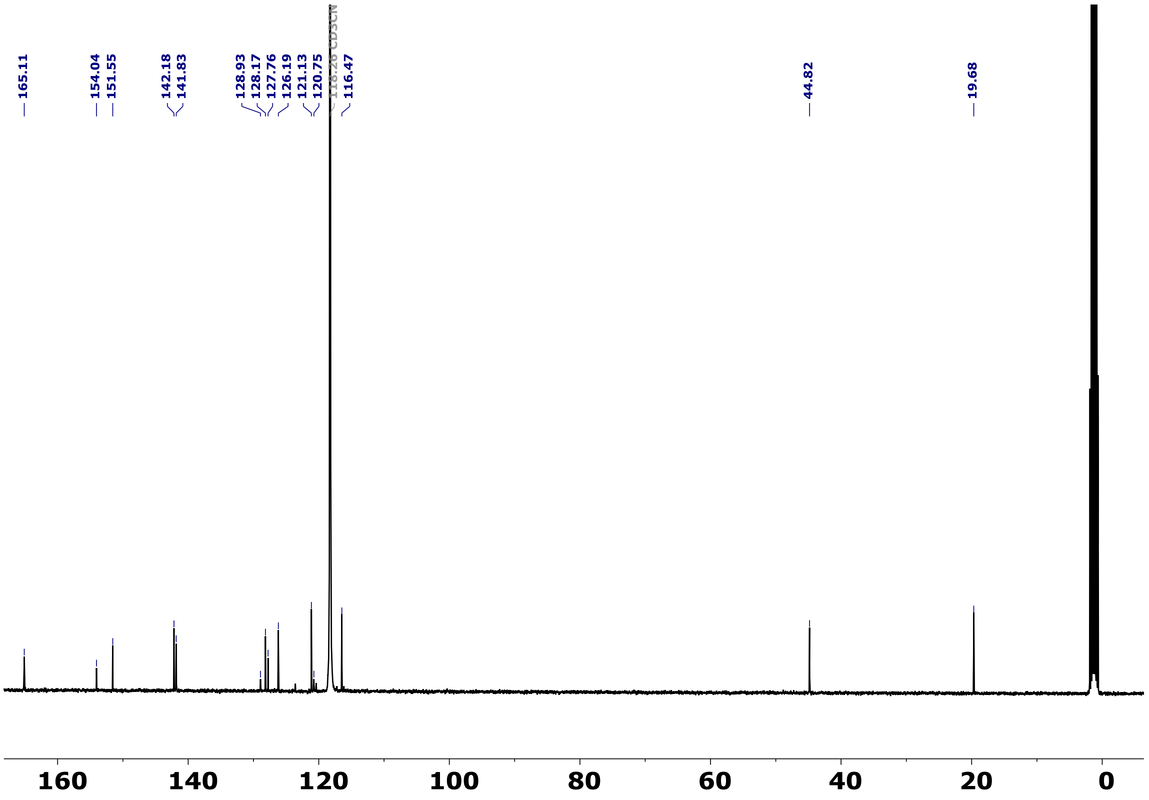

Figure S49: $^{13}C\{^{1}H\}$ NMR spectrum (CD_3CN, 101 MHz) of **2c**.

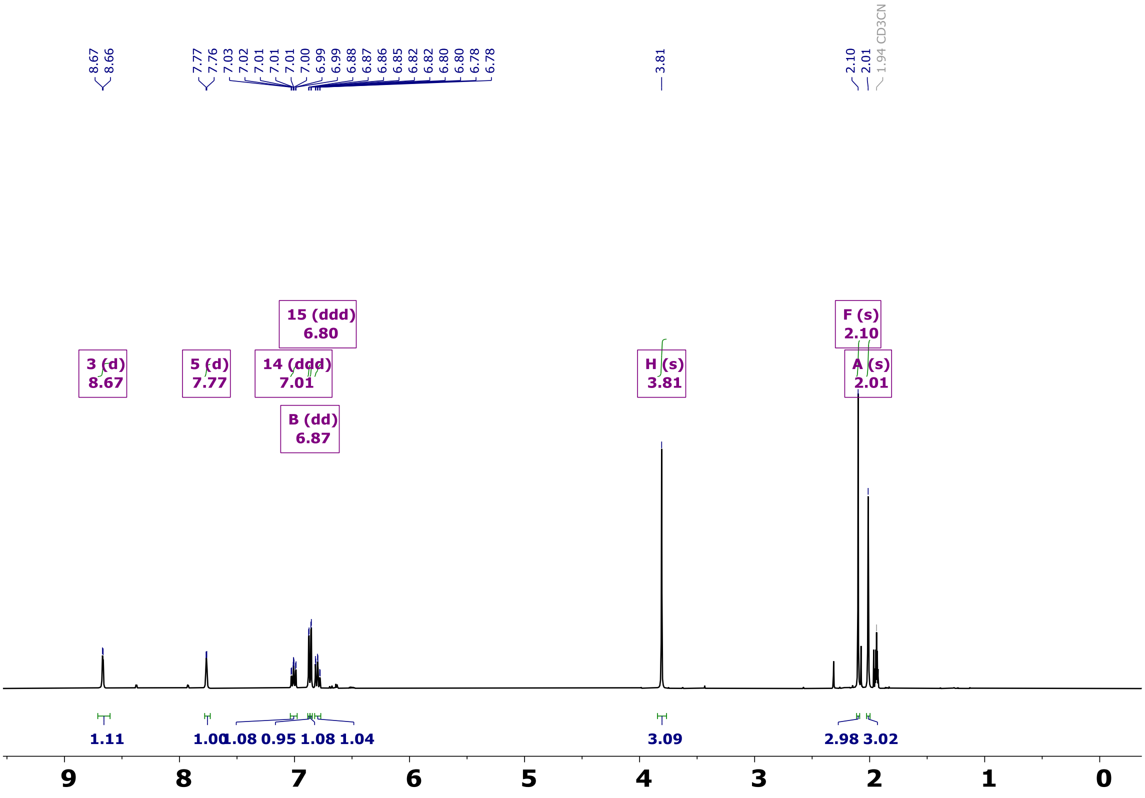

Figure S50: ^{1}H NMR spectrum (CD_3CN, 400 MHz) of **2d**.

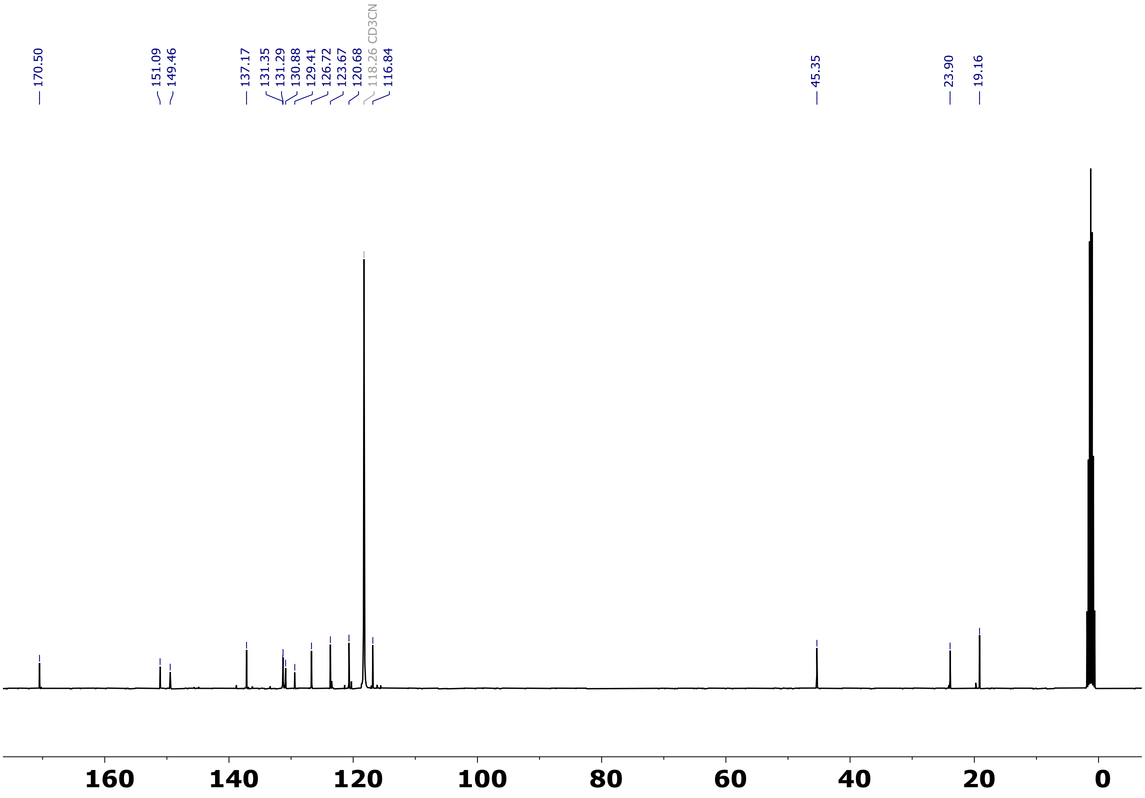

Figure S51: $^{13}C\{^{1}H\}$ NMR spectrum (CD_3CN, 101 MHz) of **2d**.

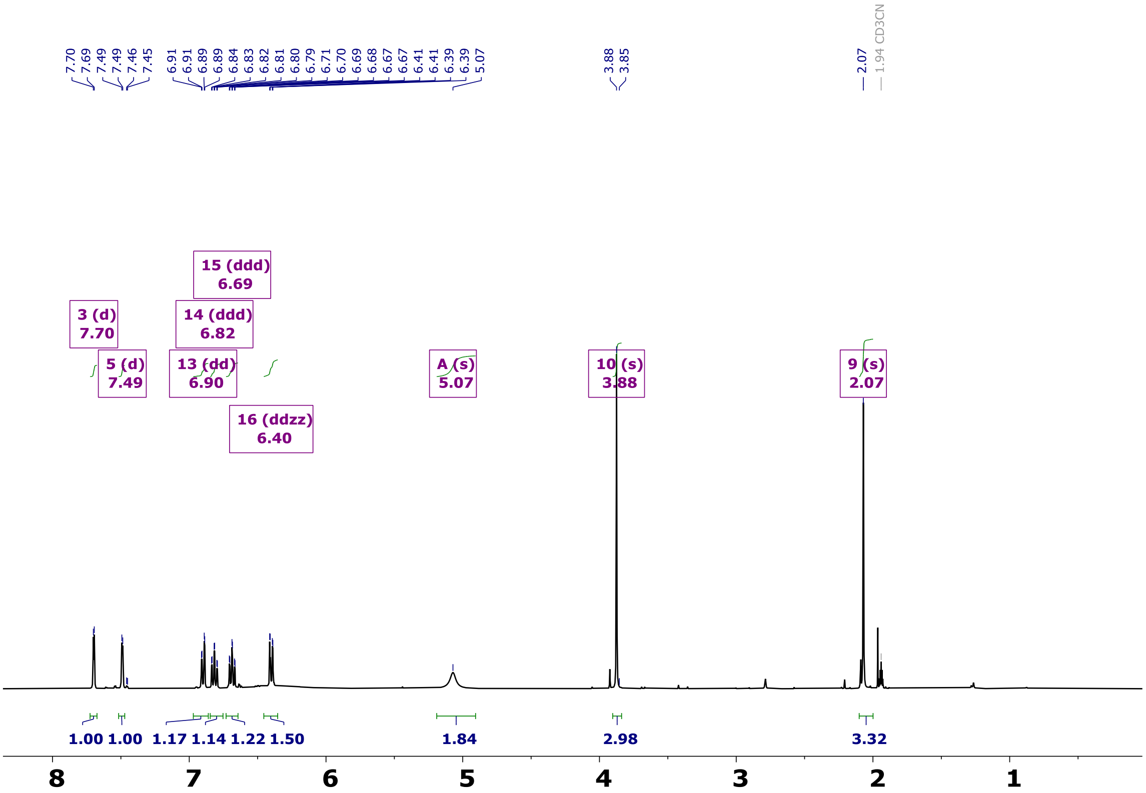

Figure S52: ^{1}H NMR spectrum (CD_3CN, 400 MHz) of **2e**.

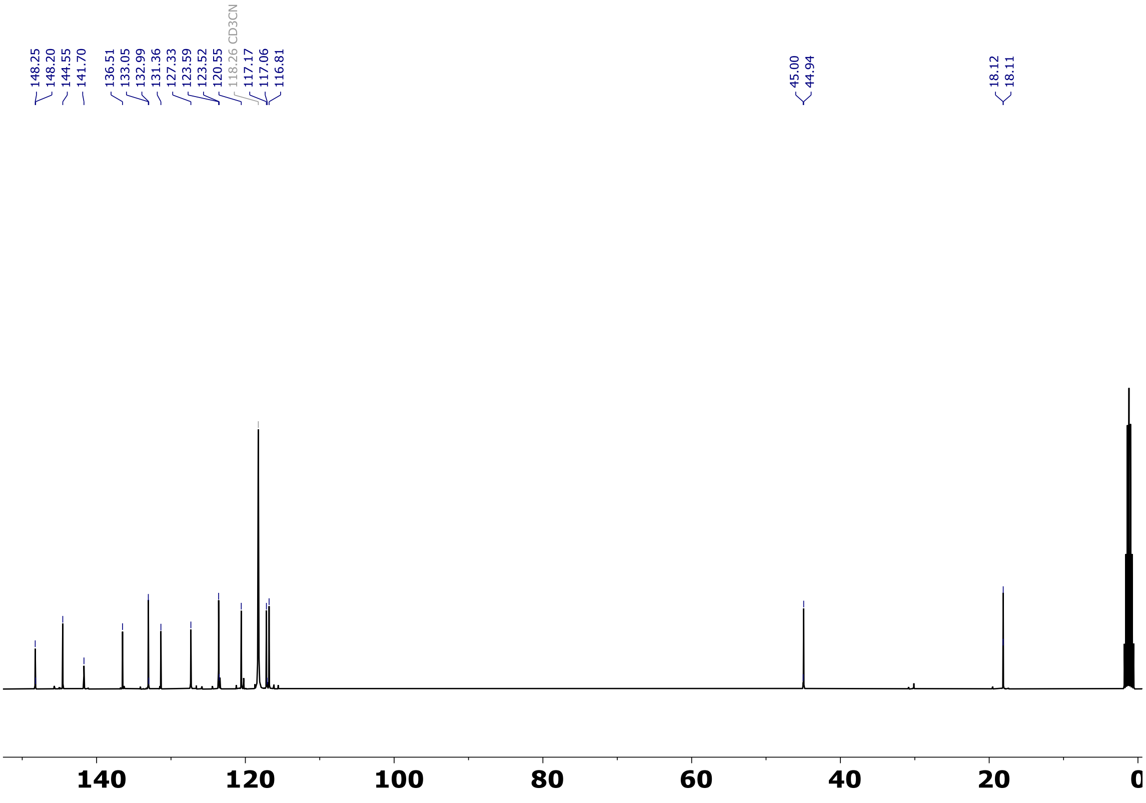

Figure S53: $^{13}C\{^{1}H\}$ NMR spectrum (CD_3CN, 101 MHz) of **2e**.

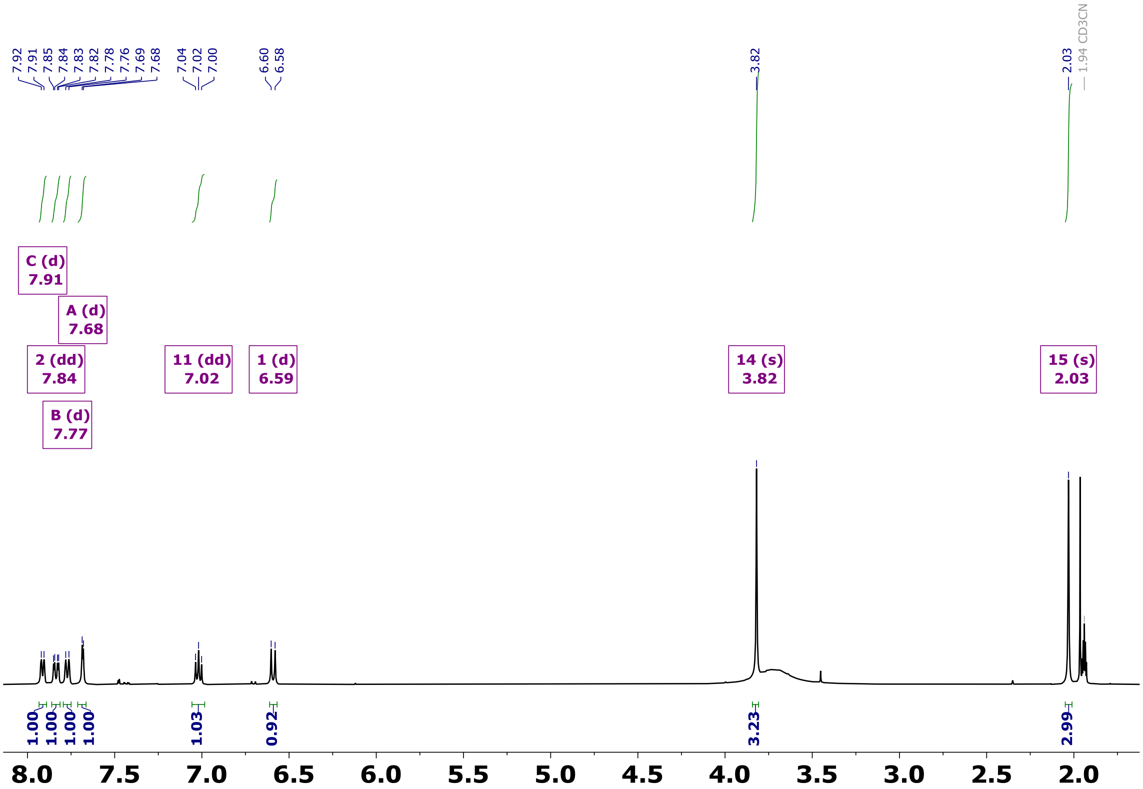

Figure S54: ^{1}H NMR spectrum (CD_3CN, 400 MHz) of **2f**.

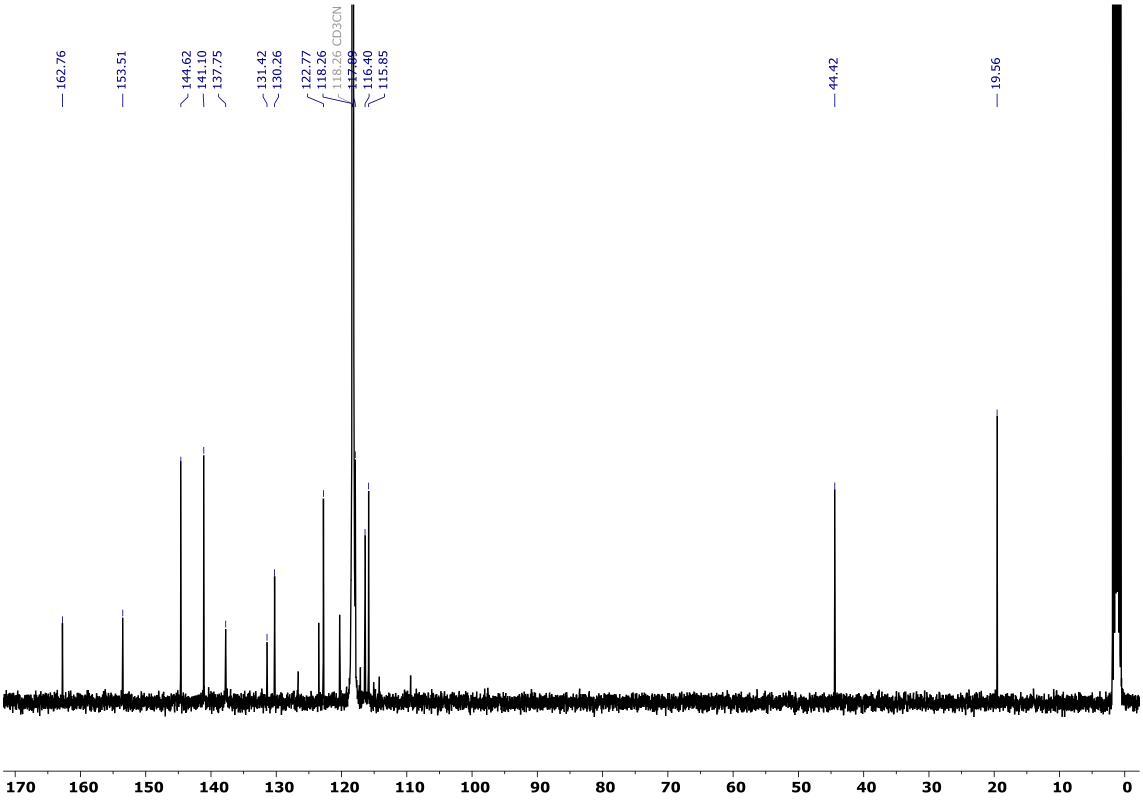

Figure S55: $^{13}C\{^1H\}$ NMR spectrum (CD_3CN, 101 MHz) of **2f**.

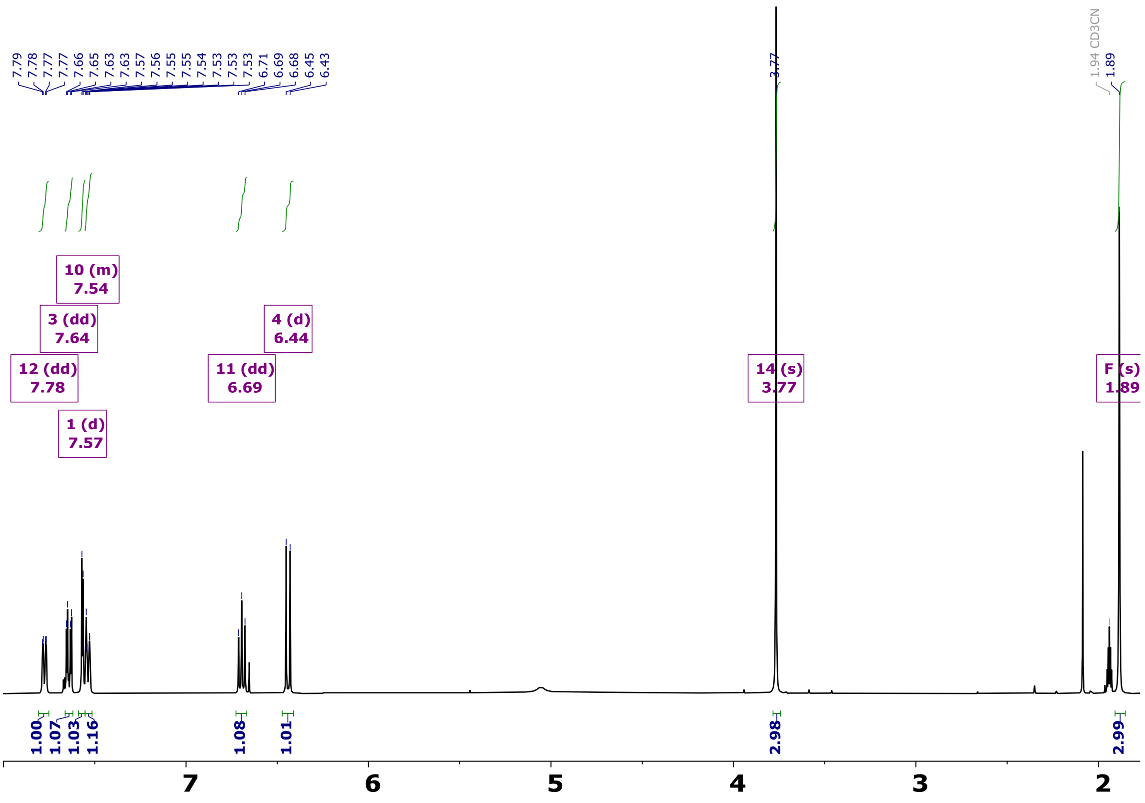

Figure S56: ^{1}H NMR spectrum (CD_3CN, 400 MHz) of **2g**.

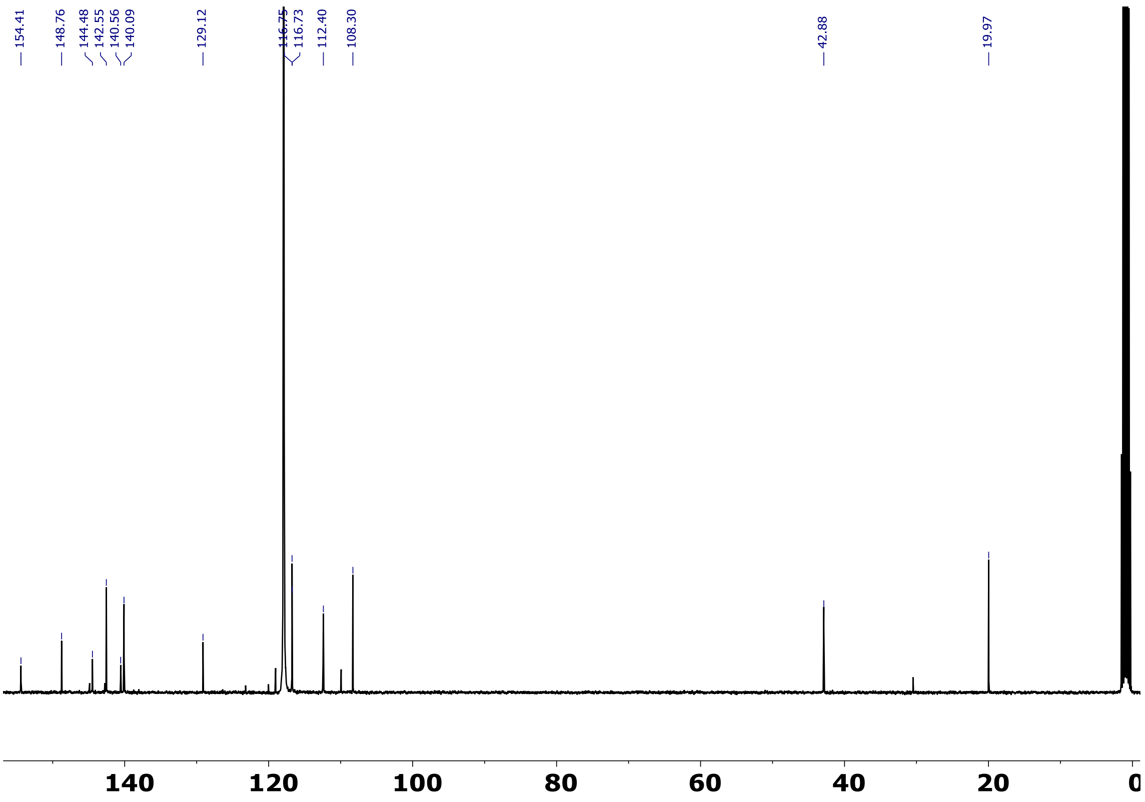

Figure S57: $^{13}C\{^{1}H\}$ NMR spectrum (CD_3CN, 101 MHz) of **2g**.

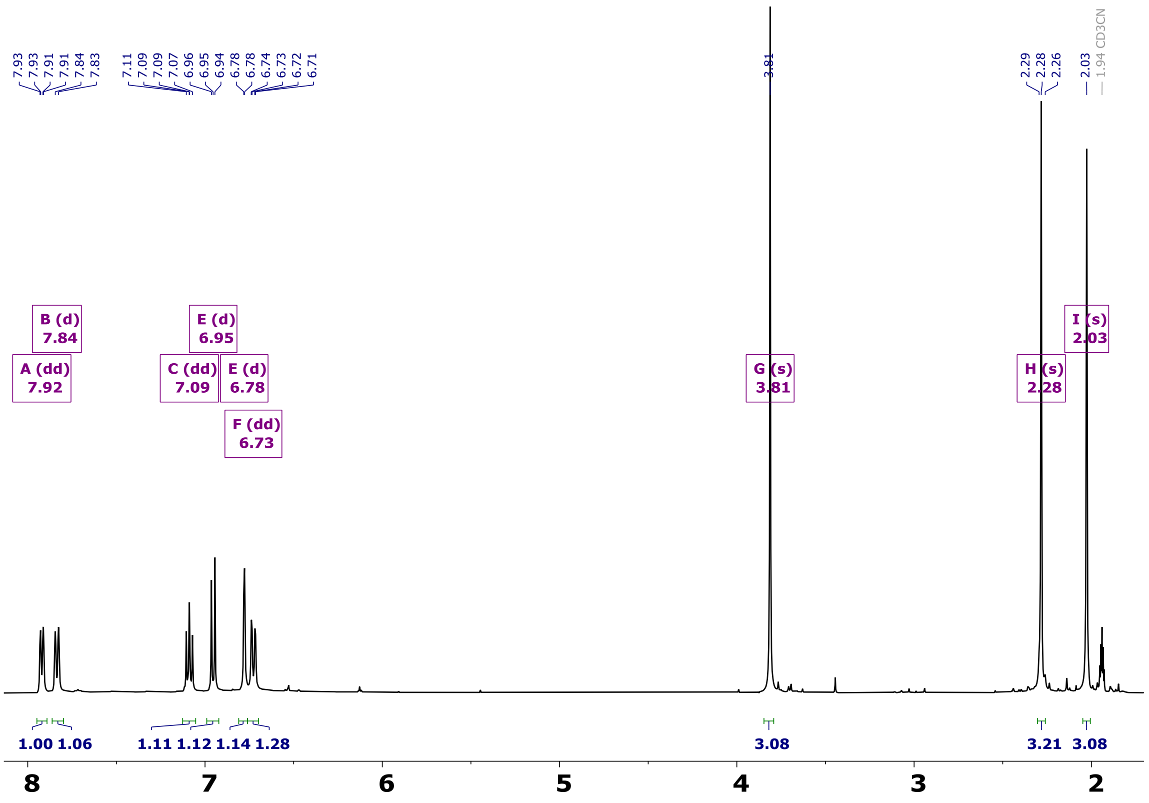

Figure S58: ^{1}H NMR spectrum (CD_3CN, 400 MHz) of **2h**.

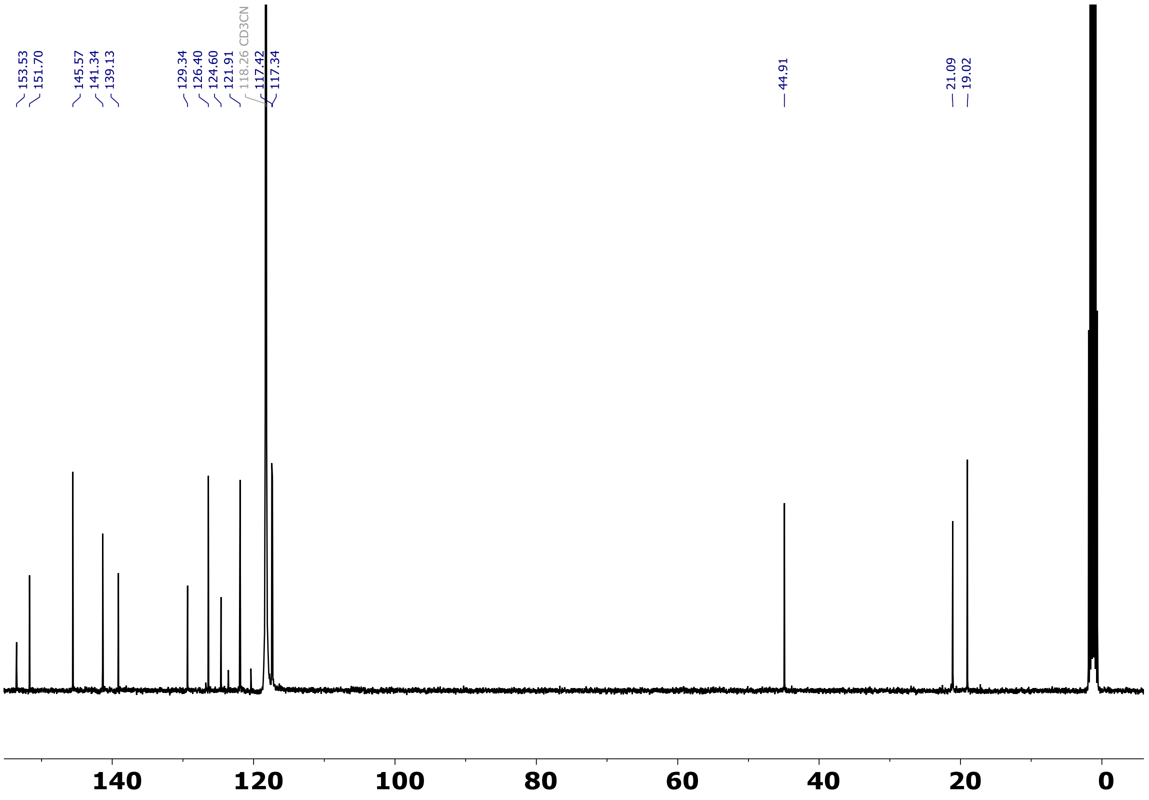

Figure S59: $^{13}C\{^{1}H\}$ NMR spectrum (CD_3CN, 101 MHz) of **2h**.

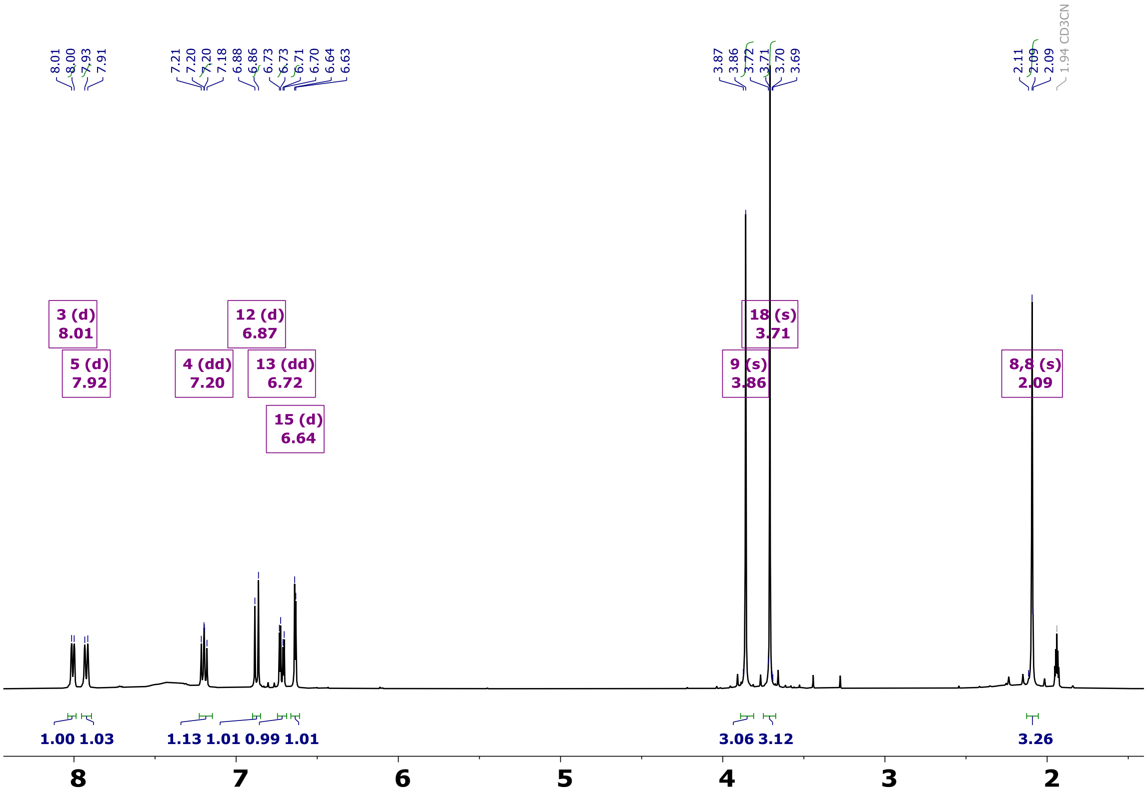

Figure S60: ^{1}H NMR spectrum (CD_3CN, 400 MHz) of **2i**.

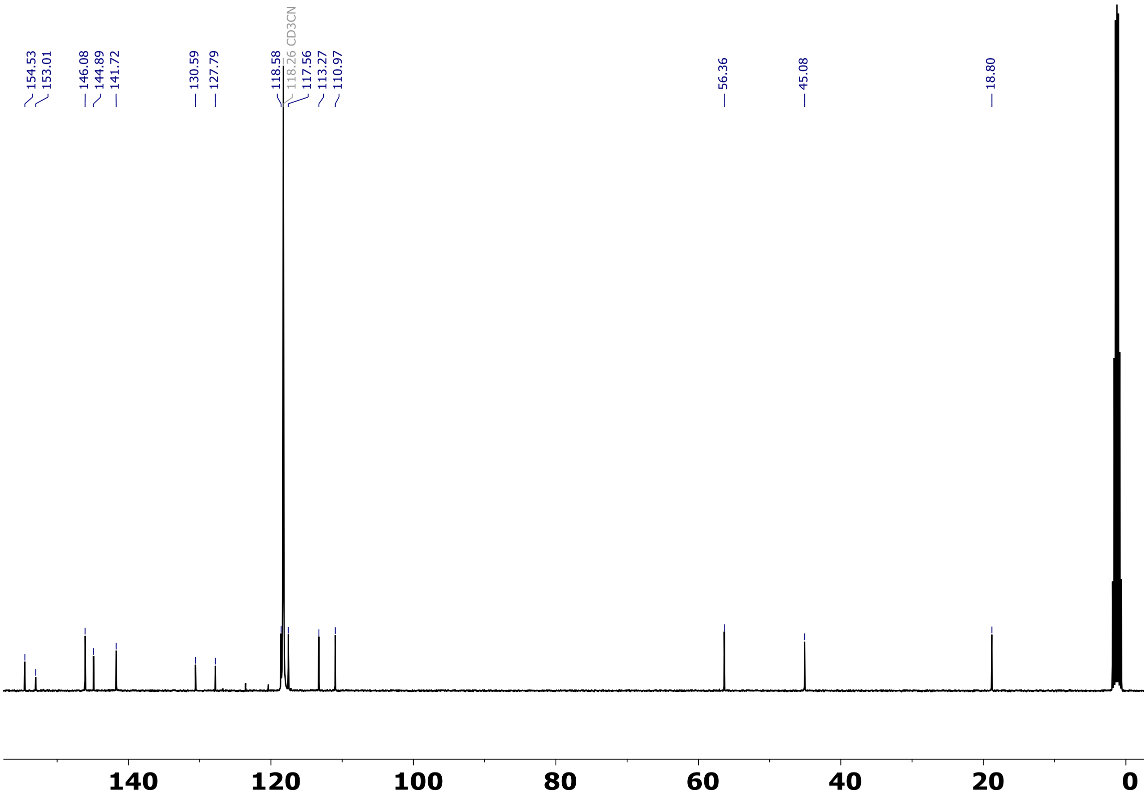

Figure S61: $^{13}C\{^{1}H\}$ NMR spectrum (CD_3CN, 101 MHz) of **2i**.

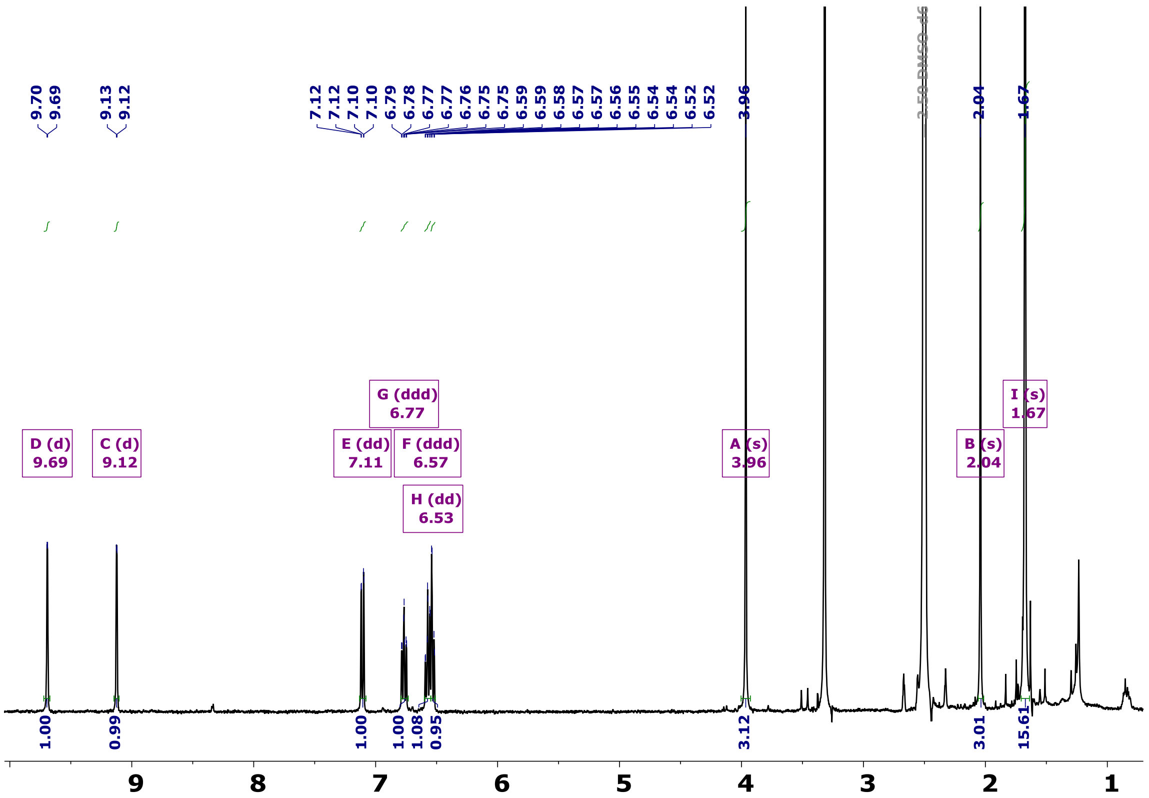

Figure S62: ^{1}H NMR spectrum (DMSO-d$_6$, 400 MHz) of **3b**.

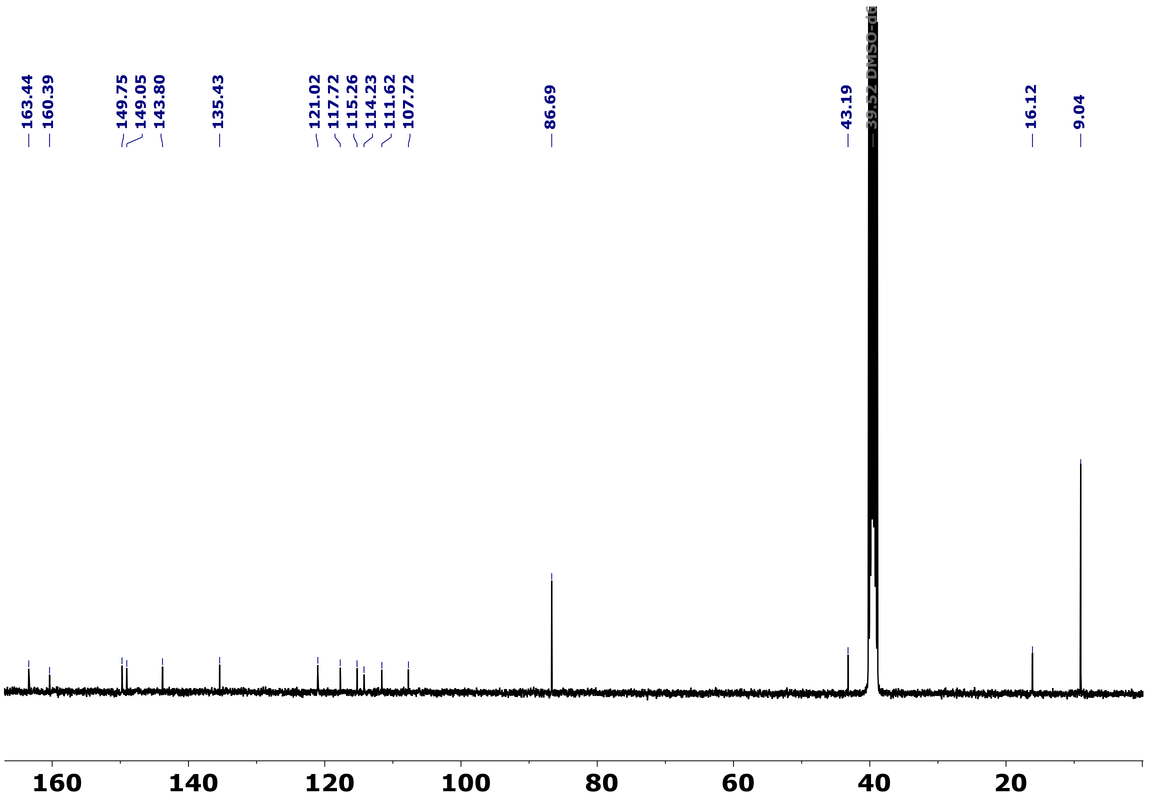

Figure S63: $^{13}C\{^{1}H\}$ NMR spectrum (DMSO-d$_6$, 101 MHz) of **3b**.

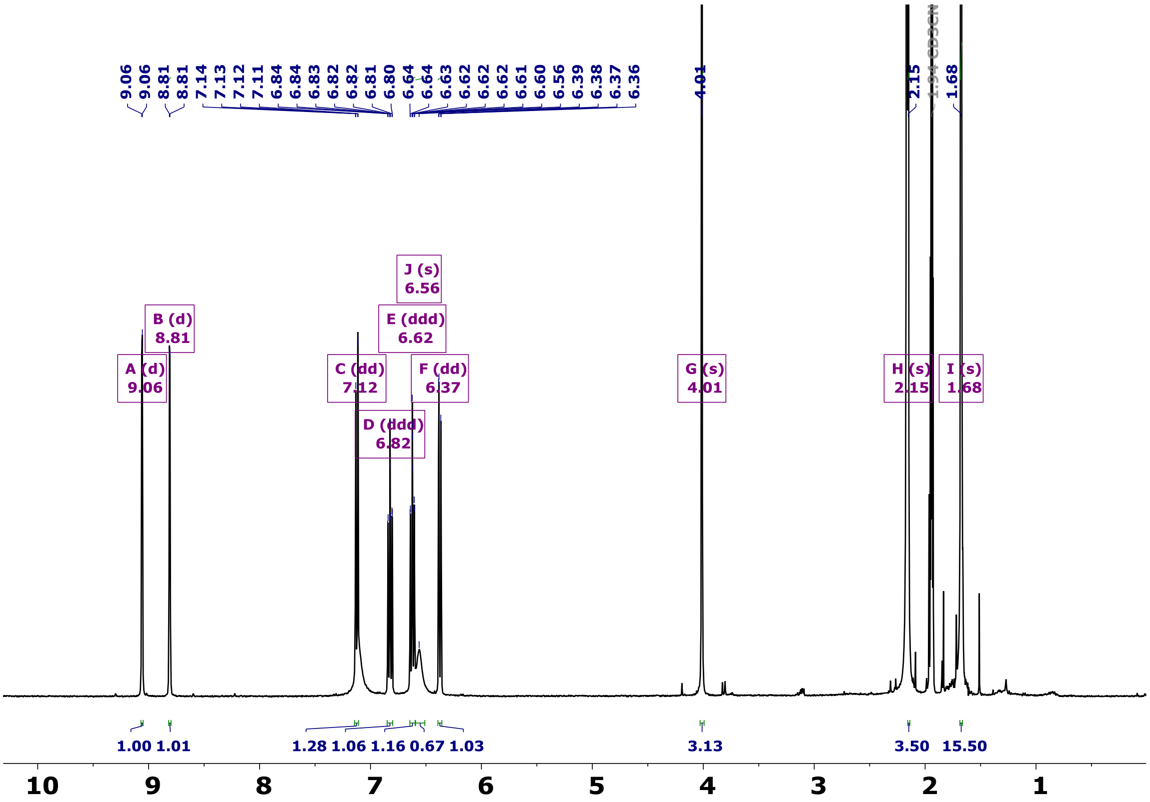

Figure S64: ^{1}H NMR spectrum (CD_3CN, 400 MHz) of **3c**.

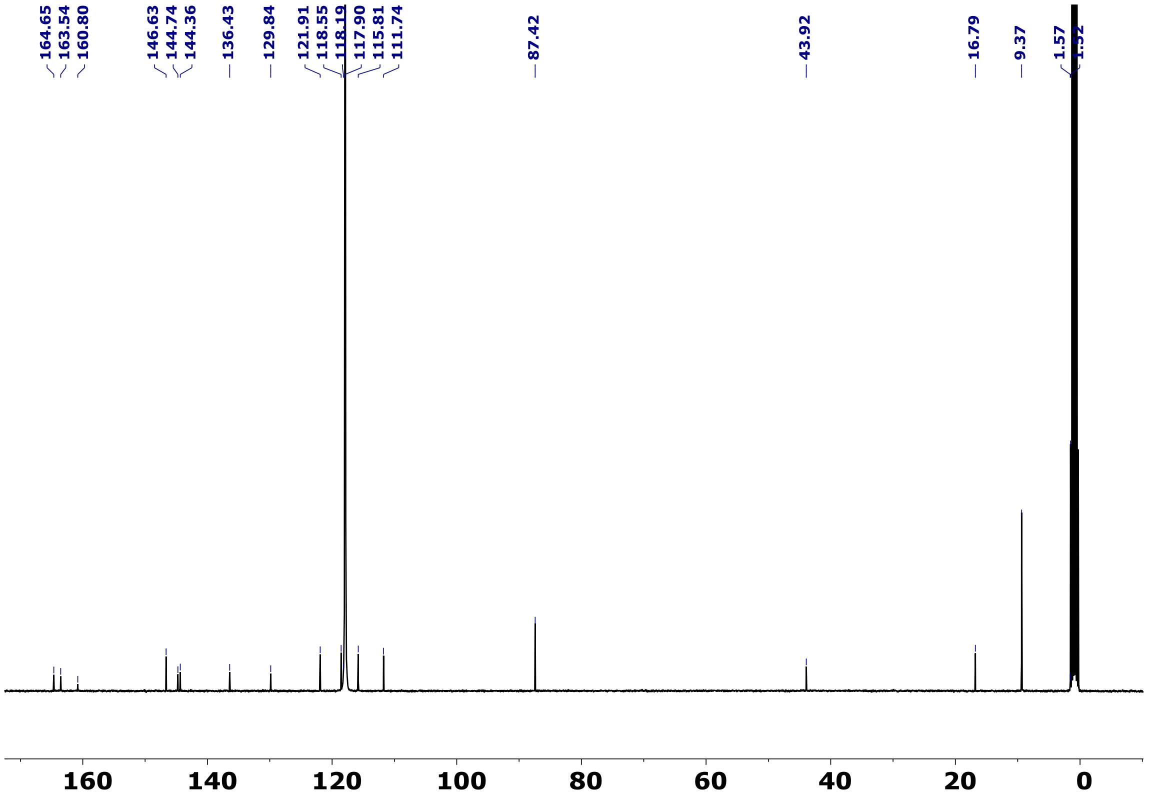

Figure S65: $^{13}C\{^{1}H\}$ NMR spectrum (CD_3CN, 101 MHz) of **3c**.

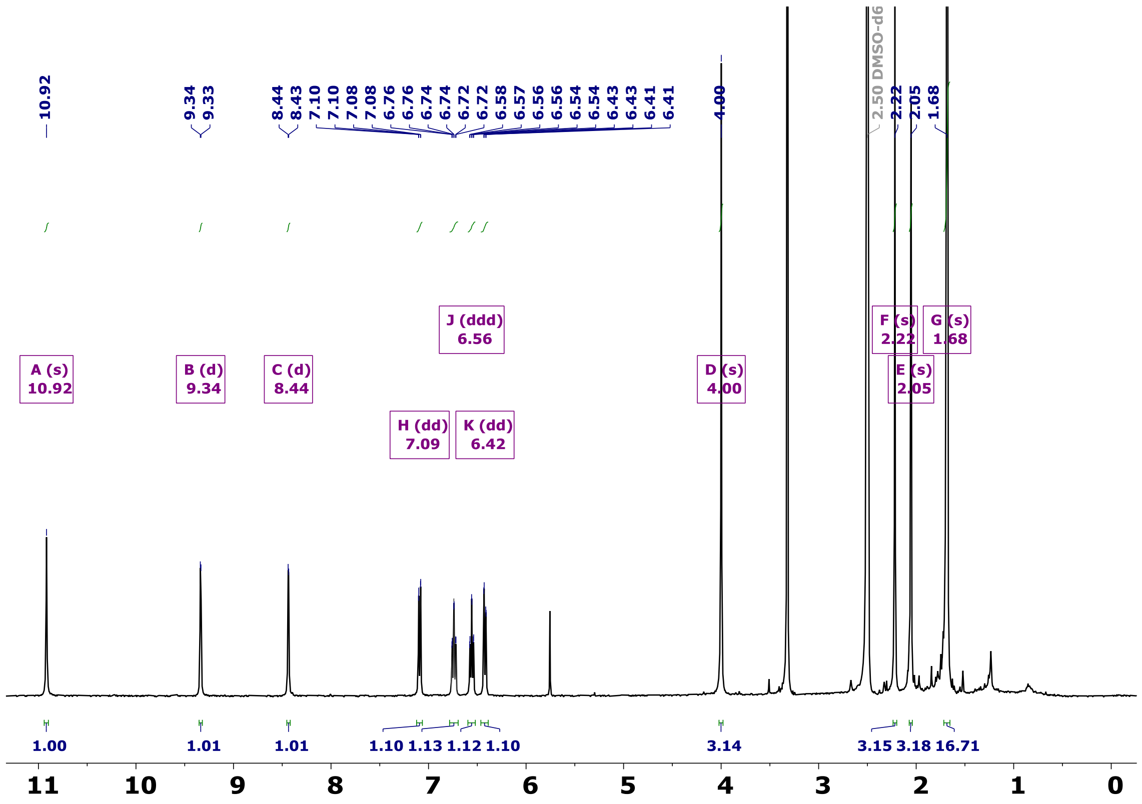

Figure S66: ^{1}H NMR spectrum (DMSO-d$_6$, 400 MHz) of **3d**.

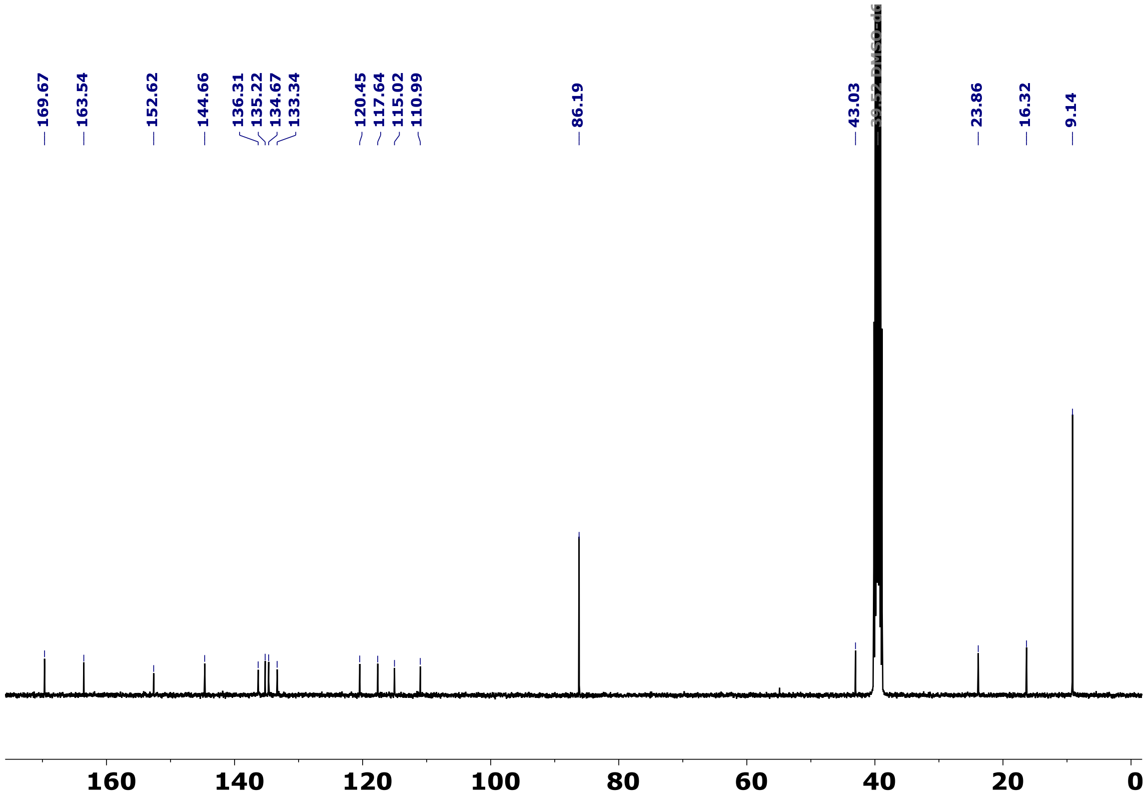

Figure S67: ^{13}C{^{1}H} NMR spectrum (DMSO-d$_6$, 101 MHz) of **3d**.

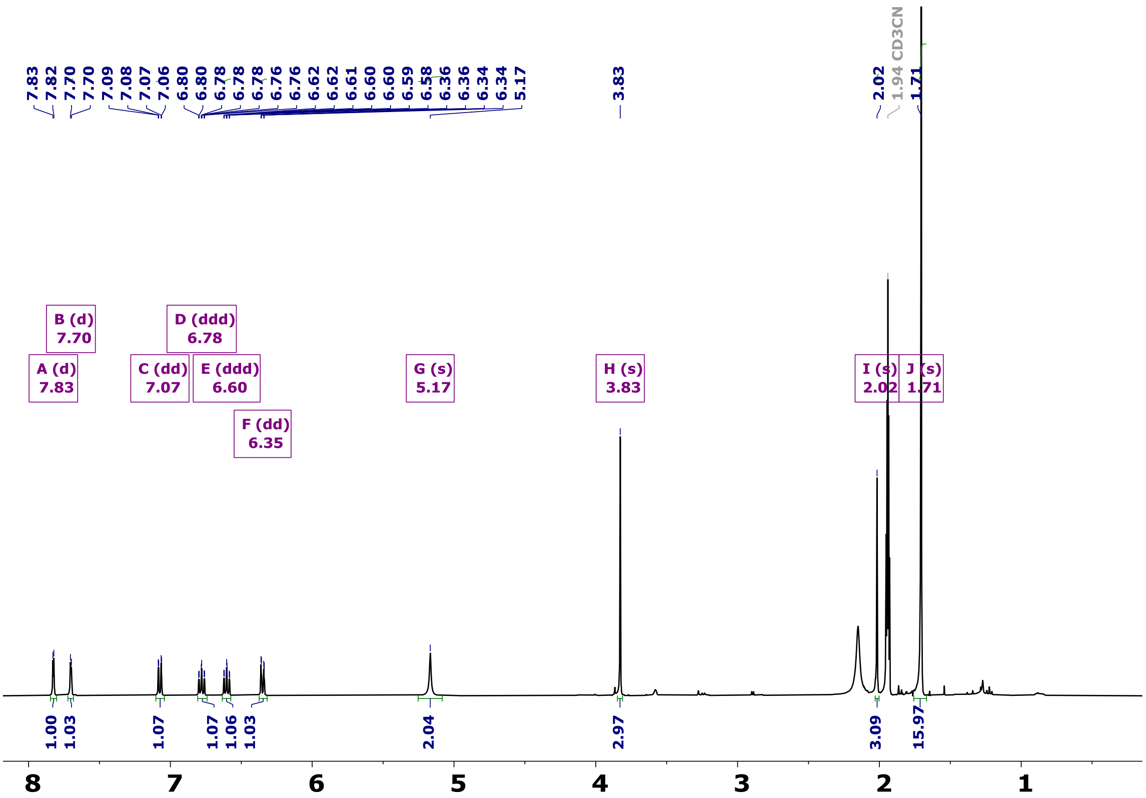

Figure S68: ^{1}H NMR spectrum (CD_3CN, 400 MHz) of **3e**.

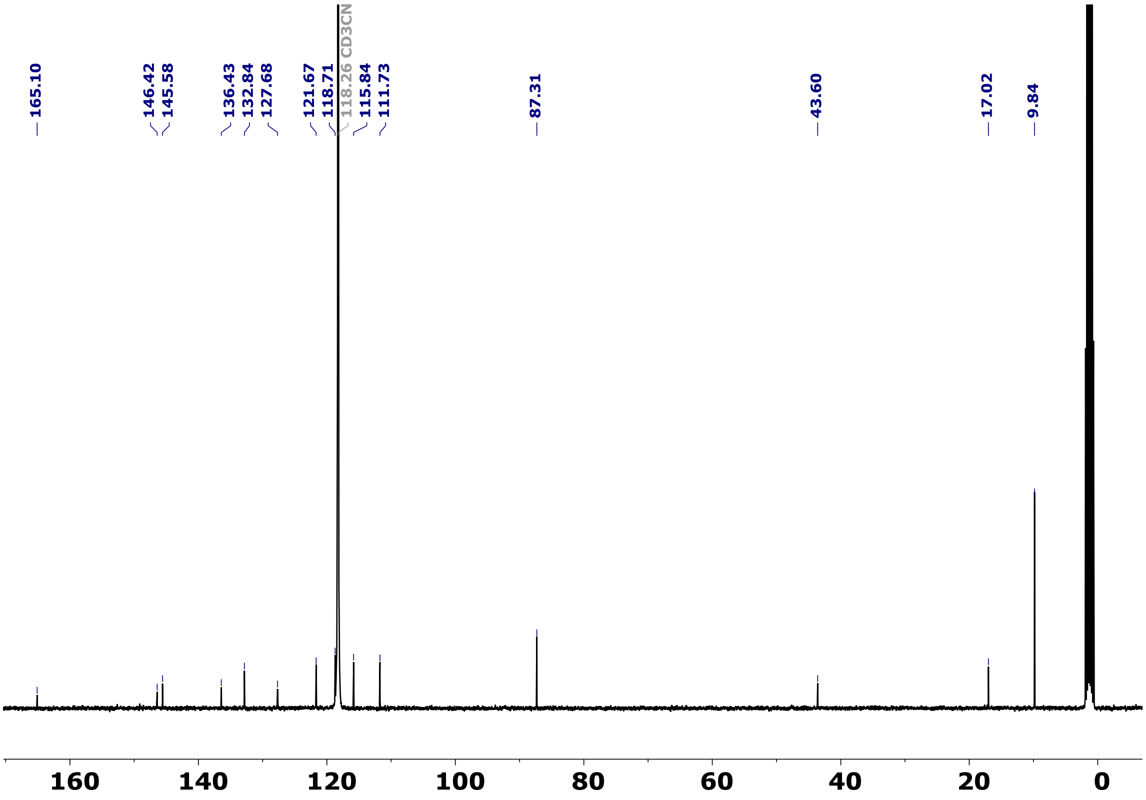

Figure S69: $^{13}C\{^{1}H\}$ NMR spectrum (CD_3CN, 101 MHz) of **3e**.

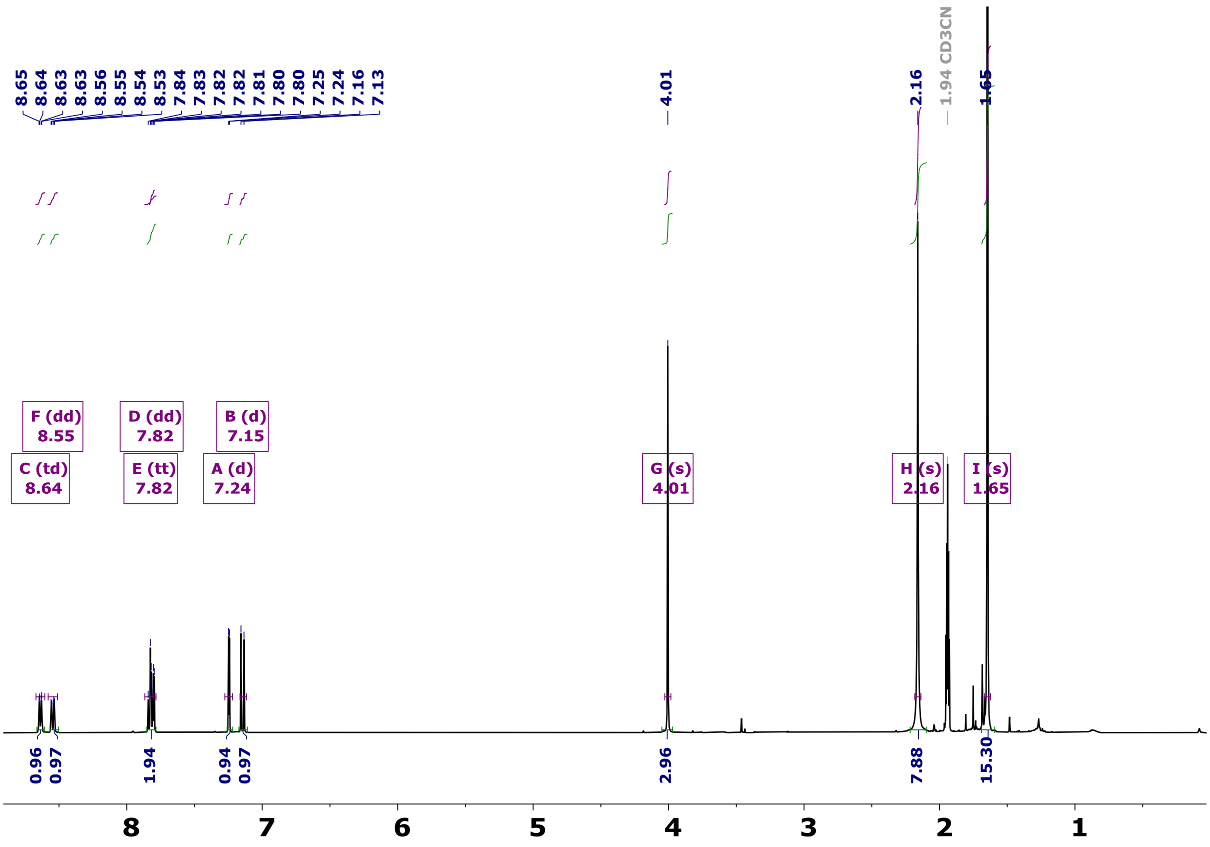

Figure S70: ^{1}H NMR spectrum (CD_3CN, 400 MHz) of **3f**.

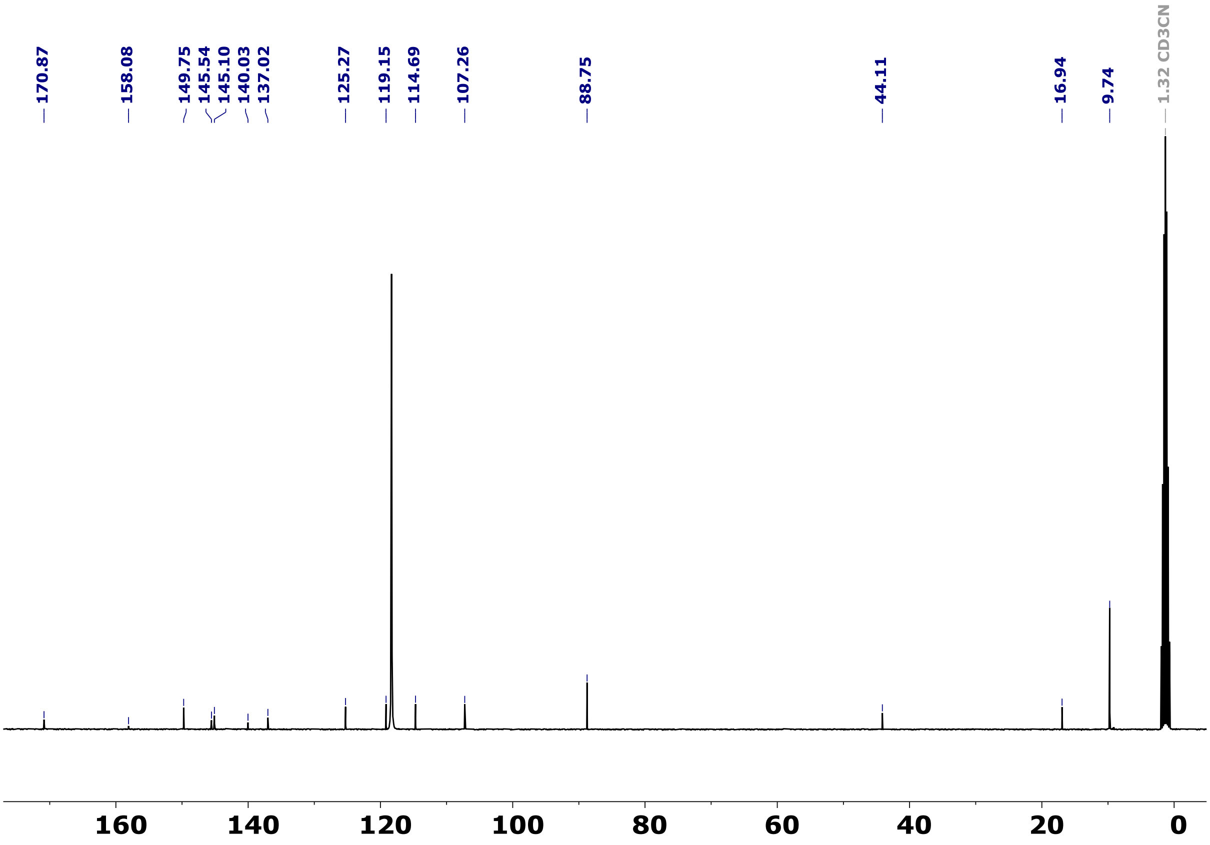

Figure S71: $^{13}C\{^{1}H\}$ NMR spectrum (CD_3CN, 101 MHz) of **3f**.

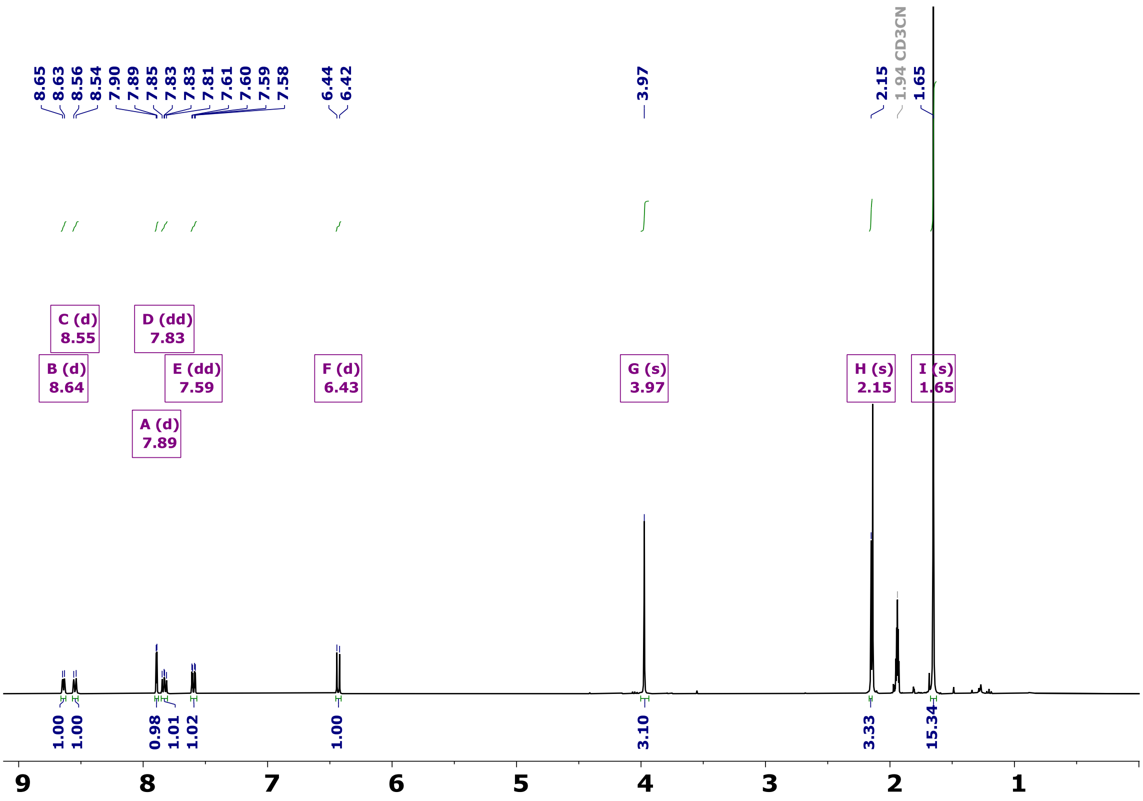

Figure S72: ^{1}H NMR spectrum (CD_3CN, 400 MHz) of **3g**.

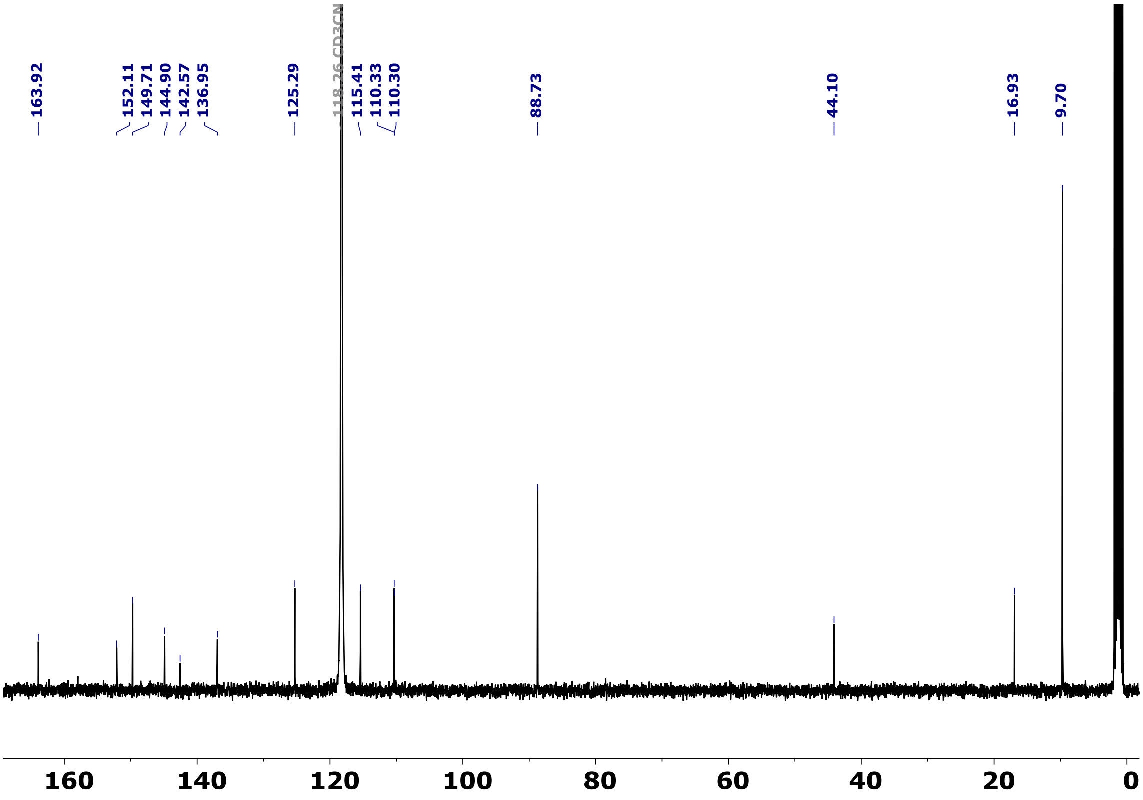

Figure S73: ^{13}C{^{1}H} NMR spectrum (CD_3CN, 101 MHz) of **3g**.

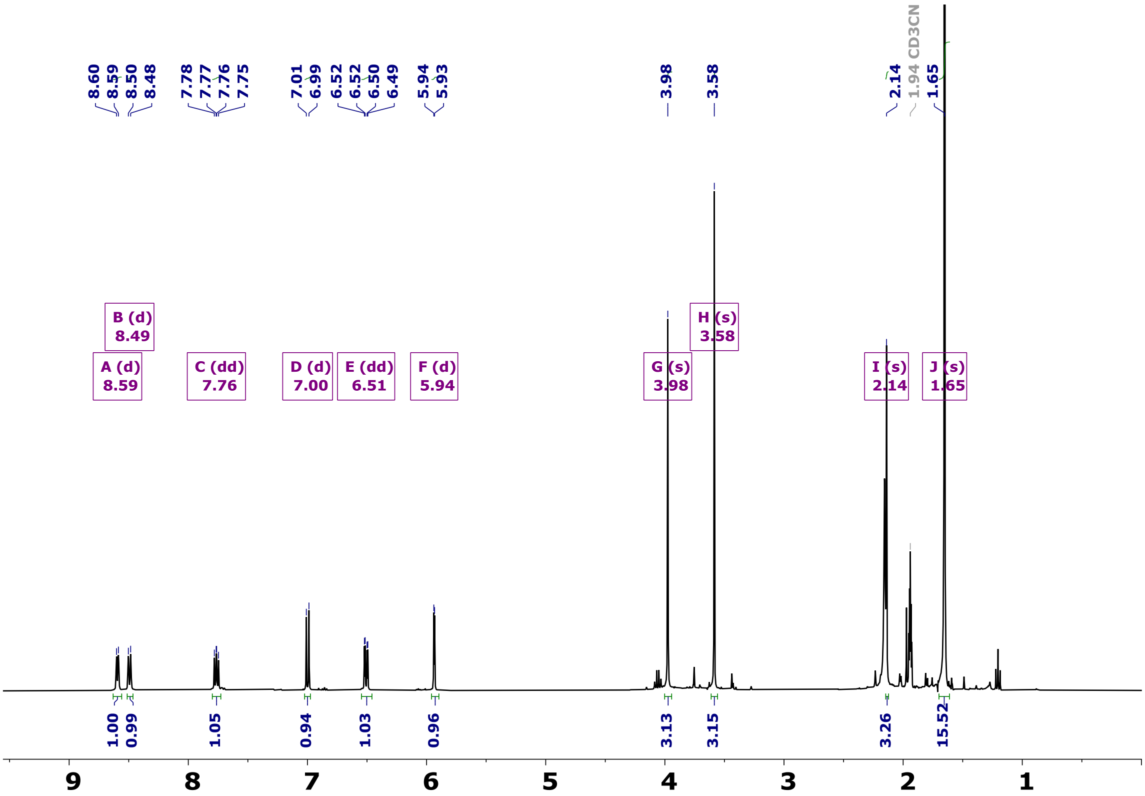

Figure S74: ^{1}H NMR spectrum (CD$_3$CN, 400 MHz) of **3i**.

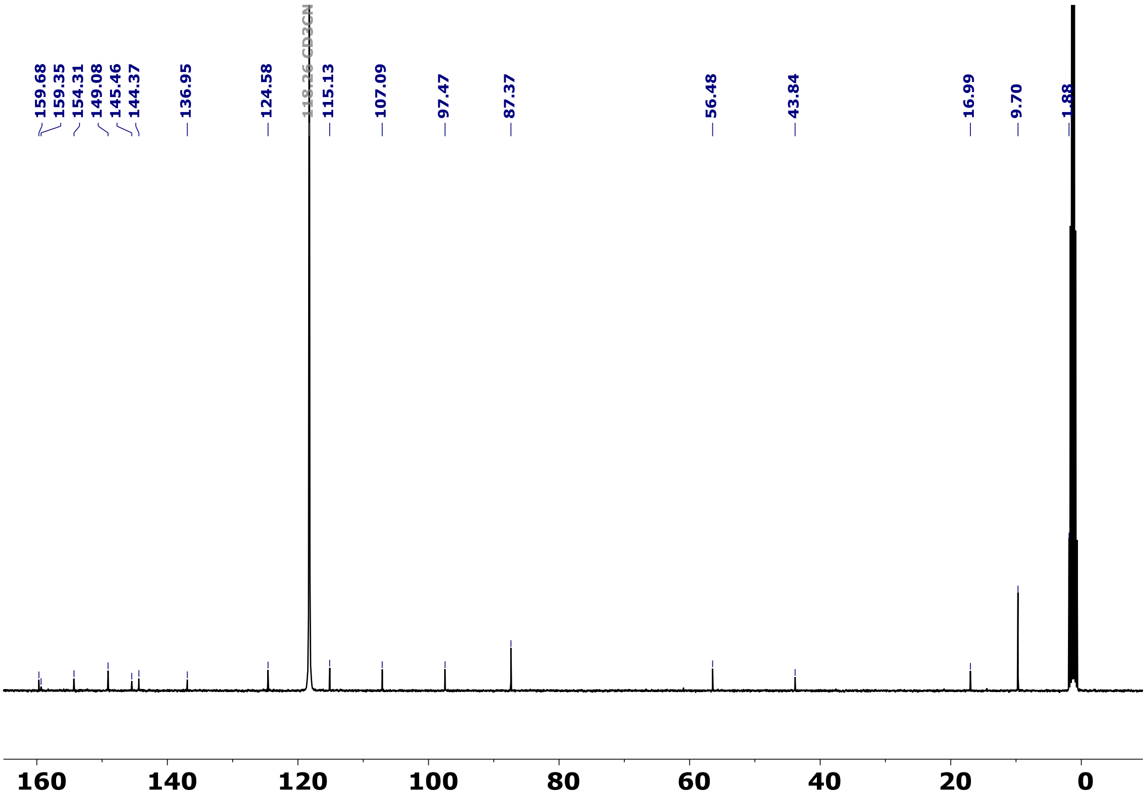

Figure S75: ^{13}C{^{1}H} NMR spectrum (CD$_3$CN, 101 MHz) of **3i**.

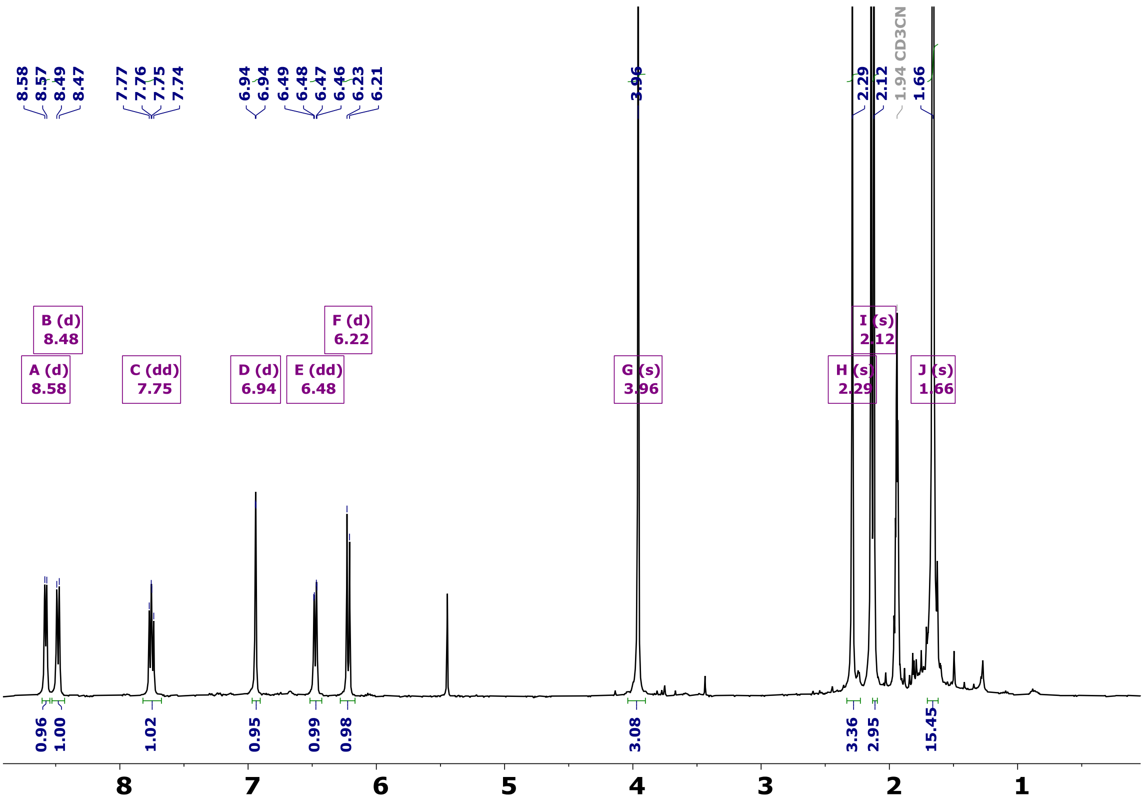

Figure S76: ^{1}H NMR spectrum (CD$_3$CN, 400 MHz) of **3h**.

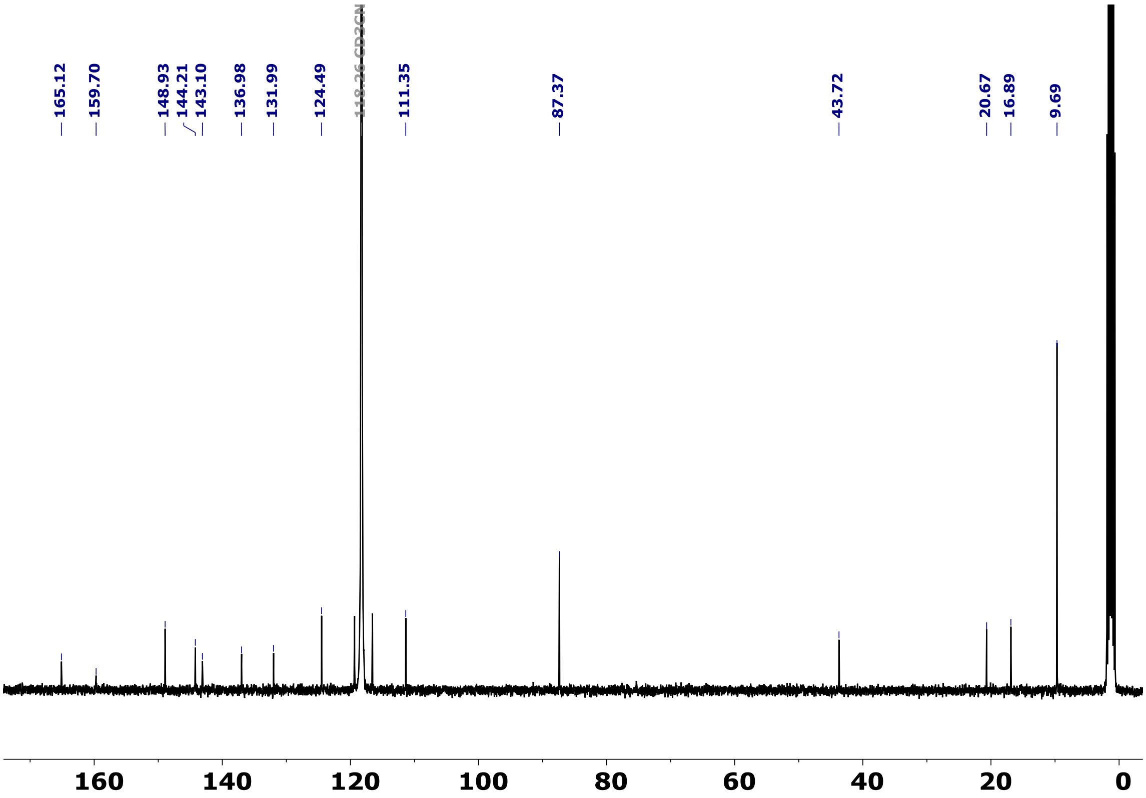

Figure S77: ^{13}C{^{1}H} NMR spectrum (CD$_3$CN, 101 MHz) of **3h**.

9 CRYSTALLOGRAPHY

Crystal-Structure Determination. Suitable single crystals of complexes **3b**, **3g**, and **3i** immersed in parabar oil were mounted at ambient conditions and transferred into a stream of cold nitrogen (173 K). All measurements were carried out on a *RIGAKU XtaLAB Synergy R*, HyPix-Arc100 area-detector diffractometer[S14] using mirror optics monochromated Mo *K*α radiation (λ = 0.71073 Å). The unit cell constants and an orientation matrix for data collection were obtained from a least-squares refinement of the setting angles of reflections in the range 2.10° < θ < 33.51°. Frames were collected using ω scans, with 1.6 seconds exposure time, a rotation angle of 0.5° per frame, a crystal-detector distance of 43.0 mm, at T = 173.0(1) K.

Data reduction was performed using the *CrysAlisPro*[S14] program. The intensities were corrected for Lorentz and polarization effects, and a numerical absorption correction based on gaussian integration over a multifaceted crystal model with additional empirical absorption correction using spherical harmonics using SCALE3 ABSPACK in *CrysAlisPro*[S15] was applied. Data collection and refinement parameters are given in Tables S3–S5.

The structures were solved by intrinsic phasing using *SHELXT*,[S15] which revealed the positions of all non-hydrogen atoms. All non-hydrogen atoms were refined anisotropically. H-atoms were assigned in geometrically calculated positions and refined using a riding model where each H-atom was assigned a fixed isotropic displacement parameter with a value equal to 1.2Ueq of its parent atom (1.5Ueq for methyl groups). The structure of **3g** was refined as an inversion twin. Dynamic disorder of the PF_6 anions was treated with two disorder components for each of the two symmetry-independent anions.

Refinement of the structures was carried out on F^2 using full-matrix least-squares procedures, which minimized the function $\Sigma w(F_o^2 - F_c^2)^2$. The weighting scheme was based on counting statistics and included a factor to downweight the intense reflections. All refinements were performed using the *SHELXL-2014/7*[S16] program in OLEX2.[S17] Crystallographic data for all structures have been deposited with the Cambridge Crystallographic Data entre (CCDC) as supplementary publication number 2555905 (**3b**), 2555904 (**3g**), 2555906 (**3i**).

Table S3. Crystal data and structure refinement for **3b**.

CCDC No	2555905
Empirical formula	$C_{24}H_{27}F_6IrN_3OP$
Formula weight	710.65
Temperature/K	173.00(10)
Crystal system	tetragonal
Space group	$I4_1/a$
a/Å	20.57537(7)
b/Å	20.57537(7)
c/Å	23.89923(13)

α/°	90
β/°	90
γ/°	90
Volume/Å^3	10117.64(9)
Z	16
ρ_{calc}g/cm^3	1.866
μ/mm^{-1}	11.428
F(000)	5536.0
Crystal size/mm^3	0.14 × 0.028 × 0.019
Radiation	Cu Kα (λ = 1.54184)
2Θ range for data collection/°	5.668 to 148.96
Index ranges	–25 ≤ h ≤ 21, –25 ≤ k ≤ 25, –29 ≤ l ≤ 29
Reflections collected	53787
Independent reflections	5198 [R_{int} = 0.0225, R_{sigma} = 0.0110]
Data/restraints/parameters	5198/246/398
Goodness-of-fit on F^2	1.060
Final R indexes [I>=2σ (I)]	R_1 = 0.0271, wR_2 = 0.0561
Final R indexes [all data]	R_1 = 0.0279, wR_2 = 0.0564
Largest diff. peak/hole / e Å^{-3}	0.47/–0.66

Table S4. Crystal data and structure refinement for **3g**.

CCDC No.	2555904
Empirical formula	$C_{23}H_{27}N_3O_3F_6PIr$
Formula weight	730.64
Temperature/K	173.00(10)
Crystal system	orthorhombic
Space group	Pna2$_1$
a/Å	20.0863(2)
b/Å	7.44160(10)
c/Å	34.1259(4)
α/°	90
β/°	90
γ/°	90
Volume/Å^3	5100.94(10)
Z	8
ρ_{calc}g/cm^3	1.903
μ/mm^{-1}	5.373
F(000)	2848.0
Crystal size/mm^3	0.43 × 0.198 × 0.118
Radiation	Mo Kα (λ = 0.71073)
2Θ range for data collection/°	4.228 to 61.012
Index ranges	–28 ≤ h ≤ 28, –10 ≤ k ≤ 10, –48 ≤ l ≤ 48
Reflections collected	142289
Independent reflections	15596 [R_{int} = 0.0396, R_{sigma} = 0.0192]
Data/restraints/parameters	15596/360/792
Goodness-of-fit on F^2	1.033
Final R indexes [I>=2σ (I)]	R_1 = 0.0240, wR_2 = 0.0605
Final R indexes [all data]	R_1 = 0.0265, wR_2 = 0.0614
Largest diff. peak/hole / e Å^{-3}	1.54/–0.71
Flack parameter	0.468(6)

Table S5. Crystal data and structure refinement for **3i**.

CCDC No.	2555906
Empirical formula	$C_{24}H_{30}F_6IrN_2O_2P$
Formula weight	715.67
Temperature/K	173.00(10)
Crystal system	monoclinic
Space group	$P2_1/n$
a/Å	9.16248(11)
b/Å	7.96837(9)
c/Å	34.6978(5)
α/°	90
β/°	93.3315(11)
γ/°	90
Volume/Å^3	2529.00(5)
Z	4
ρ_{calc}g/cm^3	1.880
μ/mm^{-1}	5.412
F(000)	1400.0
Crystal size/mm^3	0.145 × 0.121 × 0.012
Radiation	Mo Kα (λ = 0.71073)
2Θ range for data collection/°	4.54 to 61.014
Index ranges	$-13 \le h \le 13$, $-11 \le k \le 11$, $-49 \le l \le 49$
Reflections collected	75638
Independent reflections	7729 [R_{int} = 0.0244, R_{sigma} = 0.0138]
Data/restraints/parameters	7729/169/409
Goodness-of-fit on F^2	1.274
Final R indexes [I>=2σ (I)]	R_1 = 0.0248, wR_2 = 0.0479
Final R indexes [all data]	R_1 = 0.0287, wR_2 = 0.0487
Largest diff. peak/hole / e Å^{-3}	1.03/–0.88

10 References

S1 N. Lentz and M. Albrecht, *ACS Catal.*, 2022, **12**, 12627–12631.

S2 N. Lentz, S. Reuge and M. Albrecht, *ACS Catal.*, 2023, **13**, 9839–9844.

S3 N. Lentz, S. Reuge, A. Beaufils and M. Albrecht, *Organometallics*, 2024, **43**, 1536–1546.

S4 F. Neese, *WIREs Comput. Mol. Sci.*, 2025, **15**, e70019.

S5 C. Lee, W. Yang and R. G. Parr, *Phys. Rev. B*, 1988, **37**, 785–789.

S6 A. D. Becke, *J. Chem. Phys.*, 1993, **98**, 5648–5652.

S7 S. Grimme, S. Ehrlich and L. Goerigk, *J. Comput. Chem.*, 2011, **32**, 1456–1465.

S8 S. Grimme, J. Antony, S. Ehrlich and H. Krieg, *J. Chem. Phys.*, 2010, **132**, 154104.

S9 F. Weigend and R. Ahlrichs, *Phys. Chem. Chem. Phys.*, 2005, **7**, 3297–3305.

S10 S. Kossmann and F. Neese, *J. Chem. Theory Comput.*, 2010, **6**, 2325–2338.

S11 Y. Takano and K. N. Houk, *J. Chem. Theory Comput.*, 2005, **1**, 70–77.

S12 V. Barone and M. Cossi, *J. Phys. Chem. A*, 1998, **102**, 1995–2001.

S13 G. Bruhn, E. R. Davidson, I. Mayer and A. E. Clark, *Int. J. Quantum Chem.*, 2006, **106**, 2065–2072.

S14 *CrysAlis PRO*, Version 1.171.40.37a, Oxford Diffraction Ltd, Yarnton, UK, 2018.

S15 G. M. Sheldrick, *Acta Crystallogr. A Found. Adv.*, 2015, **71**, 3–8.

S16 G. M. Sheldrick, *Acta Crystallogr. C Struct. Chem.*, 2015, **71**, 3–8.

S17 O. V. Dolomanov, L. J. Bourhis, R. J. Gildea, J. A. K. Howard and H. Puschmann, *J. Appl. Crystallogr.*, 2009, **42**, 339–341.
